# Supplementary material for: Building knowledge, optimising physical and mental health and setting up healthier life trajectories in South African women (Bukhali): a preconception randomised control trial part of the Healthy Life Trajectories Initiative (HeLTI)
Source: BMJ Open. 2022 Apr 21;12(4):e059914. doi: 10.1136/bmjopen-2021-059914 (PMC9024255; doi:10.1136/bmjopen-2021-059914)
Supplement: Supplementary data [file bmjopen-2021-059914supp001.pdf]

Table 1: World Health Organization Trial Registration Data Set

**Data category**	**Information**
Primary registry and trial identifying number	PACTR201903750173871
Date of registration in primary registry	25 March 2019
Source(s) of monetary or material support	South African Medical Research Council; Canadian Institutes of Health Research
Primary sponsor	University of the Witwatersrand, Johannesburg, South Africa
Contact for public queries	SAN; shane.norris@wits.ac.za
Contact for scientific queries	SAN; shane.norris@wits.ac.za
Public title	HeLTI-SA: a preconception randomised control trial
Countries of recruitment	South Africa
Health condition(s) or problem(s) studied	Child obesity and non-communicable disease risk
Intervention(s)	Active comparator: community health worker, health literacy, behaviour change, dietetic and counselling support; risk-screening health services; multi-micronutrient supplement<br>Placebo comparator: standard of care plus non-health related call centre support
Key inclusion and exclusion criteria	Women aged 18-28 years are eligible, unless they are: (i) diagnosed with type-I diabetes or epilepsy because these require intensive treatment and management priorities; (ii) present with intellectual disability that hinders informed consent, and (iii), not able or willing to provide consent.
Study type	Complex prevention intervention; individual randomisation; research team blind to randomisation; phase II
Date of first enrolment	October 2019
Target sample size	6800 women
Recruitment status	Recruiting
Primary outcome(s)	DXA-derived fat mass index (fat mass/height^2)
Key secondary outcomes	Maternal and child anthropometry, cardiometabolic, and behaviour, and child developmental outcomes
