# Supplementary material for: Building knowledge, optimising physical and mental health and setting up healthier life trajectories in South African women (Bukhali): a preconception randomised control trial part of the Healthy Life Trajectories Initiative (HeLTI)
Source: BMJ Open. 2022 Apr 21;12(4):e059914. doi: 10.1136/bmjopen-2021-059914 (PMC9024255; doi:10.1136/bmjopen-2021-059914)
Supplement: Author's manuscript [file bmjopen-2021-059914.draft_revisions.pdf]

BMJ Open is committed to open peer review. As part of this commitment we make the peer review history of every article we publish publicly available.

When an article is published we post the peer reviewers' comments and the authors' responses online. We also post the versions of the paper that were used during peer review. These are the versions that the peer review comments apply to.

The versions of the paper that follow are the versions that were submitted during the peer review process. They are not the versions of record or the final published versions. They should not be cited or distributed as the published version of this manuscript.

BMJ Open is an open access journal and the full, final, typeset and author-corrected version of record of the manuscript is available on our site with no access controls, subscription charges or pay-per-view fees (http://bmjopen.bmj.com).

If you have any questions on BMJ Open's open peer review process please email info.bmjopen@bmj.com

BMJ Open

Building knowledge, optimising physical and mental health, and setting up healthier life trajectories in South African women (Bukhali): a preconception randomised control trial part of the Healthy Life Trajectories Initiative (HeLTI)

Journal:	*BMJ Open*
Manuscript ID	bmjopen-2021-059914
Article Type:	Protocol
Date Submitted by the Author:	07-Dec-2021

Complete List of Authors:	Norris, Shane; University of the Witwatersrand, SAMRC Developmental Pathways for Health Research Unit, Department of Paediatrics; University of Southampton, Global Health Research Institute, School of Health and Human Development<br>Draper, Catherine; University of the Witwatersrand, Department of Paediatrics<br>Prioreschi, Alessandra; University of the Witwatersrand, MRC/Wits Developmental Pathways for Health Research Unit<br>Smuts, CM; North-West University<br>Ware, Lisa; University of the Witwatersrand, Department of Paediatrics<br>Dennis, CindyLee; University of Toronto<br>Awadalla, Philip; University of Toronto, Department of Molecular Genetics, Ontario Institute for Cancer Research<br>Bassani, D; The Hospital for Sick Children<br>Bhutta, Zulfiqar; The Hospital for Sick Children<br>Briollais, Laurent; University of Cape Town<br>Cameron, D; University of Ottawa<br>Chirwa, Tobias; University of the Witwatersrand<br>Fallon, B; University of Toronto<br>Gray, CM; University of Stellenbosch, Division of Molecular Biology and Human Genetics; University of Cape Town, Division of Immunology, Institute of Infectious Disease and Molecular Medicine<br>Hamilton , Jill; University of Toronto, Department of Paediatrics<br>Jamison, J; Red River College<br>Jaspan, Heather; University of Cape Town, Division of Immunology, Institute of Infectious Disease and Molecular Medicine<br>Jenkins, Jennifer; University of Toronto, Department of Applied Psychology and Human Development<br>Kahn, Kathleen; University of the Witwatersrand<br>Kengne , AP; South African Medical Research Council<br>Lambert, Estelle; University of Cape Town, Division of Exercise Science and Sports Medicine<br>Levitt, Naomi; University of Cape Town<br>Martin , Marie-Claude ; University of Toronto<br>Ramsay, Michele; University of the Witwatersrand<br>Roth, Daniel; The Hospital for Sick Children; University of Toronto, Department of Paediatrics<br>Scherer, Stephen; University of Toronto, Department of Molecular

	Genetics; The Hospital for Sick Children<br>Sellen, Daniel; University of Toronto; University of Toronto, Department of Nutritional Sciences<br>Slemming, Wiedaad; University of the Witwatersrand, Department of Paediatrics<br>Sloboda, Deborah; Hamilton, Department of Biochemistry and Biomedical Sciences<br>Szyf, M.; McGill University, Department of Pharmacology and Therapeutics<br>Tollman, Stephen; University of the Witwatersrand<br>Tomlinson, Mark; University of Stellenbosch, Institute for Life Course Health Research; Queen's University Belfast<br>Tough, Suzanne ; Alberta Children's Hospital Research Institute, Department of Paediatrics<br>Matthews, Stephen; University of Toronto, Department of Applied Psychology and Human Development; University of Toronto<br>Richter, Linda; University of the Witwatersrand<br>Lye, Stephen; University of Toronto, Department of Applied Psychology and Human Development; University of Toronto
Keywords:	NUTRITION & DIETETICS, PUBLIC HEALTH, SOCIAL MEDICINE

SCHOLARONE™
Manuscripts

1

Building knowledge, optimising physical and mental health, and setting up healthier life trajectories in South African women (Bukhali): a preconception randomised control trial part of the Healthy Life Trajectories Initiative (HeLTI)

Norris SA[1], Draper CD[1], Prioireschi A[1], Smuts CM[2], , Ware L[1,28], Dennis CL[3], Awadalla P[4], Bassani DG[5], Bhutta ZA[5,6], Briollais L[7,19], Cameron B[8], Chirwa T[9], Fallon B[10], Gray CM[11,30], Hamilton J[12], Jamison J[13], Jaspan H[11], Jenkins JM[14], Kahn K[15], Kenge AP[16], Lambert E[17], Levitt N[18], Martin MC[19], Ramsay M[20], Roth, DE[5,12], Scherer SW[4,21], Sellen D[7,22], Slemming W[23], Sloboda DM[24], Szyf M[25], Tollman S[15], Tomlinson M[26,31], Tough S[27], Matthews S[14,19,28], Richter LM[29], Lye S[14,19,28]

Affiliations

1. SAMRC Developmental Pathways for Health Research Unit, Department of Paediatrics, Faculty of Health Sciences, University of the Witwatersrand, Johannesburg, South Africa
2. Centre of Excellence for Nutrition, North-West University, Potchefstroom, South Africa
3. Lawrence S. Bloomberg Faculty of Nursing, University of Toronto, Toronto, Canada
4. Department of Molecular Genetics, Ontario Institute for Cancer Research, University of Toronto, Toronto, Canada
5. Centre for Global Child Health, The Hospital for Sick Children, Toronto, Canada
6. Institute for Global Health and Development, Aga Khan University, Karachi, Pakistan
7. Dalla Lana School of Public Health, University of Toronto, Toronto, Canada
8. Ottawa Hospital Research Institute, University of Ottawa, Ottawa, Canada
9. School of Public Health, University of the Witwatersrand, Johannesburg, South Africa
10. Factor-Inwentash Faculty of Social Work, University of Toronto, Toronto, Canada
11. Division of Immunology, Institute of Infectious Disease and Molecular Medicine, Faculty of Health Sciences, University of Cape Town, Cape Town, South Africa
12. Department of Paediatrics, The Hospital for Sick Children, University of Toronto, Toronto, Canada
13. School of Health Sciences and Community Services, Red River College, Winnipeg, Canada
14. Department of Applied Psychology and Human Development, University of Toronto, Toronto, Canada
15. SAMRC Rural Public Health and Health Transitions Research Unit, School of Public Health, University of the Witwatersrand, Johannesburg, South Africa
16. Non-communicable Diseases Research Unit, South African Medical Research Council, Cape Town, South Africa
17. Division of Exercise Science and Sports Medicine, University of Cape Town, Cape Town, South Africa
18. Chronic Disease Initiative for Africa, University of Cape Town, Cape Town, South Africa
19. Lunenfeld-Tanenbaum Research Institute, Sinai Health System, University of Toronto, Toronto, Canada
20. Sydney Brenner Institute for Molecular Bioscience, University of the Witwatersrand, Johannesburg, South Africa
21. The Centre for Applied Genomics, The Hospital for Sick Children, Toronto, Canada; Program in Genetics and Genome Biology, The Hospital for Sick Children, Toronto, Canada; McLaughlin Centre, University of Toronto, Toronto, Canada
22. Department of Nutritional Sciences, University of Toronto, Toronto, Canada; Joannah and Brian Lawson Centre for Child Nutrition, University of Toronto, Toronto, Canada
23. Department of Paediatrics, University of the Witwatersrand, Johannesburg, South Africa
24. Department of Biochemistry and Biomedical Sciences, McMaster University, Hamilton, Canada
25. Department of Pharmacology and Therapeutics, McGill University, Montreal, Canada
26. Institute for Life Course Health Research, Department of Global Health, Stellenbosch University, Stellenbosch, South Africa
27. Department of Paediatrics, Alberta Children's Hospital Research Institute, Calgary, Canada
28. Department of Physiology, University of Toronto, Toronto, Canada; Departments of Obstetrics and Gynaecology and Medicine, University of Toronto, Toronto, Canada
29. DSI-NRF Centre of Excellence in Human Development, University of the Witwatersrand, Johannesburg, South Africa

For peer review only

30. Division of Molecular Biology and Human Genetics, Stellenbosch University, Cape Town, South Africa
31. School of Nursing and Midwifery, Queens University, Belfast, UK

Corresponding author: Professor Shane Norris; shane.norris@wits.ac.za

Email addresses:
Draper CD: Catherine.Draper@wits.ac.za
Smuts CM: Marius.Smuts@nwu.ac.za
Prioireschi A: Alessandra.Prioreschi@wits.ac.za
Ware L: lisa.ware@wits.ac.za
Dennis CL: cindylee.dennis@utoronto.ca
Awadalla P: Philip.Awadalla@oicr.on.ca
Bassani DG: diego.bassani@sickkids.ca
Bhutta ZA: zulfiqar.bhutta@sickkids.ca
Briollais L: laurent@lunenfeld.ca
Cameron B: BCAMERON@toh.ca
Chirwa T: Tobias.Chirwa@wits.ac.za
Fallon B: barbara.fallon@utoronto.ca
Gray CM: cgray@sun.ac.za
Hamilton J: jill.hamilton@sickkids.ca
Jamison J: janetijamieson@gmail.com
Jaspan H: hbjaspan@gmail.com
Jenkins JM: jenny.jenkins@utoronto.ca
Kahn K: Kathleen.Kahn@wits.ac.za
Kenge AP: Andre.Kengne@mrc.ac.za
Lambert E: vicki.lambert@uct.ac.za
Levitt N: Naomi.Levitt@uct.ac.za
Martin MC: mcmartin@lunenfeld.ca
Ramsay M: Michele.Ramsay@wits.ac.za
Roth, DE: daniel.roth@sickkids.ca
Scherer SW: stephen.scherer@sickkids.ca
Sellen D: dan.sellen@utoronto.ca
Slemming W: Wiedaad.Slemming@wits.ac.za
Sloboda DM: sloboda@mcmaster.ca
Szyf M: moshe.szyf@mcgill.ca
Tollman S: Stephen.Tollman@wits.ac.za
Tomlinson M: markt@sun.ac.za
Tough S: stough@ucalgary.ca
Matthews S: stephen.matthews@utoronto.ca
Richter LM: Linda.Richter@wits.ac.za
Lye S: LYE@lunenfeld.ca

Word count: 4970

For peer review only

Article summary

Introduction: South Africa's evolving burden of disease is challenging due to a persistent infectious disease, burgeoning obesity, most notably among women, and rising rates of non-communicable diseases (NCDs). With two thirds of women presenting at their first antenatal visit either overweight or obese in urban South Africa (SA), the preconception period is an opportunity to optimise health and offset transgenerational risk of both obesity and NCDs.

Methods and analysis: *Bukhali* is the first individual randomised controlled trial in Africa to test the efficacy of a complex continuum of care intervention and forms part of the Healthy Life Trajectories Initiative (HeLTI) consortium implementing harmonised trials in Canada, China, India and SA. Starting preconception and continuing through pregnancy, infancy and childhood, the intervention is designed to improve nutrition, physical and mental health, and health behaviours of South African women to offset obesity-risk (adiposity) in their offspring. Women aged 18-28 years (n=6800) will be recruited from Soweto, an urban-poor area of Johannesburg. The primary outcome is dual-energy x-ray absorptiometry derived fat mass index (fat mass divided by height^2) in the offspring at age 5-years. Community health workers will deliver the intervention randomly to half the cohort by providing health literacy material, dispensing a multi-micronutrient supplement, providing health services and feedback, and facilitating behaviour change support sessions to optimise: (i) nutrition, (ii) physical and mental health, and (iii) lay the foundations for healthier pregnancies and early child development.

Ethics and dissemination: Ethical approval has been obtained from the Human Ethics Research Committee University of the Witwatersrand, Johannesburg, South Africa (M1811111), the University of Toronto, Canada (19-0066-E) and the WHO Ethics Committee (ERC.0003328). Data and biological sample sharing policies are consistent with the governance policy of the HeLTI Consortium (https://helti.org) and South African government legislation (POPIA). The recruitment and research team will obtain informed consent.

Strengths and limitations of the study

- *Bukhali* (HeLTI South Africa) will be the first RCT in Africa to evaluate if a community health worker facilitated intervention starting from preconception through early childhood in those participants that become pregnant will reduce offspring obesity at age 5-years.
- Formative research showed a significant level of maternal trauma, which may impede changes in health-centric behavioural interventions. The *Bukhali* intervention package was therefore modified to include trauma support, and a pragmatic approach of adapting the intervention was adopted.
- Outcomes and most of the trial measures are harmonised with three other HeLTI trial-sites (Canada, China and India) for pooled analyses.
- Formative research indicated a low-level of participation of men (fathers) in the trial, and therefore, a separate and dedicated male research team will target father involvement with alternate approaches and incentives.

For peer review only

Trial registration: this trial is registered with the Pan African Clinical Trials Registry (https://pactr.samrc.ac.za) on 25 March 2019 (identifier: PACTR201903750173871). See Supplementary Table 1

Protocol version: 8 November 2021 (version #3). Any protocol amendments will be communicated to investigators, IRBs, trial participants, and trial registries.

Keywords: pre-conception, South Africa, childhood obesity, early child development, healthy trajectories

For peer review only

Background

Non-communicable diseases (NCDs) such as obesity, hypertension, and diabetes are significant health burdens in many rapidly transitioning middle-income countries[1]. NCDs kill 41 million people each year (71% of deaths) globally, and WHO has identified NCDs as one of the ten leading threats to health[2]. From 1980 to 2014, the prevalence of diabetes changed little in western Europe, but doubled in sub-Saharan Africa (SSA), India and China[1]. SSA countries experience complex health burdens with health systems that are under severe strain. These countries have persisting afflictions from infectious diseases, particularly HIV, TB and malaria, but also now the COVID-19 pandemic. In many respects the impetus to address NCDs as part of the Sustainable Development Goals has stalled as a result of the COVID-19 emergency and a lack of implemented preventive measures.

Recently, a Lancet series highlighted the double burden of malnutrition (DBM) in low- and middle-income countries (LMICs), which is the simultaneous existence within individuals, families and communities of both undernutrition and overweight/obesity[3]. While urban transition is fuelling obesity in LMICs, this is occurring against a background of generations that experienced nutritional deprivation and co-exists with persisting food insecurity, and micronutrient deficiencies. These, directly or in combination with adiposity, may increase susceptibility to cardiovascular disease and diabetes[4]. This broad epidemiological transition in SSA (increasing incidence of cardio-metabolic disease against a backdrop of maternal and child malnutrition, increasing levels of mental health problems, and high-demand on resources needed to address infectious diseases) is manifest to different degrees across the African continent.

A meta-analysis of 61 studies showed that increased preconception and interpregnancy weight gain was associated with gestational diabetes (88% greater risk), preeclampsia (92% greater risk), and large-for-gestational-age (36% greater risk) babies[5]. A systematic review of 38 studies found a consistent association between maternal pre-pregnancy weight and offspring obesity[6]. Furthermore, Heslehurst and colleagues showed that a child has a 2.6-fold greater risk of obesity if the mother is obese prior to conception[7]. A systematic review by Dean and co-authors identified 23 randomised controlled trials of preconception care and counselling delivered via health facilities or community groups, that reported improved women's health behaviours (calorie restriction and increased physical activity), as well as, more positive maternal, neonatal and child health outcomes including an increase in antenatal care seeking and an increased likelihood of breast feeding[8,9]. None of these preconception interventions were conducted in Africa.

In Soweto (urban-poor area of the Johannesburg Metropole covering 200 km^2 with over 1.3 million residents; 6400/km^2) we have found that the prevalence of overweight and obesity in females increases from 10% by age 8 to 43% by age 22 years, while the prevalence in males remains fairly constant into young adulthood at 10%; further, that girls who are obese by age 5 are 42 times more likely to be obese adults[10,11]. Furthermore, 66% of pregnant Soweto women at first antenatal clinic visit, were either overweight or obese, and 10% were diagnosed with gestational diabetes (GDM) in late pregnaancy[12,13]. There is a need for preconception interventions to support women to optimise health and manage weight to combat intergenerational obesity[14]. Specifically, healthier trajectories must be fostered by improving younger women's health prior to, and during pregnancy, and reinforcing good health in the infant to offset obesity and NCD risk.

The Healthy Life Trajectory Initiative (HeLTI), an international consortium, was developed in partnership with the WHO to address childhood obesity. We hypothesise that an integrated complex intervention comprising a continuum of care in 4-phases starting pre-conception and across this period of life (pre-pregnancy, pregnancy, infancy and childhood) will reduce childhood adiposity and the risk for NCDs, as well as, improve child development. Four randomised controlled trials in Ontario and Alberta (Canada)[15], Shanghai (China)[16], Mysore (India)[17], and Soweto (South Africa) have been harmonised to test this hypothesis. This paper describes the HeLTI South African trial protocol in Soweto called *Bukhali* (a Zulu word meaning smart/powerful) signifying the beneficial potential of optimising women's health for herself and her future offspring.

Methods/design

Participant and public involvement

For peer review only - http://bmjopen.bmj.com/site/about/guidelines.xhtml

We constituted a Community Advisory Group with local women, and have involved them in the intervention development and the preparatory phase to enable feedback and input and to facilitate greater acceptance and feasibility of the study. Furthermore, a Participant Advisory Group has been formed as part of a longitudinal qualitative study to further understand and identify preconception health concerns, barriers and solutions, as well as other contextual issues that participants are facing which could influence their participation in the trial. A Stakeholder Advisory Group has been formed with multiple stakeholders (South African government departments of health and education, UNICEF, Save the Children, WHO, NGOs, and academics) to share the development of *Bukhali* and elicit input and guidance around a variety of issues – policy relevance, linkage with current initiatives, ethics, and dissemination.

Formative research and pilot trial study

The trial was designed following the Standard Protocol Items (Recommendations for Interventional Trials (SPIRIT) 2013 statement). Through extensive qualitative (focus groups, in-depth individual interviews, and stakeholder engagement)[18-23] and epidemiological research[24-31], we identified our target group and developed the intervention package. We initially opted for a cluster randomised study design in order to included community peer group sessions as part of the intervention. To assess the feasibility and implementation of this design, a pilot trial of data collection and intervention commenced in 2018 and concluded in 2019. We recruited 1719 participants within six community clusters, randomised the clusters, collected baseline data and biological samples, and implemented the planned intervention and control arm activities. The pilot trial data highlighted critical learning, among others, that: (i) it was feasible to recruit and collect good quality data and biological samples (95% consented to provide a blood sample and 75% consented to provide a DNA sample) from a large group of our target population; (ii) young women living in Soweto lead complicated and economically stressful lives and are pre-occupied with survival strategies, which often entails moving and living at multiple homes across family and friends in the community over time; (iii) there is poor health literacy around preconception health amongst young women in Soweto; (iv) young women were anxious about participation due to fear of criminal activities and abduction; (v) community safety of study community health workers could not be assured; and (vi) we could not effectively deliver the planned community peer group component due to poor attendance despite convenient Saturday meeting times, multiple opportunities during the month to attend, and the provision of refreshments. To address these challenges, we formulated a multi-pronged strategy: (i) we rolled-out a communication campaign; a soap opera series of 11 episodes drawing attention to preconception health issues in multiple languages through two local radio stations with the largest population reach supported by social media engagement (https://soundcloud.com/user-662329775); (ii) we engaged and obtained additional support to operate within the community through community leadership forums and police awareness of the study team; (iii) we produced branded material for staff and vehicles with clear university and project identification to promote credibility; and (iv) we modified the study design (we pivoted from cluster-randomisation to individual-randomisation due to cluster contamination linked to young women shifting between multiple households) and intervention delivery, and jettisoned the peer group component because of poor compliance[32].

Study design

Bukhali is an individual-randomised controlled two-arm trial, where each participant is the unit of randomisation (1:1 ratio to the four-phase preconception-early childhood intervention arm or to standard of care plus control arm). Computer-generated random numbers will be used as the method of generating the allocation sequence. The data team will generate the allocation sequence and this will be implemented through an electronic envelope which is clicked on by the participant with the randomisation officer to reveal their allocation. Researcher and research team collecting trial data will be blinded to participant allocation throughout the trial. All participants will be exposed to the preconception intervention or control programme for up to 18 months. If a participant does not become pregnant within the 18 months, she will have an exit assessment. If a participant becomes pregnant, she will be followed through pregnancy. An 'index child' conceived after randomisation will be followed until age 5-years.

Inclusion/exclusion criteria

Participants will be recruited through a survey of Soweto households (approximately 30000), which is sufficiently large to adequately recruit our target participant number. Women aged 18-28 years are eligible, unless they are: (i) diagnosed with type-I diabetes or epilepsy because these require intensive treatment and management priorities; (ii) present with intellectual disability that hinders informed consent, and (iii), not able or willing to provide consent. Pregnancy at the time of the baseline assessment does not exclude participation. Participants who move residence during the course of the study will continue participation as long as their current address is known, the participant wishes to remain involved, and data can be collected. Participants who have miscarriages will be offered counselling support and remain in the study.

Sample size

The sample size calculation is based on the mean (4.1 kg/m^2) and SD (2.1) for dual-energy x-ray absorptiometry (DXA) derived fat mass index (FMI) calculated from our pre-pubertal data from the Birth to Twenty Plus Cohort in Soweto, South Africa. We will require 765 children to be born in each arm to detect a meaningful difference of 0.25 SD (0.5 kg/m^2) in FMI at age 5-years between the intervention and control groups at 80% power and 5% significance level, and assuming 30% attrition. Therefore, we will recruit approximately 6800 women so reach a target of 1530 pregnancies. The intervention group will receive all four phases of the intervention and the control remains standard of care plus throughout. Pooling of data across the four countries will provide the ability to detect an effect size of 0.1 SD between intervention and control groups at 80% power and 5% significance level. The pooled analysis should also allow us to detect a 0.2 SD difference in our primary outcome in boys and girls separately (although this is dependent on the direction of change being the same across our populations).

Primary and secondary outcome questions:

Primary child outcome: What is the relative effect of a 4-phase intervention on adiposity status at age 5-years as determined by DXA-derived fat mass index (fat mass/height^2) among children of young adult women in the intervention arm, when compared to the children of women who receive standard of care plus (control arm)?

Secondary child outcomes: What are the effects of the 4-phase intervention on child outcomes at age 5-years on:

1. Anthropometry (age- and sex-standardized continuous BMI-z score, BMI growth trajectories, continuous waist circumference z- score, upper arm circumference, head circumference);
2. Cardiometabolic outcomes (systolic and diastolic blood pressure, lipid profile, glucose, insulin, insulin resistance);
3. Behaviour (dietary intake, physical activity, screen time, sedentary behaviour, sleep time);
4. Developmental outcomes (cognitive, motor skills, communication, behavioural); and

Secondary maternal outcomes: What are the effects of the 4-phase intervention on?

1. Anthropometry (overweight and obesity (OWO), DXA-derived body composition, waist circumference);
2. Nutrition (anaemia)
3. Physical health (hypertension);
4. Mental health (depressive symptoms, anxiety) and
5. Behaviour (dietary patterns, physical activity, screen time, sedentary behaviour, sleep time, tobacco and alcohol use);

Phase-specific outcomes

1. Preconception Phase (outcome at conception or exit): What is the effect of the preconception intervention phase on: maternal anthropometry and body composition (overweight and obesity, fat mass index, waist circumference); nutrition (anaemia); health behaviour (physical activity, screen time, sleep); mental health (depressive symptoms, anxiety); and physical health (hypertension)?
2. Pregnancy Phase (parental and child outcomes at birth): What is the effect of the preconception + pregnancy intervention phases on: pregnancy weight gain; rate of gestational diabetes; rate of

For peer review only

gestational hypertension; rate of pre-eclampsia; rate of preterm birth < 37 weeks; rates of large- and small- birthweight for gestational age; and maternal mental health?

3. Infancy Phase (parental and child outcomes across the first two years postpartum): What is the effect of the preconception + pregnancy + infancy intervention phases on: breastfeeding outcomes (initiation, duration, exclusivity, self-efficacy); nutrition (anaemia), health behaviour outcomes (physical activity, screen time, sleep); and mental health outcomes (depressive symptoms, anxiety), and child outcomes (growth, blood pressure, sleep, sedentary and physical activity; feeding practices; developmental outcomes)?
4. Childhood phase (parental and child outcomes 2-5 years postpartum): What is the effect of the preconception + pregnancy + infancy +childhood intervention phases on: maternal nutrition (anaemia), health behaviour outcomes (physical activity, screen time, sleep); and mental health outcomes (depressive symptoms, anxiety); and child outcomes (growth, blood pressure, sleep, sedentary and physical activity; dietary practices; cognitive development outcomes)?

Ethics and regulatory management

Bukhali has ethical approval from the Human Ethics Research Committee of the University of the Witwatersrand, Johannesburg, South Africa (M1811111), the University of Toronto, Canada (19-0066-E) and the WHO Ethics Committee (ERC.0003328). The trial is registered with the Pan African Clinical Trial Registry.

Intervention: community health worker approach

The intervention has been informed by the UK MRC Guidelines for Complex Interventions, and we used the TIDieR (Template for Intervention Description and Replication) Checklist. Using the taxonomy of behaviour change techniques (BCTs)[33], we grounded the behaviour change intervention in the theory of planned behaviour, control theory and social cognitive theory. Figure 1 details the logic model to illustrate the pathways to impact that guided the intervention approach. The intervention is centred on “Living your best life” with four universal objectives that infuse through all phases of the intervention (preconception, pregnancy, infancy and childhood) to: (i) optimise health; (ii) optimise nutrition; (iii) optimise mental health; and (iv) promote early childhood development. The agents of change are modelled upon community health workers, who we call Health Helpers (HHs) to differentiate them from current Department of Health workers. HHs do not need to have any specific qualification or expertise in the area of health promotion, but should ideally have experience in community-based work. The HHs are supported by a supervisor and dietician with access to nurses and a referral network. HHs deliver the intervention through the provision of health literacy resource materials and micronutrient supplements, provide health feedback and services, and they support behaviour change through individual sessions in person or telephonically, and supportive text messaging (see Figure 2). Multiple strategies will be put in place by the HHs (flexibility, reminders, home visits, incentives) to aid in achieving trial compliance.

Healthy Conversation Skills and trauma-informed care: HHs will be trained in Healthy Conversation Skills (HCS), a technique specifically developed and tested for use with socioeconomically disadvantaged participants to improve self-efficacy and support behaviour change. HCS is based on a social-cognitive model of health behaviour, which emphasises the role of increasing self-efficacy in promoting behaviour change. It recognises that knowledge alone is insufficient to change behaviour unless people are also motivated and empowered to change. Trained in HCS, HHs will explore the barriers that that women experience in trying to change their behaviour, ways of over-coming these barriers and setting goals for change. The HCS approach enables a woman to reflect on her particular priorities for physical and mental health, and to support her in finding her own solutions to challenges she experiences. The training is standardised and supported by the MRC Lifecourse Epidemiology Unit (UK). HCS training is aimed at achieving four basic competencies that are known to be useful in supporting behaviour change: (i) the use of open-discovery questions, questions that begin with ‘how’ and ‘what’ and lead people to explore and find their own ways of overcoming barriers to change; (ii) listening more than talking which allows people to identify and take control of their own behaviour change; (iii) reflecting on practice in order to be more effective; and (iv) supporting the use of SMARTER goal setting so that participants have a sense of change and progress[34-36]. To the HCS approach we

For peer review only

have added selected skills from the WHO Problem Management Plus (individual psychological help for adults impaired by distress in communities exposed to adversity).

Health literacy resource material: With topic experts and a specialised curriculum developer, we integrated behaviour change theory and designed both HH facilitator manuals and participant resource materials. The resource material encompasses 9 modules (women's health, chronic diseases, diet, sleep, physical activity and fitness, sitting time, body size and image, emotional awareness, and caregiving) in 6 chapters, and these modules repeat with new content every 6 chapters. The resource books contain essential educational content, local narratives to convey key concepts, and space for participants to engage with the material through the use of checklists, diaries and reflections. The approach for each module is that of: (i) Knowing (what do I know about it?); (ii) Doing (what am I doing about it?); and (iii) Becoming (how do I live my best life?). Three preconception health resource books are provided; one book is provided for pregnancy health, and supplementary resource materials to the South African Road to Health Booklet are provided for early child development.

Multi-micronutrient supplementation: based on 6-monthy point-of-care haemoglobin concentration assessments, the HHs will dispense and monitor micronutrient supplement use. The MMN supplement is based on WHO recommendations (Table 1) to optimise nutrition and address anaemia, based on haemoglobin concentrations:

- Non-anaemic women (Hb ≥12 g/dL at enrolment): Provision of a micronutrient supplement containing approximately 27 mg iron twice (2x) per week.
- Mildly anaemic women (Hb <12 g/dL and >7g/dL at enrolment): Provision of a micronutrient supplement daily for six months.
- Severely anaemic women (Hb <7g/dL at baseline): Receive treatment regime according to current standard of care in South Africa.

Supplement use will continue throughout the preconception period until pregnancy or exit. If the participant becomes pregnant, the supplement regimen will then shift to daily use throughout pregnancy and 6-months post-partum.

Table 1: Nutrient composition of multiple micronutrient supplements

Micronutrient	Unit	Nutrient Reference Value (NRV) EU
Vitamin A	2664 IU	100% (800 µg / 2664 IU)
Vitamin D3	200 IU	100% (5 µg / 200 IU)
Tocopherol (Vitamin E)	15 mg	125% (12 mg / 17.9 I
Vitamin K1	55 µg	73% (75 µg)
Thiamine	1.4 mg	127% (1.1 mg)
Riboflavin	1.4 mg	100% (1.4 mg)
Niacinamide	18 mg	113% (16 mg)
Pantothenic acid	6 mg	100% (6 mg)
Pyridoxine (Vitamin B6)	1.9 mg	136% (1.4 mg)
Biotin	30 µg	60% (50 µg)
Folic acid	600 µg	300% (200 µg)
Cyanocobalamin (Vitamin B12)	2.6 µg	100% (2.5 µg)
Ascorbic acid	60 mg	75% (80mg)
Copper	1.15 mg	100% (1 mg)
Iodine	250 µg	167% (150 µg)
Iron	27 mg	193% (14 mg)

For peer review only

Selenium	50 µg	100% (55 µg)
Zinc	10 mg	100% (10 mg)

Services: Through individual in-person or telephonic sessions, HHs will provide: (i) specific health feedback to participants based on their baseline results (BMI (obesity), blood pressure (hypertension), haemoglobin concentration (anaemia), mental health, and lifestyle (physical activity, sedentary behaviour, sleep); (ii) offer home-based HIV and pregnancy testing with counselling, utilise HCS to identify aspects of health behaviour change, set goals and monitor, and provide access to healthcare referral networks. For overweight and obese women, additional waist circumference measurements will be provided. During the infant and child periods, HHs continue the monthly consultations and support women with aid of the South African Road to Health Booklet (https://sidebyside.co.za/resources/road-to-health-book/) and postnatal clinic visits.

Control arm: call centre approach (standard of care plus)
In addition to access to standard of care offered through the public health system, control arm participants will have a dedicated call centre. The rationale to add a standard of care plus programme is to maintain contact with participants, and minimise attrition bias. Similar to the intervention team, the call centre team will receive training in the delivery of the modules. To partially control for special attention given to women in the intervention arm, the call centre will contact participants once a month and deliver a curriculum on "life skills" via telephone, SMS and email. The non-health specific intervention material will be standardised (see Figure 2) and the modules of the curriculum will include: (i) civic rights and responsibilities (How do I apply for an identity document; what does it mean to vote?); (ii) financial education (savings & budgets; how do I open a bank account); (iii) job readiness (preparing a CV; job interview skills; volunteering); (iv) navigating social media (internet, different social media; how social media can be harmful), and (v) social grants. HIV and pregnancy testing services will be offered on request for free at the research centre.

Process evaluation
For all intervention (health worker-centric) and control (call centre-centric) components, Standard Operating Procedures (SOPs) support the HHs and call centre team to deliver their respective modules in a structured and uniform way. We will follow the MRC guidance on process evaluation of complex interventions[37]. Evaluation will focus on implementation, mechanisms of impact and context. Assessment of implementation will focus on finding out what is delivered and how delivery is achieved, and which is vital information if the intervention is to be replicated. We will assess compliance by counts using a log (for example, contacts made), and by discussions between HHs and participants. We will measure fidelity of intervention delivery by observing HHs during one-to-one contacts with intervention group participants, focusing on HHs use of HCS and the competencies they demonstrate. We will also observe the contact between call centre assistants and control participants to record the nature of interactions. We will monitor intervention dose by recording the frequency and duration of contact between participants and HHs using diaries. To understand the mechanisms through which the intervention brings about change in the trial's specified outcomes, we will assess the acceptability of the intervention, and women's experiences of contact with HHs using interviews and focus groups. In assessing the context, we will aim to identify factors that might act as barriers or facilitators to intervention implementation or effects. This will include local and national policy, local service configuration and provision, and socio-demographic and environmental factors within the communities taking part in the study. Qualitative methods (interviews and focus groups), including longitudinal approaches, will be used to further explore specific issues of relevance, in terms of participants' experiences of the trial, key issues facing participants, and factors influencing implementation of the trial.

Data collection, management and analysis
Table 2 and 3 details the phenotype measures and biological sample collection on women, biological fathers and index child. Formative research has indicated low-level (10%) biological father participation in the study. A dedicated research team will be established increase father participation through alternate approaches (for example: data collection flexibility in evening and on Saturdays; encouraging fathers to accompany pregnant women for their ultrasound scan). There is substantial harmonisation of data, samples and quality management across the four HeLTI studies. For

For peer review only

example, over 1800 core variables have been harmonized from preconception through to infancy (6 months). All data will be managed through real-time capturing, reporting and quality control utilising REDCap electronic data capture tools[38]. Maelstrom Research at McGill University (Canada), has been supporting the harmonisation process across the sites and developing the online catalogue and metadata, which are available on their website (https://www.maelstrom-research.org/study/helti-hp). In addition, Maelstrom will support the implementation of a data infrastructure within each country using DataSHIELD/Opal software[39] to allow federated data analysis (country-specific or meta-analysis by pooling estimates across countries) to be conducted without having access to individual participant data or exporting and pooling data on a central server. This capacity will provide a mechanism for addressing restrictive regulation regarding exporting data across countries. The data management system coupled with our laboratory management system of samples (different tissues at different time points) will enable researchers to conduct future mechanistic studies and participants have provided consent for this on provision of IRB approval for such studies. All personal data are managed in a separate and secure with restricted access in line with the Protection of Personal Information Act of South Africa.

Analysis plan

Standard data screening/cleaning procedures will be conducted including the extent and pattern of missing data. When data are missing at random (MAR), which we expect to be the case, we will utilise multiple imputation so as to maximise sample size and study power in a dataset. We will assess the comparability at randomisation of the groups using descriptive analyses. We will also assess process indicators including recruitment and retention, and indicators of programme delivery in the Intervention Group. Sensitivity analyses will be performed to assess the influence of attrition and compliance. Intention-to-treat analysis will be used for key outcomes based on all viable pregnancies adjusted for compliance and selection of viable pregnancies to minimise bias. For the primary outcome, we will assess the effect of the intervention on fat mass index at age 5-years. The concept of this multiple-phase trial, is that the outcomes of interest in a particular phase result from the intervention administered in the same phase, but is also mediated by the outcomes observed in the previous phase(s). For example, a reduction in obesity risks during pregnancy caused by the intervention in this phase will impact birth weight and the child weight/BMI observed in the next phase. Therefore, at the end of the study, we will measure an “overall” effect of the interventions over the four phases on the final outcomes, but also, could assess how the association between the final outcomes and the interventions is mediated by the phase-specific outcomes (see Figure 3).

Other secondary anthropometric outcomes to be assessed at 5-years of age include OWO, BMI and centiles, and adiposity (skin-fold thickness, arm circumference). We will also assess the effect of the intervention on glucose metabolism, blood pressure and cognitive outcomes. Mixed models with random intercept/slope effects and fixed effects (for the intervention) will be used to estimate the intervention effect. Due to possible heterogeneity of effects for key outcomes according to the time of initiation of the intervention (e.g. preconception versus pregnancy) we will conduct a subgroup analysis stratifying by this variable, testing for heterogeneity. The distribution of modifiable risk factors for childhood obesity, poor cardiometabolic and neurodevelopmental outcomes at baseline, and their changes over time will be assessed according to study group.

For peer review only

Table 2. Longitudinal data collection in *Bukhali* (four-phases of the trial; M-mother, F-Father, C-Infant/child)

Domain	Measure	Preconception		Pregnancy		Postnatal				
**Time point (months)**		**Baseline**	**18 mo (exit)**	**10-17 wk**	**24-28 wk**	**Delivery**	**6 mo**	**12 mo**	**24 mo**	**60 mo**
**Demography**	Demographic and socio-economic data, household composition, occupation, education, living environment	M	M	M	F		M	M	M	M
	Date of birth / sex	M			F	C				
**Anthropometry and body composition**	Height/length	M			F	C	C	C	C	C
	Weight	M	M	M	M/F	C	M/C	M/C	M/C	M/C
	Gestational weight gain			M	M					
	Body Mass Index	M	M	M	M/F		M	M	M	M
	Adiposity: DXA (Adult)	M	M		F		M		M/C	M/C
	Skinfolds (triceps & subscapular) (*if scan is not possible)			M	M		M*		M*	M*
	Peapod body composition					C	C			
	Waist circumference	M	M		F		C	M/C	M/C	M/C
	Fetal anthropometry (gestational age and fetal growth)			C	C	C (GA)				
	Mid upper arm circumference	M	M	M	M/F	C	C	C	M/C	M/C
	Head circumference					C	C	C	C	C
**Clinical**	Pregnancy Complications			M	M					
	Birth complications, survival					M/C	C	C	C	C
	APGAR score 1 & 5 mins					C				
	HIV test			M	F					M
	Pregnancy test (pre-DXA scan)	M	M				M		M	M
	Blood pressure	M	M	M	M/F		M	M	M/C	M/C
	Anaemia (HemoCue)	M	M	M	M/F			M	M	M
	HbA1c	M	M	M	M/F			M	M	M
	Random blood glucose	M	M	M	F					
	Fasting blood glucose				M			M	M/C	M/C
	Oral glucose tolerance test				M					M/C
	Plasma & Serum collection	M	M	M	M/F			M	M/C	M/C
	DNA collection	M	M	M	M/F			M		M/C
	Urine spot sample	M	M		M/F				C	M/C
	Urine dipstick	M	M	M	M/F				C	M/C
	Heel-prick blood								C	
	Buccal swab								C	

**Lifestyle and health behaviour**	Diet intake (Dietary diversity & Household Food Insecurity Access Scale)	M	M	M	M/F			M	M	M
	Breastfeeding Questionnaire					M				
	Feeding (breastfeeding, solid food introduction, diversity, eating behaviour; SUNRISE Questionnaire)						C	C	C	C
	Physical activity (M:GPAQ & Sitting Time Quesstionnaire)	M	M	M	M/F			C/M	**M**	M
	Step Test		M		F					
	Accelerometry (sub-study)								C	C
	Screen Time Questionnaire	M	M	M	M/F		C	C	C	C
	Sleep: Pittsburgh Sleep Quality Index	M	M	M	M/F		M	M	M	M
	Brief Infant Sleep Questionnaire						C	C	C	
	SUNRISE sleep questionnaire									C
	Tobacco, alcohol, and drug use: Exposure questionnaire (WHO-STEPS, AUDIT-Questionnaire)	M	M	M	M/F			M	M	M
	Environmental tobacco exposure				M			C	C	C
	Child Care Questionnaire						M	M	M	M
**Mental and physical health, and chid development**	Depression (PHQ9)	M	M	M	M/F			M		M
	Depression (Edinburgh)			M	M		M			
	Stress (PSS)			M	M/F		M		M	M
	Stressful life events (ACEs)	M			F					
	Generalised Self-Efficacy Scale	M	M	M	M/F			M		M
	Social Support Questionnaire	M	M	M	M/F					
	Social Provisions Scale				M					M
	Medical history (and TB), family history, drug, supplement history	M	M	M	M/F		M	M	M	M
	Breasfeeding efficacy				M					
	Anxiety (GAD-7)	M	M	M	M/F			M	M	M
	Medication/supplement use	M	M	M	M/F	M/C	M/C	M/C	M/C	M/C
	Medical events (hospitalisation)				M	M/C	M/C	M/C	M/C	M/C
	Births, pregnancy & reproductive health	M		M		M	M	M	M	M
	Vaccinations						C	C	C	C
	Emotional health Questionnaire	M	M	M	M/F					
	Ages and Stages questionnaire						C	C	C	C
	Parenting practices (Brigance Parent-Child Interaction Scale)							M	M	

	Neurodevelopment (WHO Global Scale for Early Development - short-form)							C	C	
	Executive function (Early Years Toolbox)									C
	Temperament/behaviour (Strengths and Difficulties Questionnaire)								C	C
	School readiness (International Development and Early Learning Assessment)									C
	Stimulation in the home (UNICEF Multiple Inidcators Cluster Survey Early Childhood Development, Home Learning Environment)								C	C

Governance

The shared governance and harmonisation structures for the HeLTI trials include: Research Committee of country Principal Investigators; a single Data Monitoring Committee that is independent from the sponsor and competing interests (DMC Charter is available on the HeLTI website); WHO Monitoring and Evaluation Team; and a HeLTI Secretariat. The trial is blinded to the investigators and research data collection team, but the data management team will unblind participant's allocation upon request by the DMC or ethics committee for key reporting or in cases of risk. A governance framework has been developed and adopted by all partners that details the management and access of data and biological samples. The HeLTI website (https://helti-net.org) provides more information including contact details of the HeLTI office and secretariat and information on the Data Access Portal through which investigators from around the world can view and request access to data/biospecimens from individual HeLTI country datasets. HeLTI will also be registered as a hub on Global Health Network (https://tghn.org) The Research Committee is supported by seven cross-consortium active working groups, including: (i) Core Variable Harmonization, (ii) Interventions and Retention, (iii) Early Child Development Measures, (iv) Biospecimen Collection and Management, (v) Data Management and Quality Assurance, (vi) Capacity Building, and (vii) Publications. Results will actively be disseminated within the four countries and through WHO mechanisms.

Monitoring

An independent clinical research associate will audit the trial annually. WHO reviews quality assurance data of the trial quarterly. An electronic management system is in place for trial HHs and staff to record and manage adverse events. Utilising REDCap, Adverse Event Forms will be completed online, which will trigger a process of alerts and recommendations from the investigators, researchers and project coordinators to best handle the event. All events are also discussed at the HH weekly debrief session and reported to the DMC.

For peer review only

Table 3. Longitudinal biological sample collection in *Bukhali* (four-phases of the trial)

	Preconception		Pregnancy		Postnatal		
	**Baseline**	**18 mo**	**10-17 wk**	**24-28 wk**	**12 mo**	**24 mo**	**60 mo**
**Mother**							
Blood collection	X (random)	X (random)	X (random)	X (fasting; OGTT)	X (fasting)	X (fasting)	X (fasting; OGTT)
Plasma	X	X	X	X	X	X	X
Serum	X	X	X	X	X	X	X
DNA	X		X	X	X		X
Urine (spot sample)	X	X		X			X
Urine (dipstick)	X	X	X	X			X
**Father**							
Blood - random				X			
Plasma				X			
Serum				X			
DNA				X			
Urine (spot sample)				X			
Urine (dipstick)				X			
**Infant/Child**							
Blood collection (DBS)					X		
Blood collection						X (fasting)	X (Fasting; OGTT)
Plasma						X	X
Serum						X	X
DNA							X
Buccal swab						X	
Urine (spot sample & dipstick)						X	X

Trial status and impact of the Covid-19 pandemic

Trial formative research started in April 2017, the pilot trial was initiated in 2018, main trial recruitment began in October 2019 and is expected to conclude early in 2022, and the trial is expected to be completed by 2029. The collective HeLTI initiative provides an unprecedented preconception health platform with the potential to expand to other settings. To maximise the learning and impact of these studies, HeLTI aims to host postgraduate students and training opportunities, and facilitate accelerated managed access to data and biological samples. Our formative work has revealed just how complex both the environments and lives of young urban women in South Africa are. The health implications are further compounded by poorer than anticipated health literacy levels. Among these women, health is not prioritised amidst competing economic and food security concerns, and the daily stress of navigating challenging household (relationships, households with multi-families) and community circumstances (poverty, crime and exposure to violence). Also, the Soweto environment is becoming more obesogenic with easier access to less healthy fast foods that are affordable and sold on most street corners. This makes the decision to purchase healthier, more expensive foods that are sold further away from the home less appealing[20]. For these reasons, we have opted for a more pragmatic and adaptive trial design, whereby the intervention package can be modified to the circumstances of each participant and can incorporate new learning on how best to engage, support and improve compliance. The strength of this approach is to remain relevant and responsive to the urban poor context, and thereby, increase the likelihood of achieving health behaviour change. The challenge of this adaptive and individually-

For peer review only

responsive approach is how to capture and analyse the dose effect of intervention elements. To address this challenge, we have setup a sophisticated data management and dashboard system which can monitor key quality assurance, delivery and compliance indicators that are not only useful for trial management, but also assist the intervention and control arm teams.

Six months into the main trial the Covid-19 pandemic hit South Africa through a series of waves and lockdown levels. A hard lockdown (level 5) was implemented in South Africa from 26 March - 30 April 2020. Thereafter a level 4 lockdown was implemented until 31 May 2020. Level 3 then lasted until 17 August, when the country moved to level 2 and finally level 1 (September 2021). The recruitment has been severely interrupted particularly between 26 March and 30 July 2020. Upon resuming community recruitment, additional Covid-19 specific questions were added to the questionnaire, support and surveillance protocols were implemented and a dedicated COVID-19 nurse was appointed. Intervention face-to-face interactions were replaced with virtual face-to-face sessions during more severe lockdowns and micronutrient supplements were delivered to participants' homes. To boost trial recruitment, we launched a revised community drive that included community pop-up stands, and enlisted community leaders aiding recruitment drives. We have also produced a fidelity protocol and participant retention standard operating procedures. In conclusion, changing the course of the obesity tide globally will require a major concerted effort to examine initiatives across the lifecourse. The preconception period, in particular, represents an exciting and largely untested stage in life to prevent the transgenerational impacts of maternal metabolic and mental health.

Acknowledgements
The authors extend our gratitude to the South African Medical Research Council and the Canadian Institutes of Health Research for funding *Bukhali*. We greatly appreciate the willingness of all women who have agreed to participate in the study and the staff who are implementing this ambitious research programme. We also acknowledge the role of WHO in supporting the HeLTI collaboration and providing quality assurance support to *Bukhali.* We thank the HeLTI PI Research Committee and HeLTI Office for their intellectual contributions, comradery and administrative support.

Author Contributions
SAN and SL are co-principal investigators for Healthy Life Trajectory Initiative South Africa. SAN, CD, LW, AP and SL wrote the initial protocol draft. CMS, CLD, PA, DGB, ZAB, LB, BC, TC, BF, CG, JH, JJ, HJ, JMJ, KK, AK, EL, NL, MCM, MR, DER, SWS, DS, WS, DMS, MS, ST, MT, ST, SM, and LMR read and contributed to the final version. All authors provided edits and critiqued the manuscript for intellectual content.

Funding
The authors extend our gratitude to the South African Medical Research Council (Strategic Health Innovation Partnerships grant) and the Canadian Institutes of Health Research for funding (grant number: HLS 151553) *Bukhali.*

Trial Sponsor
University of the Witwatersrand, Johannesburg, South Africa; Contact Dr Drennan (robin.drennan@wits.ac.za).

Competing interests
We have no conflicts of interest to declare. The HeLTI Council of funders and WHO require all Principal Investigators to complete annual Declarations of Interest and to commit to having no direct or indirect relations with the tobacco, arms and food industries during the course of the trial.

Patient consent for publication Not required.

Provenance and peer review Not commissioned; peer-reviewed for ethical and funding approval prior to submission.

References

1. Wang H, Naghavi M, Allen C, *et al*. Global, regional, and national life expectancy, all-cause mortality, and cause-specific mortality for 249 causes of death, 1980-2015: a systematic analysis for the global burden of disease study 2015. *Lancet* 2016;388:1459–544.
2. World Health Organisation. https://www.who.int/news-room/spotlight/10-global-health-issues-to-track-in-2021 Accessed November 2021
3. Popkin BM, Corvalan C, Grummer-Strawn LM. Dynamics of the double burden of malnutrition and the changing nutrition reality. *Lancet* 2020; 395:65-74.
4. Wells JC, Sawaya AL, Wibaek R, *et al*. The double burden of malnutrition: aetiological pathways and consequences for health. *Lancet* 2020;395:75-88.
5. Nagpal TS, Souza SCS, Moffat M, *et al*. Does prepregnancy weight change have an effect on subsequent pregnancy health outcomes? A systematic review and meta-analysis. *Obes Rev* 2021; e13324.
6. Woo Baidal JA, Locks LM, Cheng ER, *et al*. Risk factors for childhood obesity in the first 1,000 days: a systematic review. *Am J Prev Med* 2016;50:761–79
7. Heslehurst N, Vieira R, Akhter Z, *et al*. The association between maternal body mass index and child obesity: A systematic review and meta-analysis. *PLoS Med* 2019;16:e1002817.
8. Dean SV, Lassi ZS, Imam AM, *et al*. Preconception care: nutritional risks and interventions. *Reprod Health* 2014;11:S3.
9. Dean SV, Lassi ZS, Imam AM, *et al*. Preconception care: closing the gap in the continuum of care to accelerate improvements in maternal, newborn and child health. *Reprod Health* 2014;11:S1
10. Nyati LH, Pettifor JM, Norris SA. The prevalence of malnutrition and growth percentiles for urban South African children. *BMC Public Health* 2019;19:492.
11. Lundeen EA, Norris SA, Adair LS, *et al*. Sex differences in obesity incidence: 20-year prospective cohort in South Africa. *Pediatr Obes*. 2016;11:75-80.
12. Macaulay S, Ngobeni M, Dunger DB, Norris SA. The prevalence of gestational diabetes mellitus amongst black South African women is a public health concern. *Diabetes Res Clin Pract*. 2018;139:278-287
13. Prioreschi A, Wrottesley SV, Said-Mohamed R, *et al*. Understanding how maternal social and biological factors are related to fetal growth in an urban South African cohort. *J Dev Orig Health Dis*. 2021;12:79-87.
14. Barker M, Dombrowski SU, Colbourn T, *et al*. Intervention strategies to improve nutrition and health behaviours before conception. *Lancet*. 2018; 391:1853-1864.
15. Dennis CL, Marini F, Dick JA, *et al*. Protocol for a randomised trial evaluating a preconception-early childhood telephone-based intervention with tailored e-health resources for women and their partners to optimise growth and development among children in Canada: a Healthy Life Trajectory Initiative (HeLTI Canada). *BMJ Open*. 2021;11:e046311.
16. Wu Y, Marc I, Bouchard L, *et al*. Study protocol for the Sino-Canadian Healthy Life Trajectories Initiative (SCHeLTI): a multicentre, cluster-randomised, parallel-group, superiority trial of a multifaceted community-family-mother-child intervention to prevent childhood overweight and obesity. *BMJ Open*. 2021;11:e045192.
17. Kumaran K, Krishnaveni GV, Suryanarayana KG, *et al*. Protocol for a cluster randomised trial evaluating a multifaceted intervention starting preconceptionally-Early Interventions to Support Trajectories for Healthy Life in India (EINSTEIN): a Healthy Life Trajectories Initiative (HeLTI) Study. *BMJ Open*. 2021;11:e045862.
18. Cohen et al. Material and relational difficulties: The impact of the household environment on the emotional wellbeing of young black women living in Soweto, South Africa. . *Family Issues* 2020, Vol. 41(8) 1307–1332
19. Draper CE, Bosire E, Prioreschi A, *et al*. Urban young women's preferences for intervention strategies to promote physical and mental health preconception: A Healthy Life Trajectories Initiative (HeLTI). *Prev Med Rep* 2019;14:100846
20. Ware LJ, Prioreschi A, Bosire E, *et al*. Environmental, Social, and Structural Constraints for Health Behavior: Perceptions of Young Urban Black Women During the Preconception

Period – A Healthy Life Trajectories Initiative. *J Nutr Educ Behav*. 2019; S1499-4046:30178-2
21. Bosire EN et al. Young women's perceptions of life in urban South Africa: Contextualising the preconception knowledge gap. African *J Reproduc Health*. 2021;25:39-49.
22. Prioreschi A, Wrottesley SV, Slemming W, *et al*. A qualitative study reporting maternal perceptions of the importance of play for healthy growth and development in the first two years of life. *BMC Pediatr* 2020 Sep 9;20:428
23. Wrottesley SV, Prioreschi A, Slemming W, *et al*. Maternal perspectives on infant feeding practices in Soweto, South Africa. Public Health Nutr. 2021; 24:3602-3614
24. Silubonde TM, Baumgartner J, Ware LJ, *et al*. Adjusting Haemoglobin Values for Altitude Maximizes Combined Sensitivity and Specificity to Detect Iron Deficiency among Women of Reproductive Age in Johannesburg, South Africa. *Nutrients*. 2020;12:633
25. Ware LJ, Kim AW, Prioreschi A, Nyati LH, Taljaard W, Draper CE, Lye SJ, Norris SA. Social vulnerability, parity and food insecurity in urban South African young women: the healthy life trajectories initiative (HeLTI) study. *J Public Health Policy*. 2021;42:373-389.
26. Prioreschi A, Wrottesley SV, Norris SA. Physical Activity Levels, Food Insecurity and Dietary Behaviours in Women from Soweto, South Africa. J Community Health. 2020 Jun 19
27. Kehoe S, Wrottesley S, Prioreschi A, *et al*. Food insecurity, diet quality and body composition: data from the Healthy Life Trajectories Initiative pilot survey in urban Soweto, South Africa. *Public Health Nutr*. 2021;24:1629-1637
28. Wrottesley SV, Prioreschi A, Kehoe SH, Ward KA, Norris SA. A maternal "mixed, high sugar" dietary pattern is associated with fetal growth. *Matern Child Nutr* 2019;16: e12912
29. Prioreschi A, Wrottesley SV, Said-Mohamed R, *et al*. Understanding how maternal social and biological factors are related to fetal growth in an urban South African cohort. *J Dev Orig Health Dis*. 2020 Feb 17:1-9
30. Wrottesley SV, Prioreschi A, Norris SA. The association between fetal growth and neonatal adiposity in urban South African infants. *Pediatr Obes*. 2021;16:e12737
31. Soepnel L et al. Obesity and adiposity of 3- to 6-year-old children born to mothers with hyperglycemia detected in pregnancy in an urban South African setting. Annals Human Biol. 2020 48:81-92
32. Draper CE, Prioreschi A, Ware LJ, *et al*. Pilot implementation of *Bukhali*: A preconception health trial in South Africa. *SAGE Open Med*. 2020 Jul 7;8
33. Michie S, Richardson M, Johnston M, *et al*. The behavior change technique taxonomy (v1) of 93 hierarchically clustered techniques: building an international consensus for the reporting of behavior change interventions. *Ann Behav Med* 2013;46:81-95.
34. Lawrence W, Black C, Tinati T, *et al*. 'Making every contact count': Evaluation of the impact of an intervention to train health and social care practitioners in skills to support health behaviour change. *J Health Psychol*. 2016;21:138-51.
35. Lawrence W, Vogel C, Strömmer S, *et al*. How can we best use opportunities provided by routine maternity care to engage women in improving their diets and health? *Matern Child Nutr* 2020;16:e12900.
36. Adam LM, Jarman M, Barker M *et al*. Use of healthy conversation skills to promote healthy diets, physical activity and gestational weight gain: Results from a pilot randomised controlled trial. *Patient Educ Couns* 2020;103:1134-1142.
37. MRC guidance on process evaluation of complex interventions, (https://www.mrc.ac.uk/documents/pdf/mrc-phsrn-process-evaluation-guidance-final/).
38. PA Harris, R Taylor, R Thielke, *et al*, Research electronic data capture (REDCap) – A metadata-driven methodology and workflow process for providing translational research informatics support, *J Biomed Inform 2009;42:377-81*
39. Gaye A, Marcon Y, Isaeva J, *et al*. DataSHIELD: taking the analysis to the data, not the data to the analysis, *Int J Epi* 2014; 43:1929–1944

Legends

Figure 1. Pathways to impact model that informed the intervention development

Figure 2. Intervention and control arm components and dosage

Figure 3. Impact of outcomes in a multi-phase trial

For peer review only

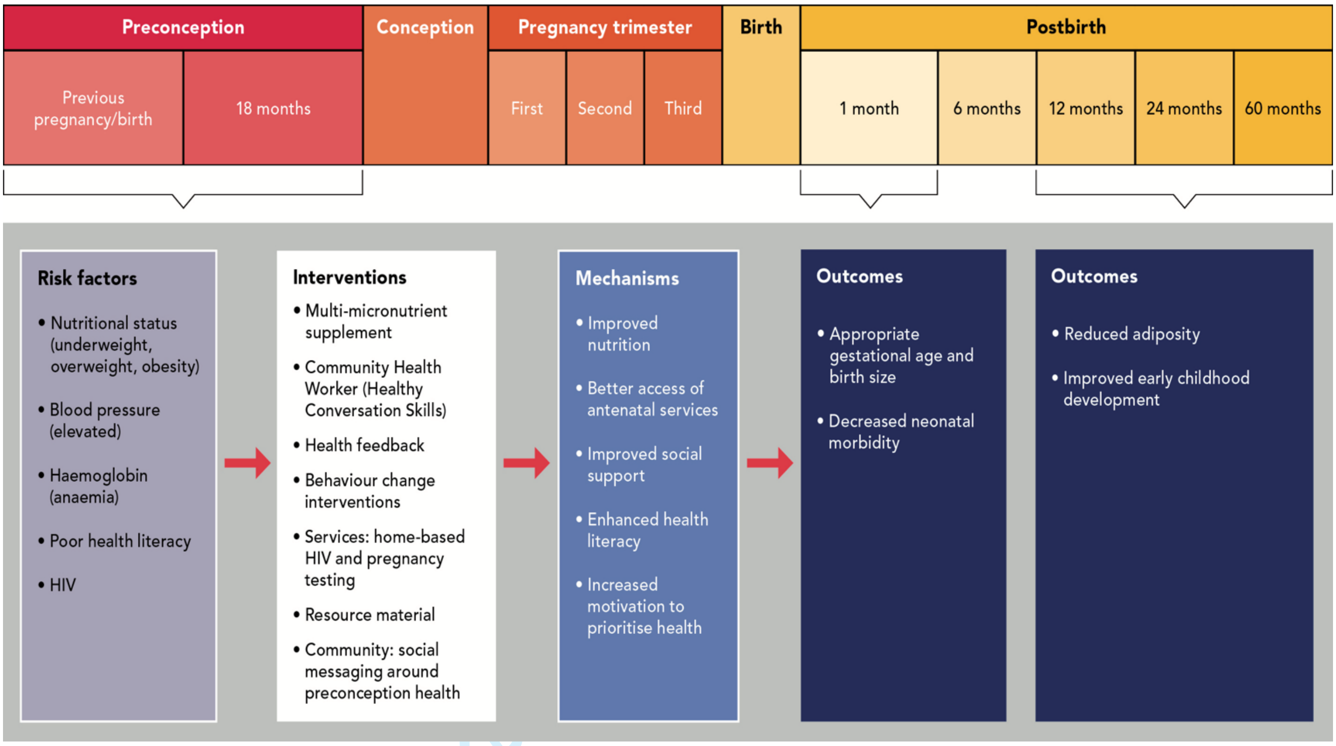
Preconception
Previous pregnancy/birth
18 months
Conception
Pregnancy trimester
First
Second
Third
Birth
Postbirth
1 month
6 months
12 months
24 months
60 months
Risk factors
• Nutritional status (underweight, overweight, obesity)
• Blood pressure (elevated)
• Haemoglobin (anaemia)
• Poor health literacy
• HIV
Interventions
• Multi-micronutrient supplement
• Community Health Worker (Healthy Conversation Skills)
• Health feedback
• Behaviour change interventions
• Services: home-based HIV and pregnancy testing
• Resource material
• Community: social messaging around preconception health
Mechanisms
• Improved nutrition
• Better access of antenatal services
• Improved social support
• Enhanced health literacy
• Increased motivation to prioritise health
Outcomes
• Appropriate gestational age and birth size
• Decreased neonatal morbidity
Outcomes
• Reduced adiposity
• Improved early childhood development

Intervention

	Preconception 18 months	Pregnancy 9 months	Early childhood 60 months
Health literacy resources	3	2	1
Multi-micronutrient supplement	18	5	6
In-person session & health check	3	2	10
Telephonic contacts	9	3	20
Dietician (at least 1 visit for at risk participants)	1	1	1
MINIMUM TOTAL DOSE	33	12	37

Control

	Preconception 18 months	Pregnancy 9 months	Early childhood 60 months
In-person session & services offered	3	1	10
Telephonic contacts	9	3	20
MINIMUM TOTAL DOSE	12	4	30

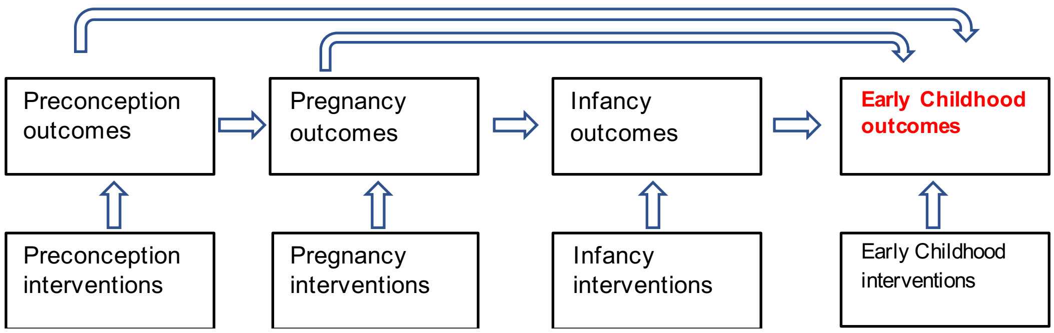

For peer review only

Table 1: World Health Organization Trial Registration Data Set

Data category	Information
Primary registry and trial identifying number	PACTR201903750173871
Date of registration in primary registry	25 March 2019
Source(s) of monetary or material support	South African Medical Research Council; Canadian Institutes of Health Research
Primary sponsor	University of the Witwatersrand, Johannesburg, South Africa
Contact for public queries	SAN; shane.norris@wits.ac.za
Contact for scientific queries	SAN; shane.norris@wits.ac.za
Public title	HeLTI-SA: a preconception randomised control trial
Countries of recruitment	South Africa
Health condition(s) or problem(s) studied	Child obesity and non-communicable disease risk
Intervention(s)	Active comparator: community health worker, health literacy, behaviour change, dietetic and counselling support; risk-screening health services; multi-micronutrient supplement<br>Placebo comparator: standard of care plus non-health related call centre support
Key inclusion and exclusion criteria	Women aged 18-28 years are eligible, unless they are: (i) diagnosed with type-I diabetes or epilepsy because these require intensive treatment and management priorities; (ii) present with intellectual disability that hinders informed consent, and (iii), not able or willing to provide consent.
Study type	Complex prevention intervention; individual randomisation; research team blind to randomisation; phase II
Date of first enrolment	October 2019
Target sample size	6800 women
Recruitment status	Recruiting
Primary outcome(s)	DXA-derived fat mass index (fat mass/height^2)
Key secondary outcomes	Maternal and child anthropometry, cardiometabolic, and behaviour, and child developmental outcomes

For peer review only

Reporting checklist for protocol of a clinical trial.

Based on the SPIRIT guidelines.

Instructions to authors

Complete this checklist by entering the page numbers from your manuscript where readers will find each of the items listed below.

Your article may not currently address all the items on the checklist. Please modify your text to include the missing information. If you are certain that an item does not apply, please write "n/a" and provide a short explanation.

Upload your completed checklist as an extra file when you submit to a journal.

In your methods section, say that you used the SPIRITreporting guidelines, and cite them as:

Chan A-W, Tetzlaff JM, Gøtzsche PC, Altman DG, Mann H, Berlin J, Dickersin K, Hróbjartsson A, Schulz KF, Parulekar WR, Krleža-Jerić K, Laupacis A, Moher D. SPIRIT 2013 Explanation and Elaboration: Guidance for protocols of clinical trials. BMJ. 2013;346:e7586

		Reporting Item	Page Number
**Administrative information**			
Title	#1	Descriptive title identifying the study design, population, interventions, and, if applicable, trial acronym	1
Trial registration	#2a	Trial identifier and registry name. If not yet registered, name of intended registry	3
Trial registration: data set	#2b	All items from the World Health Organization Trial Registration Data Set	4
Protocol version	#3	Date and version identifier	3
Funding	#4	Sources and types of financial, material, and other support	12
Roles and responsibilities: contributorship	#5a	Names, affiliations, and roles of protocol contributors	1; 12

For peer review only

Roles and responsibilities: sponsor contact information	#5b	Name and contact information for the trial sponsor	12
Roles and responsibilities: sponsor and funder	#5c	Role of study sponsor and funders, if any, in study design; collection, management, analysis, and interpretation of data; writing of the report; and the decision to submit the report for publication, including whether they will have ultimate authority over any of these activities	13
Roles and responsibilities: committees	#5d	Composition, roles, and responsibilities of the coordinating centre, steering committee, endpoint adjudication committee, data management team, and other individuals or groups overseeing the trial, if applicable (see Item 21a for data monitoring committee)	11
**Introduction**			
Background and rationale	#6a	Description of research question and justification for undertaking the trial, including summary of relevant studies (published and unpublished) examining benefits and harms for each intervention	4
Background and rationale: choice of comparators	#6b	Explanation for choice of comparators	9
Objectives	#7	Specific objectives or hypotheses	4
Trial design	#8	Description of trial design including type of trial (eg, parallel group, crossover, factorial, single group), allocation ratio, and framework (eg, superiority, equivalence, non-inferiority, exploratory)	6
**Methods: Participants, interventions, and outcomes**			
Study setting	#9	Description of study settings (eg, community clinic, academic hospital) and list of countries where data will be	4

For peer review only

		collected. Reference to where list of study sites can be obtained	
Eligibility criteria	#10	Inclusion and exclusion criteria for participants. If applicable, eligibility criteria for study centres and individuals who will perform the interventions (eg, surgeons, psychotherapists)	7
Interventions: description	#11a	Interventions for each group with sufficient detail to allow replication, including how and when they will be administered	8
Interventions: modifications	#11b	Criteria for discontinuing or modifying allocated interventions for a given trial participant (eg, drug dose change in response to harms, participant request, or improving / worsening disease)	6
Interventions: adherance	#11c	Strategies to improve adherence to intervention protocols, and any procedures for monitoring adherence (eg, drug tablet return; laboratory tests)	10
Interventions: concomitant care	#11d	Relevant concomitant care and interventions that are permitted or prohibited during the trial	N/A
Outcomes	#12	Primary, secondary, and other outcomes, including the specific measurement variable (eg, systolic blood pressure), analysis metric (eg, change from baseline, final value, time to event), method of aggregation (eg, median, proportion), and time point for each outcome. Explanation of the clinical relevance of chosen efficacy and harm outcomes is strongly recommended	7
Participant timeline	#13	Time schedule of enrolment, interventions (including any run-ins and washouts), assessments, and visits for participants. A schematic diagram is highly recommended (see Figure)	10
Sample size	#14	Estimated number of participants needed to achieve study objectives and how it was determined, including clinical and statistical assumptions supporting any sample size calculations	7

For peer review only

Recruitment	#15	Strategies for achieving adequate participant enrolment to reach target sample size	7
**Methods: Assignment of interventions (for controlled trials)**			
Allocation: sequence generation	#16a	Method of generating the allocation sequence (eg, computer-generated random numbers), and list of any factors for stratification. To reduce predictability of a random sequence, details of any planned restriction (eg, blocking) should be provided in a separate document that is unavailable to those who enrol participants or assign interventions	6
Allocation concealment mechanism	#16b	Mechanism of implementing the allocation sequence (eg, central telephone; sequentially numbered, opaque, sealed envelopes), describing any steps to conceal the sequence until interventions are assigned	6
Allocation: implementation	#16c	Who will generate the allocation sequence, who will enrol participants, and who will assign participants to interventions	6
Blinding (masking)	#17a	Who will be blinded after assignment to interventions (eg, trial participants, care providers, outcome assessors, data analysts), and how	6
Blinding (masking): emergency unblinding	#17b	If blinded, circumstances under which unblinding is permissible, and procedure for revealing a participant's allocated intervention during the trial	10
**Methods: Data collection, management, and analysis**			
Data collection plan	#18a	Plans for assessment and collection of outcome, baseline, and other trial data, including any related processes to promote data quality (eg, duplicate measurements,	10

For peer review only

		training of assessors) and a description of study instruments (eg, questionnaires, laboratory tests) along with their reliability and validity, if known. Reference to where data collection forms can be found, if not in the protocol	
Data collection plan: retention	#18b	Plans to promote participant retention and complete follow-up, including list of any outcome data to be collected for participants who discontinue or deviate from intervention protocols	12
Data management	#19	Plans for data entry, coding, security, and storage, including any related processes to promote data quality (eg, double data entry; range checks for data values). Reference to where details of data management procedures can be found, if not in the protocol	10
Statistics: outcomes	#20a	Statistical methods for analysing primary and secondary outcomes. Reference to where other details of the statistical analysis plan can be found, if not in the protocol	10
Statistics: additional analyses	#20b	Methods for any additional analyses (eg, subgroup and adjusted analyses)	10
Statistics: analysis population and missing data	#20c	Definition of analysis population relating to protocol non-adherence (eg, as randomised analysis), and any statistical methods to handle missing data (eg, multiple imputation)	10
**Methods: Monitoring**			
Data monitoring: formal committee	#21a	Composition of data monitoring committee (DMC); summary of its role and reporting structure; statement of whether it is independent from the sponsor and competing interests; and reference to where further details about its charter can be found, if not in the protocol. Alternatively, an explanation of why a DMC is not needed	11
Data monitoring: interim analysis	#21b	Description of any interim analyses and stopping guidelines, including who will have access to these interim results and make the final decision to terminate the trial	11

For peer review only

Harms	#22	Plans for collecting, assessing, reporting, and managing solicited and spontaneously reported adverse events and other unintended effects of trial interventions or trial conduct	11
Auditing	#23	Frequency and procedures for auditing trial conduct, if any, and whether the process will be independent from investigators and the sponsor	11
**Ethics and dissemination**			
Research ethics approval	#24	Plans for seeking research ethics committee / institutional review board (REC / IRB) approval	3
Protocol amendments	#25	Plans for communicating important protocol modifications (eg, changes to eligibility criteria, outcomes, analyses) to relevant parties (eg, investigators, REC / IRBs, trial participants, trial registries, journals, regulators)	3
Consent or assent	#26a	Who will obtain informed consent or assent from potential trial participants or authorised surrogates, and how (see Item 32)	3
Consent or assent: ancillary studies	#26b	Additional consent provisions for collection and use of participant data and biological specimens in ancillary studies, if applicable	10
Confidentiality	#27	How personal information about potential and enrolled participants will be collected, shared, and maintained in order to protect confidentiality before, during, and after the trial	10
Declaration of interests	#28	Financial and other competing interests for principal investigators for the overall trial and each study site	13
Data access	#29	Statement of who will have access to the final trial dataset, and disclosure of contractual agreements that limit such access for investigators	10
Ancillary and post trial care	#30	Provisions, if any, for ancillary and post-trial care, and for compensation to those who suffer harm from trial	N/A

For peer review only

		participation	
Dissemination policy: trial results	#31a	Plans for investigators and sponsor to communicate trial results to participants, healthcare professionals, the public, and other relevant groups (eg, via publication, reporting in results databases, or other data sharing arrangements), including any publication restrictions	11
Dissemination policy: authorship	#31b	Authorship eligibility guidelines and any intended use of professional writers	N/A
Dissemination policy: reproducible research	#31c	Plans, if any, for granting public access to the full protocol, participant-level dataset, and statistical code	11
**Appendices**			
Informed consent materials	#32	Model consent form and other related documentation given to participants and authorised surrogates	N/A
Biological specimens	#33	Plans for collection, laboratory evaluation, and storage of biological specimens for genetic or molecular analysis in the current trial and for future use in ancillary studies, if applicable	Table 3

The SPIRIT Explanation and Elaboration paper is distributed under the terms of the Creative Commons Attribution License CC-BY-NC. This checklist was completed on 04. December 2021 using https://www.goodreports.org/, a tool made by the EQUATOR Network in collaboration with Penelope.ai

For peer review only

BMJ Open

Building knowledge, optimising physical and mental health, and setting up healthier life trajectories in South African women (Bukhali): a preconception randomised control trial part of the Healthy Life Trajectories Initiative (HeLTI)

Journal:	*BMJ Open*
Manuscript ID	bmjopen-2021-059914.R1
Article Type:	Protocol
Date Submitted by the Author:	10-Feb-2022

Complete List of Authors:	Norris, Shane; University of the Witwatersrand, SAMRC Developmental Pathways for Health Research Unit, Department of Paediatrics; University of Southampton, Global Health Research Institute, School of Health and Human Development<br>Draper, Catherine; University of the Witwatersrand, SAMRC Developmental Pathways for Health Research Unit<br>Prioreschi, Alessandra; University of the Witwatersrand, MRC/Wits Developmental Pathways for Health Research Unit<br>Smuts, CM; North-West University<br>Ware, Lisa; University of the Witwatersrand, Department of Paediatrics<br>Dennis, CindyLee; University of Toronto<br>Awadalla, Philip; University of Toronto, Department of Molecular Genetics, Ontario Institute for Cancer Research<br>Bassani, D; SickKids Research Institute, Centre for Global Child Health<br>Bhutta, Zulfiqar; SickKids Research Institute, Centre for Global Child Health<br>Briollais, Laurent; Lunenfeld-Tanenbaum Research Institute,<br>Cameron, D; Ottawa Hospital General Campus, Medicine, Division of Infectious Diseases; Ottawa Hospital Research Institute<br>Chirwa, Tobias; University of the Witwatersrand<br>Fallon, B; University of Toronto<br>Gray, CM; University of Stellenbosch, Division of Molecular Biology and Human Genetics; University of Cape Town, Division of Immunology, Institute of Infectious Disease and Molecular Medicine<br>Hamilton , Jill; University of Toronto, Department of Paediatrics<br>Jamison, J; Red River College<br>Jaspan, Heather; University of Cape Town, Division of Immunology, Institute of Infectious Disease and Molecular Medicine<br>Jenkins, Jennifer; University of Toronto, Department of Applied Psychology and Human Development<br>Kahn, Kathleen; University of the Witwatersrand<br>Kengne , AP; South African Medical Research Council<br>Lambert, Estelle; University of Cape Town, Division of Exercise Science and Sports Medicine<br>Levitt, Naomi; University of Cape Town<br>Martin , Marie-Claude ; University of Toronto<br>Ramsay, Michele; University of the Witwatersrand<br>Roth, Daniel; The Hospital for Sick Children; University of Toronto,

Department of Paediatrics
Scherer, Stephen; University of Toronto, Department of Molecular Genetics; The Hospital for Sick Children
Sellen, Daniel; University of Toronto; University of Toronto, Department of Nutritional Sciences
Slemming, Wiedaad; University of the Witwatersrand, Department of Paediatrics
Sloboda, Deborah; Hamilton, Department of Biochemistry and Biomedical Sciences
Szyf, M.; McGill University, Department of Pharmacology and Therapeutics
Tollman, Stephen; University of the Witwatersrand
Tomlinson, Mark; University of Stellenbosch, Institute for Life Course Health Research; Queen's University Belfast
Tough, Suzanne ; Alberta Children's Hospital Research Institute, Department of Paediatrics
Matthews, Stephen; University of Toronto, Department of Applied Psychology and Human Development; University of Toronto
Richter, Linda; University of the Witwatersrand, DSI-NRF Centre of Excellence in Human Development
Lye, Stephen; University of Toronto, Department of Applied Psychology and Human Development; University of Toronto

**Primary Subject Heading**:	Public health
Secondary Subject Heading:	Nutrition and metabolism
Keywords:	NUTRITION & DIETETICS, PUBLIC HEALTH, SOCIAL MEDICINE

SCHOLARONE™
Manuscripts

Building knowledge, optimising physical and mental health, and setting up healthier life trajectories in South African women (Bukhali): a preconception randomised control trial part of the Healthy Life Trajectories Initiative (HeLTI)

Norris SA[1], Draper CE[1], Prioireschi A[1], Smuts CM[2], , Ware L[1,28], Dennis CL[3], Awadalla P[4], Bassani DG[5], Bhutta ZA[5,6], Briollais L[7,19], Cameron B[8], Chirwa T[9], Fallon B[10], Gray CM[11,30], Hamilton J[12], Jamison J[13], Jaspan H[11], Jenkins JM[14], Kahn K[15], Kenge AP[16], Lambert E[17], Levitt N[18], Martin MC[19], Ramsay M[20], Roth, DE[5,12], Scherer SW[4,21], Sellen D[7,22], Slemming W[23], Sloboda DM[24], Szyf M[25], Tollman S[15], Tomlinson M[26,31], Tough S[27], Matthews S[14,19,28], Richter LM[29], Lye S[14,19,28]

Affiliations

1. SAMRC Developmental Pathways for Health Research Unit, Department of Paediatrics, Faculty of Health Sciences, University of the Witwatersrand, Johannesburg, South Africa
2. Centre of Excellence for Nutrition, North-West University, Potchefstroom, South Africa
3. Lawrence S. Bloomberg Faculty of Nursing, University of Toronto, Toronto, Canada
4. Department of Molecular Genetics, Ontario Institute for Cancer Research, University of Toronto, Toronto, Canada
5. Centre for Global Child Health, The Hospital for Sick Children, Toronto, Canada
6. Institute for Global Health and Development, Aga Khan University, Karachi, Pakistan
7. Dalla Lana School of Public Health, University of Toronto, Toronto, Canada
8. Ottawa Hospital Research Institute, University of Ottawa, Ottawa, Canada
9. School of Public Health, University of the Witwatersrand, Johannesburg, South Africa
10. Factor-Inwentash Faculty of Social Work, University of Toronto, Toronto, Canada
11. Division of Immunology, Institute of Infectious Disease and Molecular Medicine, Faculty of Health Sciences, University of Cape Town, Cape Town, South Africa
12. Department of Paediatrics, The Hospital for Sick Children, University of Toronto, Toronto, Canada
13. School of Health Sciences and Community Services, Red River College, Winnipeg, Canada
14. Department of Applied Psychology and Human Development, University of Toronto, Toronto, Canada
15. SAMRC Rural Public Health and Health Transitions Research Unit, School of Public Health, University of the Witwatersrand, Johannesburg, South Africa
16. Non-communicable Diseases Research Unit, South African Medical Research Council, Cape Town, South Africa
17. Division of Exercise Science and Sports Medicine, University of Cape Town, Cape Town, South Africa
18. Chronic Disease Initiative for Africa, University of Cape Town, Cape Town, South Africa
19. Lunenfeld-Tanenbaum Research Institute, Sinai Health System, University of Toronto, Toronto, Canada
20. Sydney Brenner Institute for Molecular Bioscience, University of the Witwatersrand, Johannesburg, South Africa
21. The Centre for Applied Genomics, The Hospital for Sick Children, Toronto, Canada; Program in Genetics and Genome Biology, The Hospital for Sick Children, Toronto, Canada; McLaughlin Centre, University of Toronto, Toronto, Canada
22. Department of Nutritional Sciences, University of Toronto, Toronto, Canada; Joannah and Brian Lawson Centre for Child Nutrition, University of Toronto, Toronto, Canada
23. Department of Paediatrics, University of the Witwatersrand, Johannesburg, South Africa
24. Department of Biochemistry and Biomedical Sciences, McMaster University, Hamilton, Canada
25. Department of Pharmacology and Therapeutics, McGill University, Montreal, Canada
26. Institute for Life Course Health Research, Department of Global Health, Stellenbosch University, Stellenbosch, South Africa
27. Department of Paediatrics, Alberta Children's Hospital Research Institute, Calgary, Canada
28. Department of Physiology, University of Toronto, Toronto, Canada; Departments of Obstetrics and Gynaecology and Medicine, University of Toronto, Toronto, Canada
29. DSI-NRF Centre of Excellence in Human Development, University of the Witwatersrand, Johannesburg, South Africa

For peer review only

30. Division of Molecular Biology and Human Genetics, Stellenbosch University, Cape Town, South Africa
31. School of Nursing and Midwifery, Queens University, Belfast, UK

Corresponding author: Professor Shane Norris; shane.norris@wits.ac.za

Email addresses:
Draper CE: Catherine.Draper@wits.ac.za
Smuts CM: Marius.Smuts@nwu.ac.za
Prioireschi A: Alessandra.Prioreschi@wits.ac.za
Ware L: lisa.ware@wits.ac.za
Dennis CL: cindylee.dennis@utoronto.ca
Awadalla P: Philip.Awadalla@oicr.on.ca
Bassani DG: diego.bassani@sickkids.ca
Bhutta ZA: zulfiqar.bhutta@sickkids.ca
Briollais L: laurent@lunenfeld.ca
Cameron B: BCAMERON@toh.ca
Chirwa T: Tobias.Chirwa@wits.ac.za
Fallon B: barbara.fallon@utoronto.ca
Gray CM: cgray@sun.ac.za
Hamilton J: jill.hamilton@sickkids.ca
Jamison J: janetijamieson@gmail.com
Jaspan H: hbjaspan@gmail.com
Jenkins JM: jenny.jenkins@utoronto.ca
Kahn K: Kathleen.Kahn@wits.ac.za
Kenge AP: Andre.Kengne@mrc.ac.za
Lambert E: vicki.lambert@uct.ac.za
Levitt N: Naomi.Levitt@uct.ac.za
Martin MC: mcmartin@lunenfeld.ca
Ramsay M: Michele.Ramsay@wits.ac.za
Roth, DE: daniel.roth@sickkids.ca
Scherer SW: stephen.scherer@sickkids.ca
Sellen D: dan.sellen@utoronto.ca
Slemming W: Wiedaad.Slemming@wits.ac.za
Sloboda DM: sloboda@mcmaster.ca
Szyf M: moshe.szyf@mcgill.ca
Tollman S: Stephen.Tollman@wits.ac.za
Tomlinson M: markt@sun.ac.za
Tough S: stough@ucalgary.ca
Matthews S: stephen.matthews@utoronto.ca
Richter LM: Linda.Richter@wits.ac.za
Lye S: LYE@lunenfeld.ca

For peer review only

Article summary

Introduction: South Africa's evolving burden of disease is challenging due to a persistent infectious disease, burgeoning obesity, most notably among women, and rising rates of non-communicable diseases (NCDs). With two thirds of women presenting at their first antenatal visit either overweight or obese in urban South Africa (SA), the preconception period is an opportunity to optimise health and offset transgenerational risk of both obesity and NCDs.

Methods and analysis: *Bukhali* is the first individual randomised controlled trial in Africa to test the efficacy of a complex continuum of care intervention and forms part of the Healthy Life Trajectories Initiative (HeLTI) consortium implementing harmonised trials in Canada, China, India and SA. Starting preconception and continuing through pregnancy, infancy and childhood, the intervention is designed to improve nutrition, physical and mental health, and health behaviours of South African women to offset obesity-risk (adiposity) in their offspring. Women aged 18-28 years (n=6800) will be recruited from Soweto, an urban-poor area of Johannesburg. The primary outcome is dual-energy x-ray absorptiometry derived fat mass index (fat mass divided by height^2) in the offspring at age 5-years. Community health workers will deliver the intervention randomly to half the cohort by providing health literacy material, dispensing a multi-micronutrient supplement, providing health services and feedback, and facilitating behaviour change support sessions to optimise: (i) nutrition, (ii) physical and mental health, and (iii) lay the foundations for healthier pregnancies and early child development.

Ethics and dissemination: Ethical approval has been obtained from the Human Ethics Research Committee University of the Witwatersrand, Johannesburg, South Africa (M1811111), the University of Toronto, Canada (19-0066-E) and the WHO Ethics Committee (ERC.0003328). Data and biological sample sharing policies are consistent with the governance policy of the HeLTI Consortium (https://helti.org) and South African government legislation (POPIA). The recruitment and research team will obtain informed consent.

Strengths and limitations of the study

- *Bukhali* will be the first RCT in Africa to evaluate if a community health worker facilitated intervention starting preconception will reduce offspring obesity risk at age 5-years.
- Primary and many secondary outcomes are harmonised with three other HeLTI trial-sites (Canada, China and India) to enable comparative and pooled analyses.
- The impact of *Bukhali's* data and learning will be to inform preconception prevention of NCD-risk strategies in Africa.
- A limitation is that *Bukhali* does not include an economic evaluation.

Trial registration: this trial is registered with the Pan African Clinical Trials Registry (https://pactr.samrc.ac.za) on 25 March 2019 (identifier: PACTR201903750173871). See Supplementary Table 1

Protocol version: 8 November 2021 (version #3). Any protocol amendments will be communicated to investigators, IRBs, trial participants, and trial registries.

Keywords: pre-conception, South Africa, childhood obesity, early child development, healthy trajectories

For peer review only

Background

Non-communicable diseases (NCDs) such as obesity, hypertension, and diabetes are significant health burdens in many rapidly transitioning middle-income countries[1]. NCDs kill 41 million people each year (71% of deaths) globally, and WHO has identified NCDs as one of the ten leading threats to health[2]. From 1980 to 2014, the prevalence of diabetes changed little in western Europe, but doubled in sub-Saharan Africa (SSA), India and China[1]. SSA countries have persisting afflictions from infectious diseases (HIV, TB and malaria) against a backdrop of maternal and child malnutrition[3], but also now the COVID-19 pandemic. In many respects the impetus to address NCDs as part of the Sustainable Development Goals has stalled as a result of the COVID-19 emergency.

Childhood weight gain, overweight and obesity, and adiposity are risk factors for poor health trajectories and development of NCDs. The number of children and adolescents with obesity has risen to 124 million worldwide and could exceed 250 million by 2030 without substantial interventions[4]. A meta-analysis of 61 studies showed that increased preconception and interpregnancy weight gain was associated with gestational diabetes (88% greater risk), preeclampsia (92% greater risk), and large-for-gestational-age (36% greater risk) babies[5]. A systematic review of 38 studies found a consistent association between maternal pre-pregnancy weight and offspring obesity[6]. Furthermore, Heslehurst and colleagues showed that a child has a 2.6-fold greater risk of obesity if the mother is obese prior to conception[7]. A systematic review by Dean and co-authors identified 23 randomised controlled trials of preconception care and counselling delivered via health facilities or community groups, that reported improved women's health behaviours (calorie restriction and increased physical activity), as well as, more positive maternal, neonatal and child health outcomes including an increase in antenatal care seeking and an increased likelihood of breast feeding[8,9]. None of these preconception interventions were conducted in Africa.

South Africa's (SA) evolving burden of disease is complex due to: (i) persisting malnutrition; (ii) the highest prevalence of overweight and obesity in SSA, particularly among women; and (iii) rising rates of non-communicable diseases such as of type 2 diabetes (T2D). In Soweto (urban-poor area of the Johannesburg Metropole covering 200 km^2 with over 1.3 million residents; 6400/km^2) we have found that the prevalence of overweight and obesity in females increases from 10% by age 8 to 43% by age 22 years, while the prevalence in males remains fairly constant into young adulthood at 10%; further, that girls who are obese by age 5 are 42 times more likely to be obese adults[10,11]. Furthermore, 66% of pregnant Soweto women at first antenatal clinic visit, were either overweight or obese, and 10% were diagnosed with gestational diabetes (GDM) in late pregnaancy[12,13]. Consequently, there is a high obesity burden need in Soweto for preconception interventions to support women to optimise health and manage weight to combat intergenerational obesity[14].

The Healthy Life Trajectory Initiative (HeLTI), an international consortium developed in partnership with the WHO to address childhood obesity. We hypothesise that an integrated complex intervention comprising a continuum of care starting pre-conception and across this period of life (pre-pregnancy, pregnancy, infancy and childhood) will reduce childhood adiposity and the risk for NCDs, as well as, improve child development. Four randomised controlled trials in Ontario and Alberta (Canada)[15], Shanghai (China)[16], Mysore (India)[17], and Soweto (South Africa) have been harmonised to test this hypothesis. These sites were selected based on the community need and that these sites possessed both the infrastructure, multidisciplinary teams and cohort/trial experience to execute an ambitious ten-year programme of work. This paper describes the HeLTI South African trial protocol in Soweto called *Bukhali* (a Zulu word meaning smart/powerful) signifying the beneficial potential of optimising women's health for herself and her future offspring.

Methods/design

Participant and public involvement

We constituted a Community Advisory Group with local women, and have involved them in the intervention development and the preparatory phase to enable feedback and input and to facilitate greater acceptance and feasibility of the study. Furthermore, a Participant Advisory Group has been formed as part of a longitudinal qualitative study to further understand and identify preconception health concerns, barriers and solutions, as well as other contextual issues that participants are facing

which could influence their participation in the trial. A Stakeholder Advisory Group has been formed with multiple stakeholders (South African government departments of health and education, UNICEF, Save the Children, WHO, NGOs, and academics) to share the development of *Bukhali* and elicit input and guidance around a variety of issues – policy relevance, linkage with current initiatives, ethics, and dissemination.

Formative research and pilot trial study

The trial was designed following the Standard Protocol Items (Recommendations for Interventional Trials (SPIRIT) 2013 statement). Through extensive qualitative (focus groups, in-depth individual interviews, and stakeholder engagement)[18-23] and epidemiological research[24-31], we identified our young women (18-28 years) as the target group given that a high proportion these women are already either overweight or obese and most will have their first child during this age range, and developed the intervention package. We initially opted for a cluster randomised study design in order to included community peer group sessions as part of the intervention. To assess the feasibility and implementation of this design, a pilot trial of data collection and intervention commenced in 2018 and concluded in 2019. We recruited 1719 participants within six community clusters, randomised the clusters, collected baseline data and biological samples, and implemented the planned intervention and control arm activities. The pilot trial data highlighted critical learning, among others, that: (i) it was feasible to recruit and collect good quality data and biological samples (95% consented to provide a blood sample and 75% consented to provide a DNA sample) from a large group of our target population; (ii) young women living in Soweto lead complicated and economically stressful lives and are pre-occupied with survival strategies, which often entails moving and living at multiple homes across family and friends in the community over time; (iii) there is poor health literacy around preconception health amongst young women in Soweto; (iv) young women were anxious about participation due to fear of criminal activities and abduction; (v) community safety of study community health workers could not be assured; and (vi) we could not effectively deliver the planned community peer group component due to poor attendance despite convenient Saturday meeting times, multiple opportunities during the month to attend, and the provision of refreshments. To address these challenges, we formulated a multi-pronged strategy: (i) we rolled-out a communication campaign; a soap opera series of 11 episodes drawing attention to preconception health issues in multiple languages through two local radio stations with the largest population reach supported by social media engagement (https://soundcloud.com/user-662329775); (ii) we engaged and obtained additional support to operate within the community through community leadership forums and police awareness of the study team; (iii) we produced branded material for staff and vehicles with clear university and project identification to promote credibility; and (iv) we modified the study design (we pivoted from cluster-randomisation to individual-randomisation due to cluster contamination linked to young women shifting between multiple households) and intervention delivery, and jettisoned the peer group component because of poor compliance[32].

Study design

Bukhali is an individual-randomised controlled two-arm trial, (simple randomisation; 1:1 ratio to the intervention arm or to standard of care plus control arm). After informed consent and baseline data collection, the participant will move to a separate/private randomisation counter where the participant clicks on a computer-generated electronic envelope that reveals the allocation to the participant and the randomisation officer. The randomisation officer then escorts the participant to a separate wing on a different floor in the centre to introduce participant to either the intervention arm or control arm coordinator, thus preserving the concealment of allocation. The research trial data team and associated researchers will be blinded to participant allocation throughout the trial, and this will be achieved by: (i) ensuring that group allocation is locked on the database and cannot be accessed by the research team; (ii) training the research team not to ask and participants will be encouraged not to divulge their allocation status; and (iii) when participants visit the centre for their respective arm programme these activities will occur in a separate part of the centre away from the research team. All participants will be exposed to the preconception intervention or control programme for up to 18 months. If a participant does not become pregnant within the 18 months, she will have an exit assessment. If a participant becomes pregnant, she will exit the preconception phase and be monitored through pregnancy, and postnatally. An 'index child' conceived after randomisation will be followed until age 5-years. Participant flow through the trial is seen in Figure 1.

For peer review only

Insert Figure.1

Inclusion/exclusion criteria

Participants will be recruited through a survey of Soweto households (approximately 30000), which is sufficiently large to adequately recruit our target participant number. Women aged 18-28 years are eligible, unless they are: (i) diagnosed with type-I diabetes or epilepsy because these require intensive treatment and management priorities; (ii) present with intellectual disability that hinders informed consent, and (iii), not able or willing to provide consent. Pregnancy at the time of the baseline assessment does not exclude participation. Participants who move residence during the course of the study will continue participation as long as their current address is known, the participant wishes to remain involved, and data can be collected. Participants who have miscarriages will be offered counselling support and remain in the study.

Sample size

The sample size calculation is based on the mean (4.1 kg/m^2) and SD (2.1) for dual-energy x-ray absorptiometry (DXA) derived fat mass index (FMI) calculated from our pre-pubertal data from the Birth to Twenty Plus Cohort in Soweto, South Africa. We will require 765 children to be born in each arm to detect a meaningful difference of 0.25 SD (0.5 kg/m^2) in FMI at age 5-years between the intervention and control groups at 80% power and 5% significance level, and assuming 30% attrition. Therefore, we will recruit approximately 6800 women so reach a target of 1530 pregnancies. The intervention group will receive all four phases of the intervention and the control remains standard of care plus throughout. Pooling of data across the four countries will provide the ability to detect an effect size of 0.1 SD between intervention and control groups at 80% power and 5% significance level. The pooled analysis should also allow us to detect a 0.2 SD difference in our primary outcome in boys and girls separately (although this is dependent on the direction of change being the same across our populations).

Primary and secondary outcome questions

Primary child outcome: What is the relative effect of a 4-phase intervention on adiposity status at age 5-years as determined by DXA-derived fat mass index (fat mass/height^2) among children of young adult women in the intervention arm, when compared to the children of women who receive standard of care plus (control arm)?

Secondary child outcomes: What are the effects of the 4-phase intervention on child outcomes at age 5-years on:

1. Anthropometry (age- and sex-standardized continuous BMI-z score, BMI growth trajectories, continuous waist circumference z- score, upper arm circumference, head circumference);
2. Cardiometabolic outcomes (systolic and diastolic blood pressure, lipid profile, glucose, insulin, insulin resistance);
3. Behaviour (dietary intake, physical activity, screen time, sedentary behaviour, sleep time);
4. Developmental outcomes (cognitive, motor skills, communication, behavioural); and

Secondary maternal outcomes: What are the effects of the 4-phase intervention on?

1. Anthropometry (overweight and obesity (OWO), DXA-derived body composition, waist circumference);
2. Nutrition (anaemia)
3. Physical health (hypertension);
4. Mental health (depressive symptoms, anxiety) and
5. Behaviour (dietary patterns, physical activity, screen time, sedentary behaviour, sleep time, tobacco and alcohol use);

Phase-specific outcomes

1. Preconception Phase (outcome at conception or exit): What is the effect of the preconception intervention phase on: maternal anthropometry and body composition (overweight and obesity, fat mass index, waist circumference); nutrition (anaemia); health behaviour (physical activity, screen time, sleep); mental health (depressive symptoms, anxiety); and physical health (hypertension)?

For peer review only

2. Pregnancy Phase (parental and child outcomes at birth): What is the effect of the preconception + pregnancy intervention phases on: pregnancy weight gain; rate of gestational diabetes; rate of gestational hypertension; rate of pre-eclampsia; rate of preterm birth < 37 weeks; rates of large- and small- birthweight for gestational age; and maternal mental health?
3. Infancy Phase (parental and child outcomes across the first two years postpartum): What is the effect of the preconception + pregnancy + infancy intervention phases on: breastfeeding outcomes (initiation, duration, exclusivity, self-efficacy); nutrition (anaemia), health behaviour outcomes (physical activity, screen time, sleep); and mental health outcomes (depressive symptoms, anxiety), and child outcomes (growth, blood pressure, sleep, sedentary and physical activity; feeding practices; developmental outcomes)?
4. Early Childhood phase (parental and child outcomes 2-5 years postpartum): What is the effect of the preconception + pregnancy + infancy +childhood intervention phases on: maternal nutrition (anaemia), health behaviour outcomes (physical activity, screen time, sleep); and mental health outcomes (depressive symptoms, anxiety); and child outcomes (growth, blood pressure, sleep, sedentary and physical activity; dietary practices; cognitive development outcomes)?

Ethics and regulatory management

Bukhali has ethical approval from the Human Ethics Research Committee of the University of the Witwatersrand, Johannesburg, South Africa (M1811111), the University of Toronto, Canada (19-0066-E) and the WHO Ethics Committee (ERC.0003328). The trial is registered with the Pan African Clinical Trial Registry.

Intervention: community health worker approach

The intervention has been informed by the UK MRC Guidelines for Complex Interventions, and we used the TIDieR (Template for Intervention Description and Replication) Checklist. Using the taxonomy of behaviour change techniques (BCTs)[33], we grounded the behaviour change intervention in: (i) theory of planned behaviour, which suggests that behaviours are determined by attitudes, subjective norms and perceived control; (ii) control theory, which suggests we use inner- and outer-control to minimise deviation from the standard; and (iii) social cognitive theory, which describes that behaviour and choices are influenced by the environment, personality and other factors such as goals. These theories combined anchors the intervention to value the importance of context, individual needs, and support to bolster agency. Figure 2 details the original logic model to illustrate the pathways to impact that guided the intervention development. The intervention is centred on "Living your best life" with four universal objectives that infuse through all phases of the intervention (preconception, pregnancy, infancy and childhood) to: (i) optimise health; (ii) optimise nutrition; (iii) optimise mental health; and (iv) promote early childhood development. The agents of change are modelled upon community health workers (CHWs), who we call "Health Helpers" (HHs) to differentiate them from current Department of Health workers. We opted for the CHW approach as there is strong efficacy evidence in Africa, and it aligns with SA's primary health care re-engineering policy. The HHs will share similar qualifications, characteristics and salary levels to CHWs in SA, and eligibility includes: women between the ages of 23-40 years with completed secondary school with some tertiary education, but no formal training as a CHW is required.

The HHs are supported by a supervisor and dietician with access to nurses and a referral network. HHs deliver the intervention through the provision of health literacy resource materials and micronutrient supplements, provide health feedback and services, and they support behaviour change through individual sessions in person or telephonically, and supportive text messaging. Multiple strategies will be put in place by the HHs (flexibility, reminders, home visits, incentives) to aid in achieving trial compliance.

Insert Figure 2.

Healthy Conversation Skills and trauma-informed care: HHs will be trained in Healthy Conversation Skills (HCS), a technique specifically developed and tested for use with socioeconomically disadvantaged participants to improve self-efficacy and support behaviour change. HCS is based on a social-cognitive model of health behaviour, which emphasises the role of increasing self-efficacy in promoting behaviour change[34-36]. It recognises that knowledge alone is insufficient to

change behaviour unless people are also motivated and empowered to change. Trained in HCS, HHs will explore the barriers that that women experience in trying to change their behaviour, ways of over-coming these barriers and setting goals for change. The HCS approach enables a woman to reflect on her particular priorities for physical and mental health, and to support her in finding her own solutions to challenges she experiences. The training is standardised and supported by the MRC Lifecourse Epidemiology Unit (UK). HCS training is aimed at achieving four basic competencies that are known to be useful in supporting behaviour change: (i) the use of open-discovery questions, questions that begin with 'how' and 'what' and lead people to explore and find their own ways of overcoming barriers to change; (ii) listening more than talking which allows people to identify and take control of their own behaviour change; (iii) reflecting on practice in order to be more effective; and (iv) supporting the use of SMARTER goal setting so that participants have a sense of change and progress. To the HCS approach we have added selected skills from the WHO Problem Management Plus (individual psychological help for adults impaired by distress in communities exposed to adversity).

Health literacy resource material: With topic experts and a specialised curriculum developer, we integrated behaviour change theory and designed both HH facilitator manuals and participant resource materials. The resource material encompasses 9 modules (women's health, chronic diseases, diet, sleep, physical activity and fitness, sitting time, body size and image, emotional awareness, and caregiving) in 6 chapters, and these modules repeat with new content every 6 chapters. The resource books contain essential educational content, local narratives to convey key concepts, and space for participants to engage with the material through the use of checklists, diaries and reflections. The approach for each module is that of: (i) Knowing (what do I know about it?); (ii) Doing (what am I doing about it?); and (iii) Becoming (how do I live my best life?). Three preconception health resource books are provided; one book is provided for pregnancy health, and supplementary resource materials to the South African Road to Health Booklet (https://sidebyside.co.za/resources/road-to-health-book/) and nurturing care resources are provided for infancy and early childhood.

Multi-micronutrient supplementation: based on 6-monthy point-of-care haemoglobin concentration assessments, the HHs will dispense and monitor micronutrient supplement use. The MMN supplement is based on WHO recommendations (Table 1) to optimise nutrition and address anaemia, based on haemoglobin concentrations:

- Non-anaemic women (Hb ≥12 g/dL at enrolment): Provision of a micronutrient supplement containing approximately 27 mg iron twice (2x) per week.
- Mildly anaemic women (Hb <12 g/dL and >7g/dL at enrolment): Provision of a micronutrient supplement daily for six months.
- Severely anaemic women (Hb <7g/dL at baseline): Receive treatment regime according to current standard of care in South Africa.

Supplement use will continue throughout the preconception period until pregnancy or exit. If the participant becomes pregnant, the supplement regimen will then shift to daily use throughout pregnancy and 6-months post-partum.

Table 1: Nutrient composition of multiple micronutrient supplements

Micronutrient	Unit	Nutrient Reference Value (NRV) EU
Vitamin A	2664 IU	100% (800 µg / 2664 IU)
Vitamin D3	200 IU	100% (5 µg / 200 IU)
Tocopherol (Vitamin E)	15 mg	125% (12 mg / 17.9 I
Vitamin K1	55 µg	73% (75 µg)
Thiamine	1.4 mg	127% (1.1 mg)
Riboflavin	1.4 mg	100% (1.4 mg)
Niacinamide	18 mg	113% (16 mg)

For peer review only

Pantothenic acid	6 mg	100% (6 mg)
Pyridoxine (Vitamin B6)	1.9 mg	136% (1.4 mg)
Biotin	30 µg	60% (50 µg)
Folic acid	600 µg	300% (200 µg)
Cyanocobalamin (Vitamin B12)	2.6 µg	100% (2.5 µg)
Ascorbic acid	60 mg	75% (80mg)
Copper	1.15 mg	100% (1 mg)
Iodine	250 µg	167% (150 µg)
Iron	27 mg	193% (14 mg)
Selenium	50 µg	100% (55 µg)
Zinc	10 mg	100% (10 mg)

Services: Through individual in-person or telephonic sessions, HHs will provide: (i) specific health feedback to participants based on their baseline results (BMI (obesity), blood pressure (hypertension), haemoglobin concentration (anaemia), mental health, and lifestyle (physical activity, sedentary behaviour, sleep); (ii) offer home-based HIV and pregnancy testing with counselling, utilise HCS to identify aspects of health behaviour change, set goals and monitor, and provide access to healthcare referral networks. For overweight and obese women, additional waist circumference measurements will be provided. During the infant and child periods, HHs continue the monthly consultations and support women with aid of the South African Road to Health Booklet and postnatal clinic visits.

Control arm: call centre approach (standard of care plus)
In addition to access to standard of care offered through the public health system, control arm participants will have a dedicated call centre. The rationale to add a standard of care plus programme is to maintain contact with participants, and minimise attrition bias. Similar to the intervention team, the call centre team will receive training in the delivery of the modules. To partially control for special attention given to women in the intervention arm, the call centre will contact participants once a month and deliver a curriculum on "life skills" via telephone, SMS and email. The non-health specific intervention material will be standardised and the modules of the curriculum will include: (i) civic rights and responsibilities (How do I apply for an identity document; what does it mean to vote?); (ii) financial education (savings & budgets; how do I open a bank account); (iii) job readiness (preparing a CV; job interview skills; volunteering); (iv) navigating social media (internet, different social media; how social media can be harmful), and (v) social grants. HIV and pregnancy testing services will be offered on request for free at the research centre.

Process evaluation
For all intervention (health worker-centric) and control (call centre-centric) components, Standard Operating Procedures (SOPs) support the HHs and call centre team to deliver their respective modules in a structured and uniform way. We will follow the MRC guidance on process evaluation of complex interventions[37]. Evaluation will focus on implementation, mechanisms of impact and context. Assessment of implementation will focus on finding out what is delivered and how delivery is achieved, and which is vital information if the intervention is to be replicated. We will assess compliance by counts using a log (for example, contacts made), and by discussions between HHs and participants. We will measure fidelity of intervention delivery by observing HHs during one-to-one contacts with intervention group participants, focusing on HHs use of HCS and the competencies they demonstrate. We will also observe the contact between call centre assistants and control participants to record the interactions. We will monitor intervention dose by recording the frequency and duration of contact between participants and HHs using diaries. To understand the mechanisms through which the intervention brings about change in the trial's specified outcomes, we will assess the acceptability of the intervention, and women's experiences of contact with HHs using interviews and focus groups. In assessing the context, we will aim to identify factors that might act as barriers or facilitators to intervention implementation or effects. This will include local and national policy, local

For peer review only

service configuration and provision, and socio-demographic and environmental factors within the communities taking part in the study. Qualitative methods (interviews and focus groups), including longitudinal approaches, will be used to further explore specific issues of relevance, in terms of participants' experiences of the trial, key issues facing participants, and factors influencing implementation of the trial.

Data collection, management and analysis

Table 2 and 3 details the phenotype measures and biological sample collection on women, biological fathers and index child. Formative research has indicated low-level (10%) biological father participation in the study. A dedicated research team will be established increase father participation through alternate approaches (for example: data collection flexibility in evening and on Saturdays; encouraging fathers to accompany pregnant women for their ultrasound scan). There is substantial harmonisation of data, samples and quality management across the four HeLTI studies. For example, over 1800 core variables have been harmonized from preconception through to infancy (6 months). All data will be managed through real-time capturing, reporting and quality control utilising REDCap electronic data capture tools[38]. Maelstrom Research at McGill University (Canada), has been supporting the harmonisation process across the sites and developing the online catalogue and metadata, which are available on their website (https://www.maelstrom-research.org/study/helti-hp). In addition, Maelstrom will support the implementation of a data infrastructure within each country using DataSHIELD/Opal software[39] to allow federated data analysis (country-specific or meta-analysis by pooling estimates across countries) to be conducted without having access to individual participant data or exporting and pooling data on a central server. This capacity will provide a mechanism for addressing restrictive regulation regarding exporting data across countries. The data management system coupled with our laboratory management system of samples (different tissues at different time points) will enable researchers to conduct future mechanistic studies and participants have provided consent for this on provision of IRB approval for such studies. All personal data are managed in a separate and secure with restricted access in line with the Protection of Personal Information Act of South Africa.

Analysis plan

Standard data screening/cleaning procedures will be conducted including the extent and pattern of missing data. When data are missing at random (MAR), which we expect to be the case, we will utilise multiple imputation so as to maximise sample size and study power in a dataset. We will assess the comparability at randomisation of the groups using descriptive analyses. We will also assess process indicators including recruitment and retention, and indicators of programme delivery in the Intervention Group. Sensitivity analyses will be performed to assess the influence of attrition and compliance. Intention-to-treat analysis will be used for key outcomes based on all viable pregnancies adjusted for compliance and selection of viable pregnancies to minimise bias. For the primary outcome, we will assess the effect of the intervention on fat mass index at age 5-years. The concept of this multiple-phase trial, is that the outcomes of interest in a particular phase result from the intervention administered in the same phase, but is also mediated by the outcomes observed in the previous phase(s). For example, a reduction in obesity risks during pregnancy caused by the intervention in this phase will impact birth weight and the child weight/BMI observed in the next phase. Therefore, at the end of the study, we will measure an "overall" effect of the interventions over the four phases on the final outcomes, but also, could assess how the association between the final outcomes and the interventions is mediated by the phase-specific outcomes (see Figure 3). Other secondary anthropometric outcomes to be assessed at 5-years of age include OWO, BMI and centiles, and adiposity (skin-fold thickness, arm circumference). We will also assess the effect of the intervention on glucose metabolism, blood pressure and cognitive outcomes. Mixed models with random intercept/slope effects and fixed effects (for the intervention) will be used to estimate the intervention effect. Due to possible heterogeneity of effects for key outcomes according to the time of initiation of the intervention (e.g. preconception versus pregnancy) we will conduct a subgroup analysis stratifying by this variable, testing for heterogeneity. The distribution of modifiable risk factors for childhood obesity, poor cardiometabolic and neurodevelopmental outcomes at baseline, and their changes over time will be assessed according to study group.

Insert Figure 3.

Table 2. Longitudinal data collection measures (M-mother, F-Father, C-Infant/child)

Domain	Measure	Preconception		Pregnancy (week)		Postnatal period (months)				
**Time point (months)**		**Baseline**	**18 month (exit)**	**10-17**	**24-28**	**Delivery**	**6**	**12**	**24**	**60**
**Demography**	Demographic and socio-economic data, household composition, occupation, education, living environment	M	M	M	F		M	M	M	M
	Date of birth / sex	M			F	C				
**Anthropometry and body composition**	Height/length	M			F	C	C	C	C	C
	Weight	M	M	M	M/F	C	M/C	M/C	M/C	M/C
	Gestational weight gain			M	M					
	Body Mass Index	M	M	M	M/F		M	M	M	M
	Adiposity: DXA (Adult)	M	M		F		M		M/C	M/C
	Skinfolds (triceps & subscapular) (*if scan is not possible)			M	M		M*		M*	M*
	Peapod body composition					C	C			
	Waist circumference	M	M		F		C	M/C	M/C	M/C
	Fetal anthropometry (gestational age and fetal growth)			C	C	C (GA)				
	Mid upper arm circumference	M	M	M	M/F	C	C	C	M/C	M/C
	Head circumference					C	C	C	C	C
**Clinical**	Pregnancy Complications			M	M					
	Birth complications, survival					M/C	C	C	C	C
	APGAR score 1 & 5 mins					C				
	HIV test			M	F					M
	Pregnancy test (pre-DXA scan)	M	M				M		M	M
	Blood pressure	M	M	M	M/F		M	M	M/C	M/C
	Anaemia (HemoCue)	M	M	M	M/F			M	M	M
	HbA1c	M	M	M	M/F			M	M	M
	Random blood glucose	M	M	M	F					
	Fasting blood glucose				M			M	M/C	M/C
	Oral glucose tolerance test				M					M/C
	Plasma & Serum collection	M	M	M	M/F			M	M/C	M/C
	DNA collection	M	M	M	M/F			M		M/C
	Urine spot sample	M	M		M/F				C	M/C
	Urine dipstick	M	M	M	M/F				C	M/C
	Heel-prick blood								C	
	Buccal swab								C	

For peer review only

**Lifestyle and health behaviour**	Diet intake (Dietary diversity & Household Food Insecurity Access Scale)	M	M	M	M/F			M	M	M
	Breastfeeding Questionnaire					M				
	Feeding (breastfeeding, solid food introduction, diversity, eating behaviour; SUNRISE Questionnaire)						C	C	C	C
	Physical activity (M:GPAQ & Sitting Time Quesstionnaire)	M	M	M	M/F			C/M	**M**	M
	Step Test		M		F					
	Accelerometry (sub-study)								C	C
	Screen Time Questionnaire	M	M	M	M/F		C	C	C	C
	Sleep: Pittsburgh Sleep Quality Index	M	M	M	M/F		M	M	M	M
	Brief Infant Sleep Questionnaire						C	C	C	
	SUNRISE sleep questionnaire									C
	Tobacco, alcohol, and drug use: Exposure questionnaire (WHO-STEPS, AUDIT-Questionnaire)	M	M	M	M/F			M	M	M
	Environmental tobacco exposure				M			C	C	C
	Child Care Questionnaire						M	M	M	M
**Mental and physical health, and chid development**	Depression (PHQ9)	M	M	M	M/F			M		M
	Depression (Edinburgh)			M	M		M			
	Stress (PSS)			M	M/F		M		M	M
	Stressful life events (ACEs)	M			F					
	Generalised Self-Efficacy Scale	M	M	M	M/F			M		M
	Social Support Questionnaire	M	M	M	M/F					
	Social Provisions Scale				M					M
	Medical history (and TB), family history, drug, supplement history	M	M	M	M/F		M	M	M	M
	Breasfeeding efficacy				M					
	Anxiety (GAD-7)	M	M	M	M/F			M	M	M
	Medication/supplement use	M	M	M	M/F	M/C	M/C	M/C	M/C	M/C
	Medical events (hospitalisation)				M	M/C	M/C	M/C	M/C	M/C
	Births, pregnancy & reproductive health	M		M		M	M	M	M	M
	Vaccinations						C	C	C	C
	Emotional health Questionnaire	M	M	M	M/F					
	Ages and Stages questionnaire						C	C	C	C
	Parenting practices (Brigance Parent-Child Interaction Scale)							M	M	

For peer review only

	Neurodevelopment (WHO Global Scale for Early Development - short-form)							C	C	
	Executive function (Early Years Toolbox)									C
	Temperament/behaviour (Strengths and Difficulties Questionnaire)								C	C
	School readiness (International Development and Early Learning Assessment)									C
	Stimulation in the home (UNICEF Multiple Inidcators Cluster Survey Early Childhood Development, Home Learning Environment)								C	C

Governance and monitoring

The shared governance and harmonisation structures for the HeLTI trials include: Research Committee of country Principal Investigators; a single Data Monitoring Committee that is independent from the sponsor and competing interests (DMC Charter is available on the HeLTI website); WHO Monitoring and Evaluation Team; and a HeLTI Secretariat. The trial is blinded to the investigators and research data collection team, but the data management team will unblind participant's allocation upon request by the DMC or ethics committee for key reporting or in cases of risk. A governance framework has been developed and adopted by all partners that details the management and access of data and biological samples. The HeLTI website (https://helti-net.org) provides more information including contact details of the HeLTI office and secretariat and information on the Data Access Portal through which investigators from around the world can view and request access to data/biospecimens from individual HeLTI country datasets. HeLTI will also be registered as a hub on Global Health Network (https://tghn.org) The Research Committee is supported by seven cross-consortium active working groups, including: (i) Core Variable Harmonization, (ii) Interventions and Retention, (iii) Early Child Development Measures, (iv) Biospecimen Collection and Management, (v) Data Management and Quality Assurance, (vi) Capacity Building, and (vii) Publications. Results will actively be disseminated within the four countries and through WHO mechanisms. An independent clinical research associate will audit the trial annually. WHO aims to regularly review quality assurance data of the trial. An electronic management system is in place for trial HHs and staff to record and manage adverse events. Utilising REDCap, Adverse Event Forms will be completed online, which will trigger a process of alerts and recommendations from the investigators, researchers and project coordinators to best handle the event. All events are also discussed at the HH weekly debrief session and reported to the DMC.

For peer review only

Table 3. Longitudinal biological sample measures

	Preconception		Pregnancy (weeks)		Postnatal period (months)		
	Baseline	18 month	10-17	24-28	12	24	60
**Mother**							
**Blood collection**	X (random)	X (random)	X (random)	X (fasting; OGTT)	X (fasting)	X (fasting)	X (fasting; OGTT)
**Plasma**	X	X	X	X	X	X	X
**Serum**	X	X	X	X	X	X	X
**DNA**	X		X	X	X		X
**Urine (spot sample)**	X	X		X			X
**Urine (dipstick)**	X	X	X	X			X
**Father**							
**Blood - random**				X			
**Plasma**				X			
**Serum**				X			
**DNA**				X			
**Urine (spot sample)**				X			
**Urine (dipstick)**				X			
**Infant/Child**							
**Blood collection (DBS)**					X		
**Blood collection**						X (fasting)	X (Fasting; OGTT)
**Plasma**						X	X
**Serum**						X	X
**DNA**							X
**Buccal swab**						X	
**Urine (spot sample & dipstick)**						X	X

Trial status and impact of the Covid-19 pandemic

Trial formative research started in April 2017, the pilot trial was initiated in 2018, main trial recruitment began in October 2019 and is expected to conclude early in 2022, and the trial is expected to be completed by 2029. The collective HeLTI initiative provides an unprecedented preconception health platform with the potential to expand to other settings. To maximise the learning and impact of these studies, HeLTI aims to host postgraduate students and training opportunities, and facilitate accelerated managed access to data and biological samples. Our formative work has revealed just how complex both the environments and lives of young urban women in South Africa are. The health implications are further compounded by poorer than anticipated health literacy levels. Among these women, health is not prioritised amidst competing economic and food security concerns, and the daily stress of navigating challenging household (relationships, households with multi-families) and community circumstances (poverty, crime and exposure to violence). Also, the Soweto environment is becoming more obesogenic with easier access to less healthy fast foods that are affordable and sold on most street corners. This makes the decision to purchase healthier, more expensive foods that are sold further away less appealing[20].

For peer review only - http://bmjopen.bmj.com/site/about/guidelines.xhtml

For these reasons, we have opted for a more pragmatic and adaptive trial design, whereby the intervention package can be modified to the circumstances of each participant and can incorporate new learning on how best to engage, support and improve compliance. The strength of this approach is to remain relevant and responsive to the urban poor context, and thereby, increase the likelihood of achieving health behaviour change. The challenge of this adaptive and individually-responsive approach is how to capture and analyse the dose effect of intervention elements. To address this challenge, we have setup a sophisticated data management and dashboard system which can monitor key quality assurance, delivery and compliance indicators that are not only useful for trial management, but also assist the intervention and control arm teams.

Six months into the main trial the Covid-19 pandemic hit South Africa through a series of waves and lockdown levels. A hard lockdown (level 5) was implemented in South Africa from 26 March - 30 April 2020. Thereafter a level 4 lockdown was implemented until 31 May 2020. Level 3 then lasted until 17 August, when the country moved to level 2 and finally level 1 (September 2021). The recruitment has been severely interrupted particularly between 26 March and 30 July 2020. Upon resuming community recruitment, additional Covid-19 specific questions were added to the questionnaire, support and surveillance protocols were implemented and a dedicated COVID-19 nurse was appointed. Intervention face-to-face interactions were replaced with virtual face-to-face sessions during more severe lockdowns and micronutrient supplements were delivered to participants' homes. To boost trial recruitment, we launched a revised community drive that included community pop-up stands, and enlisted community leaders aiding recruitment drives. We have also developed a participant retention standard operating procedures. In conclusion, changing the course of the obesity tide globally will require a major concerted effort to examine initiatives across the lifecourse. The preconception period, in particular, represents an exciting and largely untested stage in life to prevent the transgenerational impacts of maternal metabolic and mental health.

Acknowledgements
The authors extend our gratitude to the South African Medical Research Council and the Canadian Institutes of Health Research for funding *Bukhali*. We greatly appreciate the willingness of all women who have agreed to participate in the study and the staff who are implementing this ambitious research programme. We also acknowledge the role of WHO in supporting the HeLTI collaboration and providing quality assurance support to *Bukhali.* We thank the HeLTI PI Research Committee and HeLTI Office for their intellectual contributions, comradery and administrative support.

Author Contributions
SAN and SL are co-principal investigators for Healthy Life Trajectory Initiative South Africa. SAN, CD, LW, AP and SL wrote the initial protocol draft. CMS, CLD, PA, DGB, ZAB, LB, BC, TC, BF, CG, JH, JJ, HJ, JMJ, KK, AK, EL, NL, MCM, MR, DER, SWS, DS, WS, DMS, MS, ST, MT, ST, SM, and LMR read and contributed to the final version. All authors provided edits and critiqued the manuscript for intellectual content.

Funding
The authors extend our gratitude to the South African Medical Research Council (Strategic Health Innovation Partnerships grant) and the Canadian Institutes of Health Research for funding (grant number: HLS 151553) *Bukhali.*

Trial Sponsor
University of the Witwatersrand, Johannesburg, South Africa; Contact Dr Drennan (robin.drennan@wits.ac.za).

Competing interests
We have no conflicts of interest to declare. The HeLTI Council of funders and WHO require all Principal Investigators to complete annual Declarations of Interest and to commit to having no direct or indirect relations with the tobacco, arms and food industries during the course of the trial.

Patient consent for publication Not required.

For peer review only

Provenance and peer review Not commissioned; peer-reviewed for ethical and funding approval prior to submission.

References

1. Wang H, Naghavi M, Allen C, *et al*. Global, regional, and national life expectancy, all-cause mortality, and cause-specific mortality for 249 causes of death, 1980-2015: a systematic analysis for the global burden of disease study 2015. *Lancet* 2016;388:1459–544.
2. World Health Organisation. https://www.who.int/news-room/spotlight/10-global-health-issues-to-track-in-2021 Accessed November 2021
3. Wells JC, Sawaya AL, Wibaek R, *et al*. The double burden of malnutrition: aetiological pathways and consequences for health. *Lancet* 2020;395:75-88.
4. World Obesity Federation. *Atlas of Childhood Obesity.* 2019, London.
5. Nagpal TS, Souza SCS, Moffat M, *et al*. Does prepregnancy weight change have an effect on subsequent pregnancy health outcomes? A systematic review and meta-analysis. *Obes Rev* 2021; e13324.
6. Woo Baidal JA, Locks LM, Cheng ER, *et al*. Risk factors for childhood obesity in the first 1,000 days: a systematic review. *Am J Prev Med* 2016;50:761–79
7. Heslehurst N, Vieira R, Akhter Z, *et al*. The association between maternal body mass index and child obesity: A systematic review and meta-analysis. *PLoS Med* 2019;16:e1002817.
8. Dean SV, Lassi ZS, Imam AM, *et al*. Preconception care: nutritional risks and interventions. *Reprod Health* 2014;11:S3.
9. Dean SV, Lassi ZS, Imam AM, *et al*. Preconception care: closing the gap in the continuum of care to accelerate improvements in maternal, newborn and child health. *Reprod Health* 2014;11:S1
10. Nyati LH, Pettifor JM, Norris SA. The prevalence of malnutrition and growth percentiles for urban South African children. *BMC Public Health* 2019;19:492.
11. Lundeen EA, Norris SA, Adair LS, *et al*. Sex differences in obesity incidence: 20-year prospective cohort in South Africa. *Pediatr Obes*. 2016;11:75-80.
12. Macaulay S, Ngobeni M, Dunger DB, Norris SA. The prevalence of gestational diabetes mellitus amongst black South African women is a public health concern. *Diabetes Res Clin Pract*. 2018;139:278-287
13. Prioreschi A, Wrottesley SV, Said-Mohamed R, *et al*. Understanding how maternal social and biological factors are related to fetal growth in an urban South African cohort. *J Dev Orig Health Dis.* 2021;12:79-87.
14. Barker M, Dombrowski SU, Colbourn T, *et al*. Intervention strategies to improve nutrition and health behaviours before conception. *Lancet*. 2018; 391:1853-1864.
15. Dennis CL, Marini F, Dick JA, *et al*. Protocol for a randomised trial evaluating a preconception-early childhood telephone-based intervention with tailored e-health resources for women and their partners to optimise growth and development among children in Canada: a Healthy Life Trajectory Initiative (HeLTI Canada). *BMJ Open*. 2021;11:e046311.
16. Wu Y, Marc I, Bouchard L, *et al*. Study protocol for the Sino-Canadian Healthy Life Trajectories Initiative (SCHeLTI): a multicentre, cluster-randomised, parallel-group, superiority trial of a multifaceted community-family-mother-child intervention to prevent childhood overweight and obesity. *BMJ Open*. 2021;11:e045192.
17. Kumaran K, Krishnaveni GV, Suryanarayana KG, *et al*. Protocol for a cluster randomised trial evaluating a multifaceted intervention starting preconceptionally-Early Interventions to Support Trajectories for Healthy Life in India (EINSTEIN): a Healthy Life Trajectories Initiative (HeLTI) Study. *BMJ Open*. 2021;11:e045862.
18. Cohen et al. Material and relational difficulties: The impact of the household environment on the emotional wellbeing of young black women living in Soweto, South Africa. . *Family Issues* 2020, Vol. 41(8) 1307–1332
19. Draper CE, Bosire E, Prioreschi A, *et al*. Urban young women's preferences for intervention strategies to promote physical and mental health preconception: A Healthy Life Trajectories Initiative (HeLTI). *Prev Med Rep* 2019;14:100846

20. Ware LJ, Prioreschi A, Bosire E, *et al*. Environmental, Social, and Structural Constraints for Health Behavior: Perceptions of Young Urban Black Women During the Preconception Period – A Healthy Life Trajectories Initiative. *J Nutr Educ Behav*. 2019; S1499-4046:30178-2
21. Bosire EN et al. Young women's perceptions of life in urban South Africa: Contextualising the preconception knowledge gap. African *J Reproduc Health*. 2021;25:39-49.
22. Prioreschi A, Wrottesley SV, Slemming W, *et al*. A qualitative study reporting maternal perceptions of the importance of play for healthy growth and development in the first two years of life. *BMC Pediatr* 2020 Sep 9;20:428
23. Wrottesley SV, Prioreschi A, Slemming W, *et al*. Maternal perspectives on infant feeding practices in Soweto, South Africa. Public Health Nutr. 2021; 24:3602-3614
24. Silubonde TM, Baumgartner J, Ware LJ, *et al*. Adjusting Haemoglobin Values for Altitude Maximizes Combined Sensitivity and Specificity to Detect Iron Deficiency among Women of Reproductive Age in Johannesburg, South Africa. *Nutrients*. 2020;12:633
25. Ware LJ, Kim AW, Prioreschi A, Nyati LH, Taljaard W, Draper CE, Lye SJ, Norris SA. Social vulnerability, parity and food insecurity in urban South African young women: the healthy life trajectories initiative (HeLTI) study. *J Public Health Policy*. 2021;42:373-389.
26. Prioreschi A, Wrottesley SV, Norris SA. Physical Activity Levels, Food Insecurity and Dietary Behaviours in Women from Soweto, South Africa. J Community Health. 2020 Jun 19
27. Kehoe S, Wrottesley S, Prioreschi A, *et al*. Food insecurity, diet quality and body composition: data from the Healthy Life Trajectories Initiative pilot survey in urban Soweto, South Africa. *Public Health Nutr*. 2021;24:1629-1637
28. Wrottesley SV, Prioreschi A, Kehoe SH, Ward KA, Norris SA. A maternal "mixed, high sugar" dietary pattern is associated with fetal growth. *Matern Child Nutr* 2019;16: e12912
29. Prioreschi A, Wrottesley SV, Said-Mohamed R, *et al*. Understanding how maternal social and biological factors are related to fetal growth in an urban South African cohort. *J Dev Orig Health Dis*. 2020 Feb 17:1-9
30. Wrottesley SV, Prioreschi A, Norris SA. The association between fetal growth and neonatal adiposity in urban South African infants. *Pediatr Obes*. 2021;16:e12737
31. Soepnel L et al. Obesity and adiposity of 3- to 6-year-old children born to mothers with hyperglycemia detected in pregnancy in an urban South African setting. Annals Human Biol. 2020 48:81-92
32. Draper CE, Prioreschi A, Ware LJ, *et al*. Pilot implementation of *Bukhali*: A preconception health trial in South Africa. *SAGE Open Med*. 2020 Jul 7;8
33. Michie S, Richardson M, Johnston M, *et al*. The behavior change technique taxonomy (v1) of 93 hierarchically clustered techniques: building an international consensus for the reporting of behavior change interventions. *Ann Behav Med* 2013;46:81-95.
34. Lawrence W, Black C, Tinati T, *et al*. 'Making every contact count': Evaluation of the impact of an intervention to train health and social care practitioners in skills to support health behaviour change. *J Health Psychol*. 2016;21:138-51.
35. Lawrence W, Vogel C, Strömmer S, *et al*. How can we best use opportunities provided by routine maternity care to engage women in improving their diets and health? *Matern Child Nutr* 2020;16:e12900.
36. Adam LM, Jarman M, Barker M *et al*. Use of healthy conversation skills to promote healthy diets, physical activity and gestational weight gain: Results from a pilot randomised controlled trial. *Patient Educ Couns* 2020;103:1134-1142.
37. MRC guidance on process evaluation of complex interventions, (https://www.mrc.ac.uk/documents/pdf/mrc-phsrn-process-evaluation-guidance-final/).
38. PA Harris, R Taylor, R Thielke, *et al*, Research electronic data capture (REDCap) – A metadata-driven methodology and workflow process for providing translational research informatics support, *J Biomed Inform 2009;42:377-81*
39. Gaye A, Marcon Y, Isaeva J, *et al*. DataSHIELD: taking the analysis to the data, not the data to the analysis, *Int J Epi* 2014; 43:1929–1944

Legends

Figure 1. Participant flow diagram

Figure 2. Pathways to impact model that informed the intervention development

Figure 3. Impact of outcomes in a multi-phase trial and intervention components

For peer review only

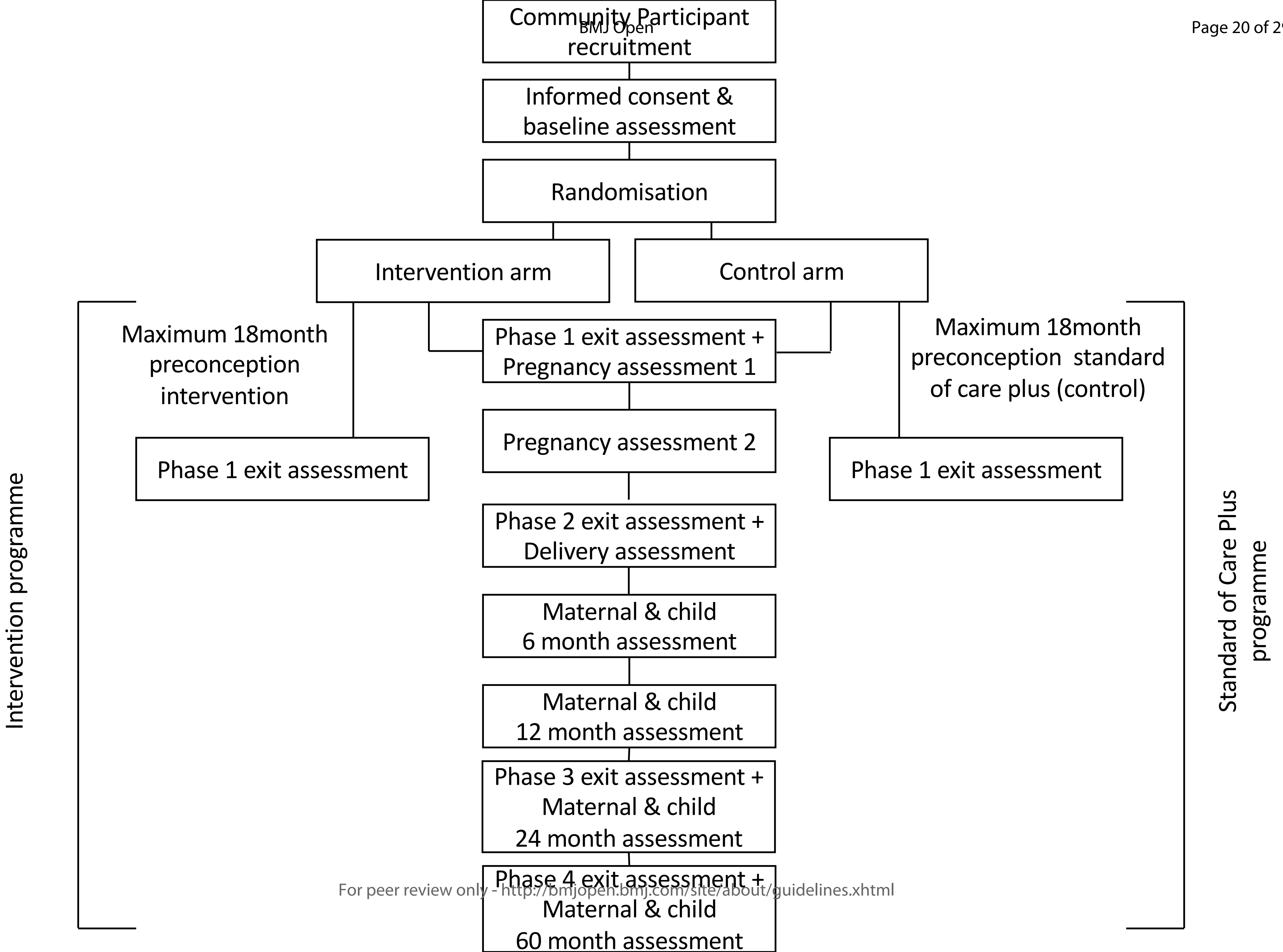
Community Participant recruitment
Informed consent & baseline assessment
Randomisation
Intervention arm
Control arm
Maximum 18month preconception intervention
Phase 1 exit assessment
Phase 1 exit assessment + Pregnancy assessment 1
Pregnancy assessment 2
Phase 2 exit assessment + Delivery assessment
Maternal & child 6 month assessment
Maternal & child 12 month assessment
Phase 3 exit assessment + Maternal & child 24 month assessment
Phase 4 exit assessment + Maternal & child 60 month assessment
Maximum 18month preconception standard of care plus (control)
Phase 1 exit assessment
Intervention programme
Standard of Care Plus programme

Logic model for programme of intervention to improve physical and mental health in women

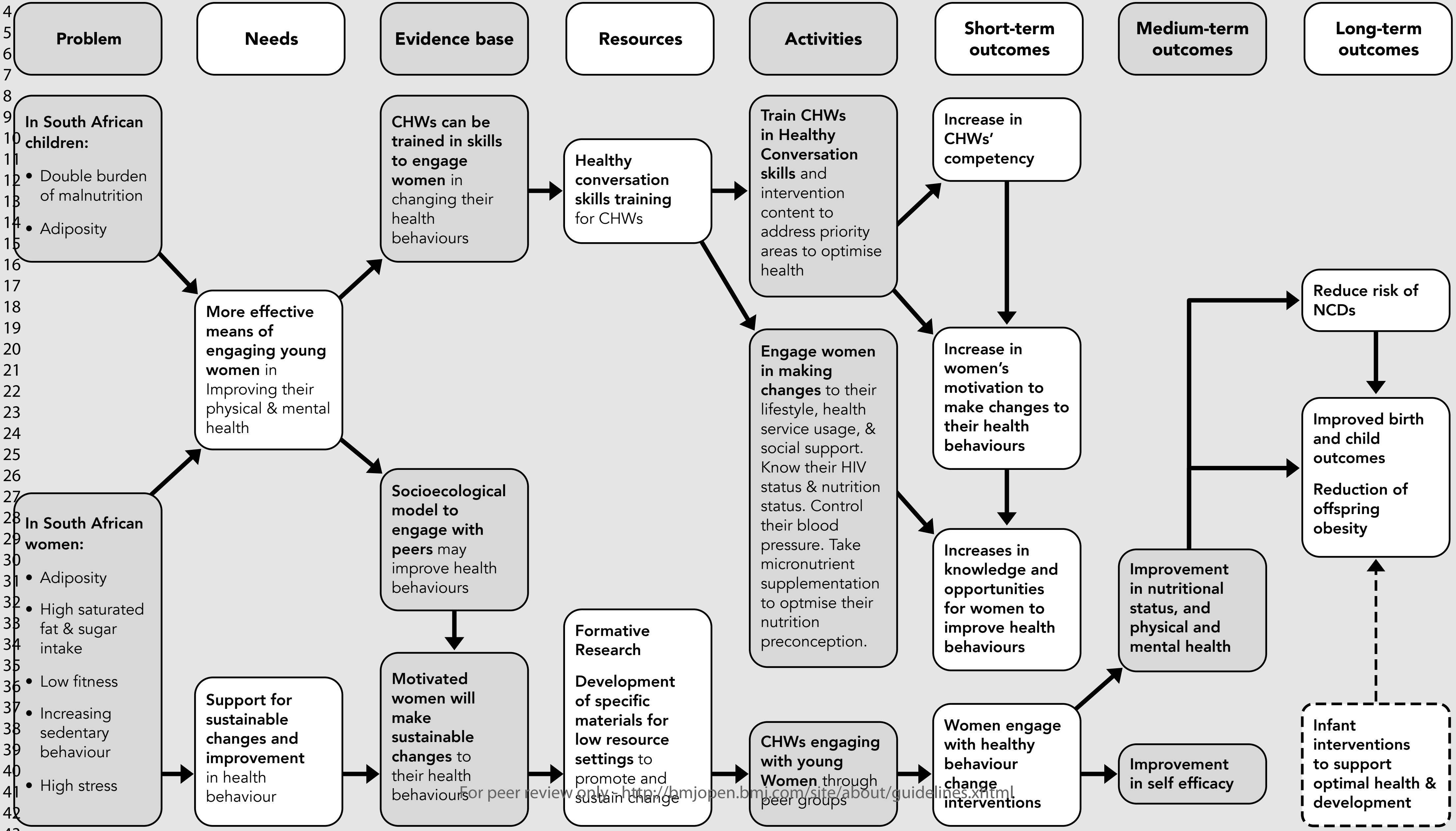

For peer review only - http://bmjopen.bmj.com/site/about/guidelines.xhtml

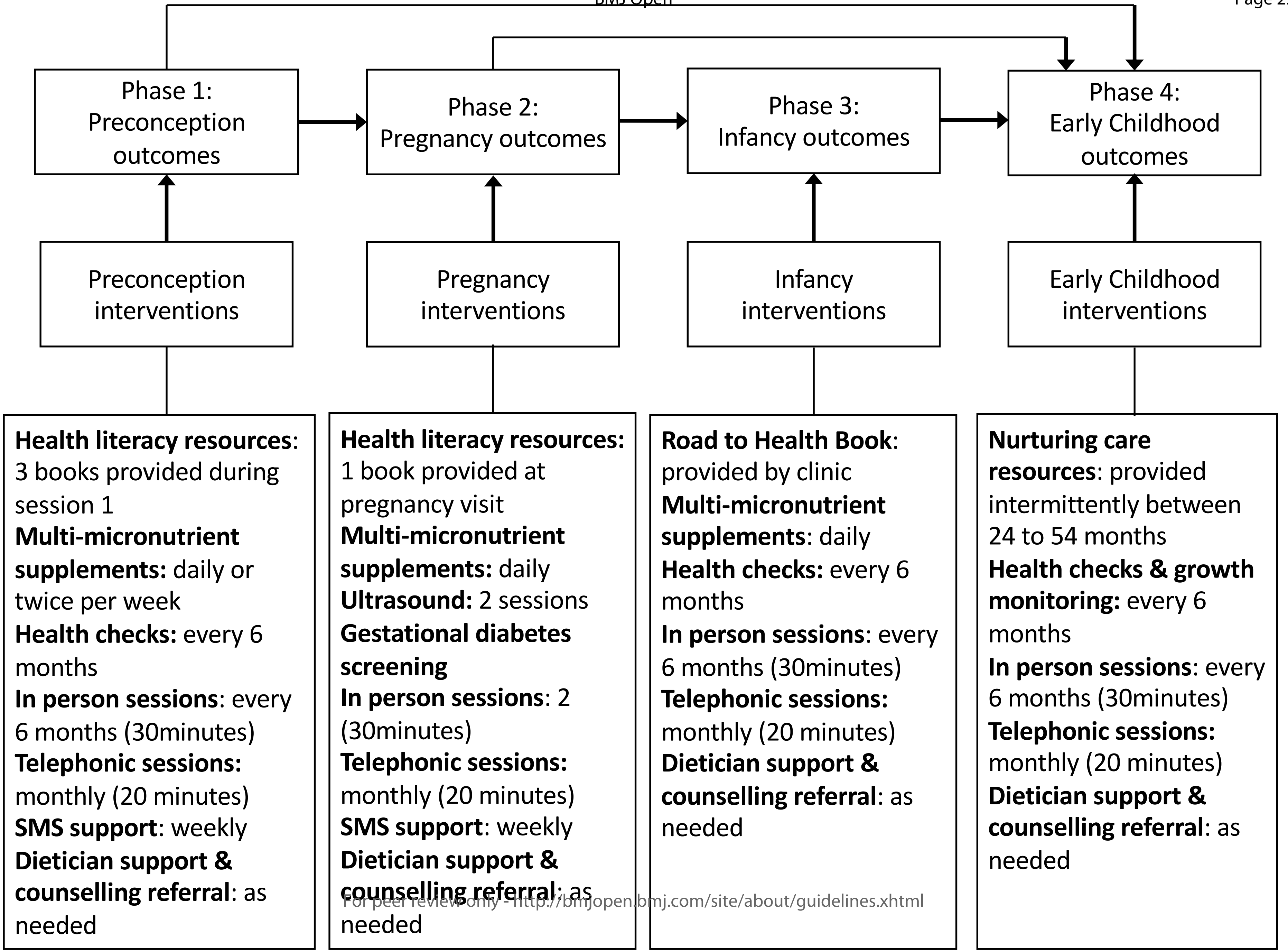

For peer review only - http://bmjopen.bmj.com/site/about/guidelines.xhtml

Table 1: World Health Organization Trial Registration Data Set

Data category	Information
Primary registry and trial identifying number	PACTR201903750173871
Date of registration in primary registry	25 March 2019
Source(s) of monetary or material support	South African Medical Research Council; Canadian Institutes of Health Research
Primary sponsor	University of the Witwatersrand, Johannesburg, South Africa
Contact for public queries	SAN; shane.norris@wits.ac.za
Contact for scientific queries	SAN; shane.norris@wits.ac.za
Public title	HeLTI-SA: a preconception randomised control trial
Countries of recruitment	South Africa
Health condition(s) or problem(s) studied	Child obesity and non-communicable disease risk
Intervention(s)	Active comparator: community health worker, health literacy, behaviour change, dietetic and counselling support; risk-screening health services; multi-micronutrient supplement<br>Placebo comparator: standard of care plus non-health related call centre support
Key inclusion and exclusion criteria	Women aged 18-28 years are eligible, unless they are: (i) diagnosed with type-I diabetes or epilepsy because these require intensive treatment and management priorities; (ii) present with intellectual disability that hinders informed consent, and (iii), not able or willing to provide consent.
Study type	Complex prevention intervention; individual randomisation; research team blind to randomisation; phase II
Date of first enrolment	October 2019
Target sample size	6800 women
Recruitment status	Recruiting
Primary outcome(s)	DXA-derived fat mass index (fat mass/height^2)
Key secondary outcomes	Maternal and child anthropometry, cardiometabolic, and behaviour, and child developmental outcomes

For peer review only

Reporting checklist for protocol of a clinical trial.

Based on the SPIRIT guidelines.

Instructions to authors

Complete this checklist by entering the page numbers from your manuscript where readers will find each of the items listed below.

Your article may not currently address all the items on the checklist. Please modify your text to include the missing information. If you are certain that an item does not apply, please write "n/a" and provide a short explanation.

Upload your completed checklist as an extra file when you submit to a journal.

In your methods section, say that you used the SPIRITreporting guidelines, and cite them as:

Chan A-W, Tetzlaff JM, Gøtzsche PC, Altman DG, Mann H, Berlin J, Dickersin K, Hróbjartsson A, Schulz KF, Parulekar WR, Krleža-Jerić K, Laupacis A, Moher D. SPIRIT 2013 Explanation and Elaboration: Guidance for protocols of clinical trials. BMJ. 2013;346:e7586

		Reporting Item	Page Number
**Administrative information**			
Title	#1	Descriptive title identifying the study design, population, interventions, and, if applicable, trial acronym	1
Trial registration	#2a	Trial identifier and registry name. If not yet registered, name of intended registry	3
Trial registration: data set	#2b	All items from the World Health Organization Trial Registration Data Set	Supp table
Protocol version	#3	Date and version identifier	3
Funding	#4	Sources and types of financial, material, and other support	16
Roles and responsibilities:	#5a	Names, affiliations, and roles of protocol contributors	1; 16

For peer review only

contributorship			
Roles and responsibilities: sponsor contact information	#5b	Name and contact information for the trial sponsor	16
Roles and responsibilities: sponsor and funder	#5c	Role of study sponsor and funders, if any, in study design; collection, management, analysis, and interpretation of data; writing of the report; and the decision to submit the report for publication, including whether they will have ultimate authority over any of these activities	16
Roles and responsibilities: committees	#5d	Composition, roles, and responsibilities of the coordinating centre, steering committee, endpoint adjudication committee, data management team, and other individuals or groups overseeing the trial, if applicable (see Item 21a for data monitoring committee)	14
**Introduction**			
Background and rationale	#6a	Description of research question and justification for undertaking the trial, including summary of relevant studies (published and unpublished) examining benefits and harms for each intervention	4
Background and rationale: choice of comparators	#6b	Explanation for choice of comparators	8
Objectives	#7	Specific objectives or hypotheses	4
Trial design	#8	Description of trial design including type of trial (eg, parallel group, crossover, factorial, single group), allocation ratio, and framework (eg, superiority, equivalence, non-inferiority, exploratory)	6
**Methods: Participants, interventions, and**			

For peer review only

**outcomes**			
Study setting	#9	Description of study settings (eg, community clinic, academic hospital) and list of countries where data will be collected. Reference to where list of study sites can be obtained	4
Eligibility criteria	#10	Inclusion and exclusion criteria for participants. If applicable, eligibility criteria for study centres and individuals who will perform the interventions (eg, surgeons, psychotherapists)	7
Interventions: description	#11a	Interventions for each group with sufficient detail to allow replication, including how and when they will be administered	8-10
Interventions: modifications	#11b	Criteria for discontinuing or modifying allocated interventions for a given trial participant (eg, drug dose change in response to harms, participant request, or improving / worsening disease)	6
Interventions: adherance	#11c	Strategies to improve adherence to intervention protocols, and any procedures for monitoring adherence (eg, drug tablet return; laboratory tests)	14
Interventions: concomitant care	#11d	Relevant concomitant care and interventions that are permitted or prohibited during the trial	N/A
Outcomes	#12	Primary, secondary, and other outcomes, including the specific measurement variable (eg, systolic blood pressure), analysis metric (eg, change from baseline, final value, time to event), method of aggregation (eg, median, proportion), and time point for each outcome. Explanation of the clinical relevance of chosen efficacy and harm outcomes is strongly recommended	7;12
Participant timeline	#13	Time schedule of enrolment, interventions (including any run-ins and washouts), assessments, and visits for participants. A schematic diagram is highly recommended (see Figure)	8

For peer review only

Sample size	#14	Estimated number of participants needed to achieve study objectives and how it was determined, including clinical and statistical assumptions supporting any sample size calculations	7
Recruitment	#15	Strategies for achieving adequate participant enrolment to reach target sample size	7
**Methods: Assignment of interventions (for controlled trials)**			
Allocation: sequence generation	#16a	Method of generating the allocation sequence (eg, computer-generated random numbers), and list of any factors for stratification. To reduce predictability of a random sequence, details of any planned restriction (eg, blocking) should be provided in a separate document that is unavailable to those who enrol participants or assign interventions	6
Allocation concealment mechanism	#16b	Mechanism of implementing the allocation sequence (eg, central telephone; sequentially numbered, opaque, sealed envelopes), describing any steps to conceal the sequence until interventions are assigned	6
Allocation: implementation	#16c	Who will generate the allocation sequence, who will enrol participants, and who will assign participants to interventions	6
Blinding (masking)	#17a	Who will be blinded after assignment to interventions (eg, trial participants, care providers, outcome assessors, data analysts), and how	6
Blinding (masking): emergency unblinding	#17b	If blinded, circumstances under which unblinding is permissible, and procedure for revealing a participant's allocated intervention during the trial	14
**Methods: Data collection,**			

For peer review only

**management, and analysis**			
Data collection plan	#18a	Plans for assessment and collection of outcome, baseline, and other trial data, including any related processes to promote data quality (eg, duplicate measurements, training of assessors) and a description of study instruments (eg, questionnaires, laboratory tests) along with their reliability and validity, if known. Reference to where data collection forms can be found, if not in the protocol	10-12
Data collection plan: retention	#18b	Plans to promote participant retention and complete follow-up, including list of any outcome data to be collected for participants who discontinue or deviate from intervention protocols	16
Data management	#19	Plans for data entry, coding, security, and storage, including any related processes to promote data quality (eg, double data entry; range checks for data values). Reference to where details of data management procedures can be found, if not in the protocol	10
Statistics: outcomes	#20a	Statistical methods for analysing primary and secondary outcomes. Reference to where other details of the statistical analysis plan can be found, if not in the protocol	11
Statistics: additional analyses	#20b	Methods for any additional analyses (eg, subgroup and adjusted analyses)	11
Statistics: analysis population and missing data	#20c	Definition of analysis population relating to protocol non-adherence (eg, as randomised analysis), and any statistical methods to handle missing data (eg, multiple imputation)	11
**Methods: Monitoring**			
Data monitoring: formal committee	#21a	Composition of data monitoring committee (DMC); summary of its role and reporting structure;	14

For peer review only

		statement of whether it is independent from the sponsor and competing interests; and reference to where further details about its charter can be found, if not in the protocol. Alternatively, an explanation of why a DMC is not needed	
Data monitoring: interim analysis	#21b	Description of any interim analyses and stopping guidelines, including who will have access to these interim results and make the final decision to terminate the trial	11
Harms	#22	Plans for collecting, assessing, reporting, and managing solicited and spontaneously reported adverse events and other unintended effects of trial interventions or trial conduct	14
Auditing	#23	Frequency and procedures for auditing trial conduct, if any, and whether the process will be independent from investigators and the sponsor	14
**Ethics and dissemination**			
Research ethics approval	#24	Plans for seeking research ethics committee / institutional review board (REC / IRB) approval	3
Protocol amendments	#25	Plans for communicating important protocol modifications (eg, changes to eligibility criteria, outcomes, analyses) to relevant parties (eg, investigators, REC / IRBs, trial participants, trial registries, journals, regulators)	3
Consent or assent	#26a	Who will obtain informed consent or assent from potential trial participants or authorised surrogates, and how (see Item 32)	3
Consent or assent: ancillary studies	#26b	Additional consent provisions for collection and use of participant data and biological specimens in ancillary studies, if applicable	10
Confidentiality	#27	How personal information about potential and enrolled participants will be collected, shared, and	10

For peer review only

		maintained in order to protect confidentiality before, during, and after the trial	
Declaration of interests	#28	Financial and other competing interests for principal investigators for the overall trial and each study site	13
Data access	#29	Statement of who will have access to the final trial dataset, and disclosure of contractual agreements that limit such access for investigators	16
Ancillary and post trial care	#30	Provisions, if any, for ancillary and post-trial care, and for compensation to those who suffer harm from trial participation	N/A
Dissemination policy: trial results	#31a	Plans for investigators and sponsor to communicate trial results to participants, healthcare professionals, the public, and other relevant groups (eg, via publication, reporting in results databases, or other data sharing arrangements), including any publication restrictions	6;14
Dissemination policy: authorship	#31b	Authorship eligibility guidelines and any intended use of professional writers	N/A
Dissemination policy: reproducible research	#31c	Plans, if any, for granting public access to the full protocol, participant-level dataset, and statistical code	14
**Appendices**			
Informed consent materials	#32	Model consent form and other related documentation given to participants and authorised surrogates	Supplementary document
Biological specimens	#33	Plans for collection, laboratory evaluation, and storage of biological specimens for genetic or molecular analysis in the current trial and for future use in ancillary studies, if applicable	Table 3

The SPIRIT Explanation and Elaboration paper is distributed under the terms of the Creative Commons Attribution License CC-BY-NC. This checklist was completed on 04. December 2021 using https://www.goodreports.org/, a tool made by the EQUATOR Network in collaboration with Penelope.ai

For peer review only

BMJ Open

Building knowledge, optimising physical and mental health, and setting up healthier life trajectories in South African women (Bukhali): a preconception randomised control trial part of the Healthy Life Trajectories Initiative (HeLTI)

Journal:	*BMJ Open*
Manuscript ID	bmjopen-2021-059914.R2
Article Type:	Protocol
Date Submitted by the Author:	22-Mar-2022

Complete List of Authors:	Norris, Shane; University of the Witwatersrand, SAMRC Developmental Pathways for Health Research Unit, Department of Paediatrics; University of Southampton, Global Health Research Institute, School of Health and Human Development<br>Draper, Catherine; University of the Witwatersrand, SAMRC Developmental Pathways for Health Research Unit<br>Prioreschi, Alessandra; University of the Witwatersrand, MRC/Wits Developmental Pathways for Health Research Unit<br>Smuts, CM; North-West University<br>Ware, Lisa; University of the Witwatersrand, Department of Paediatrics<br>Dennis, CindyLee; University of Toronto<br>Awadalla, Philip; University of Toronto, Department of Molecular Genetics, Ontario Institute for Cancer Research<br>Bassani, D; SickKids Research Institute, Centre for Global Child Health<br>Bhutta, Zulfiqar; SickKids Research Institute, Centre for Global Child Health<br>Briollais, Laurent; Lunenfeld-Tanenbaum Research Institute,<br>Cameron, D; Ottawa Hospital General Campus, Medicine, Division of Infectious Diseases; Ottawa Hospital Research Institute<br>Chirwa, Tobias; University of the Witwatersrand<br>Fallon, B; University of Toronto<br>Gray, CM; University of Stellenbosch, Division of Molecular Biology and Human Genetics; University of Cape Town, Division of Immunology, Institute of Infectious Disease and Molecular Medicine<br>Hamilton , Jill; University of Toronto, Department of Paediatrics<br>Jamison, J; Red River College<br>Jaspan, Heather; University of Cape Town, Division of Immunology, Institute of Infectious Disease and Molecular Medicine<br>Jenkins, Jennifer; University of Toronto, Department of Applied Psychology and Human Development<br>Kahn, Kathleen; University of the Witwatersrand<br>Kengne , AP; South African Medical Research Council<br>Lambert, Estelle; University of Cape Town, Division of Exercise Science and Sports Medicine<br>Levitt, Naomi; University of Cape Town<br>Martin , Marie-Claude ; University of Toronto<br>Ramsay, Michele; University of the Witwatersrand,<br>Roth, Daniel; The Hospital for Sick Children; University of Toronto,

	Department of Paediatrics<br>Scherer, Stephen; University of Toronto, Department of Molecular Genetics; The Hospital for Sick Children<br>Sellen, Daniel; University of Toronto; University of Toronto, Department of Nutritional Sciences<br>Slemming, Wiedaad; University of the Witwatersrand, Department of Paediatrics<br>Sloboda, Deborah; Hamilton, Department of Biochemistry and Biomedical Sciences<br>Szyf, M.; McGill University, Department of Pharmacology and Therapeutics<br>Tollman, Stephen; University of the Witwatersrand<br>Tomlinson, Mark; University of Stellenbosch, Institute for Life Course Health Research; Queen's University Belfast<br>Tough, Suzanne ; Alberta Children's Hospital Research Institute, Department of Paediatrics<br>Matthews, Stephen; University of Toronto, Department of Applied Psychology and Human Development; University of Toronto<br>Richter, Linda; University of the Witwatersrand, DSI-NRF Centre of Excellence in Human Development<br>Lye, Stephen; University of Toronto, Department of Applied Psychology and Human Development; University of Toronto
**Primary Subject Heading**:	Public health
Secondary Subject Heading:	Nutrition and metabolism
Keywords:	NUTRITION & DIETETICS, PUBLIC HEALTH, SOCIAL MEDICINE

SCHOLARONE™
Manuscripts

Building knowledge, optimising physical and mental health, and setting up healthier life trajectories in South African women (Bukhali): a preconception randomised control trial part of the Healthy Life Trajectories Initiative (HeLTI)

Norris SA[1], Draper CE[1], Prioireschi A[1], Smuts CM[2], , Ware L[1,28], Dennis CL[3], Awadalla P[4], Bassani DG[5], Bhutta ZA[5,6], Briollais L[7,19], Cameron B[8], Chirwa T[9], Fallon B[10], Gray CM[11,30], Hamilton J[12], Jamison J[13], Jaspan H[11], Jenkins JM[14], Kahn K[15], Kenge AP[16], Lambert E[17], Levitt N[18], Martin MC[19], Ramsay M[20], Roth, DE[5,12], Scherer SW[4,21], Sellen D[7,22], Slemming W[23], Sloboda DM[24], Szyf M[25], Tollman S[15], Tomlinson M[26,31], Tough S[27], Matthews S[14,19,28], Richter LM[29], Lye S[14,19,28]

Affiliations

1. SAMRC Developmental Pathways for Health Research Unit, Department of Paediatrics, Faculty of Health Sciences, University of the Witwatersrand, Johannesburg, South Africa
2. Centre of Excellence for Nutrition, North-West University, Potchefstroom, South Africa
3. Lawrence S. Bloomberg Faculty of Nursing, University of Toronto, Toronto, Canada
4. Department of Molecular Genetics, Ontario Institute for Cancer Research, University of Toronto, Toronto, Canada
5. Centre for Global Child Health, The Hospital for Sick Children, Toronto, Canada
6. Institute for Global Health and Development, Aga Khan University, Karachi, Pakistan
7. Dalla Lana School of Public Health, University of Toronto, Toronto, Canada
8. Ottawa Hospital Research Institute, University of Ottawa, Ottawa, Canada
9. School of Public Health, University of the Witwatersrand, Johannesburg, South Africa
10. Factor-Inwentash Faculty of Social Work, University of Toronto, Toronto, Canada
11. Division of Immunology, Institute of Infectious Disease and Molecular Medicine, Faculty of Health Sciences, University of Cape Town, Cape Town, South Africa
12. Department of Paediatrics, The Hospital for Sick Children, University of Toronto, Toronto, Canada
13. School of Health Sciences and Community Services, Red River College, Winnipeg, Canada
14. Department of Applied Psychology and Human Development, University of Toronto, Toronto, Canada
15. SAMRC Rural Public Health and Health Transitions Research Unit, School of Public Health, University of the Witwatersrand, Johannesburg, South Africa
16. Non-communicable Diseases Research Unit, South African Medical Research Council, Cape Town, South Africa
17. Division of Exercise Science and Sports Medicine, University of Cape Town, Cape Town, South Africa
18. Chronic Disease Initiative for Africa, University of Cape Town, Cape Town, South Africa
19. Lunenfeld-Tanenbaum Research Institute, Sinai Health System, University of Toronto, Toronto, Canada
20. Sydney Brenner Institute for Molecular Bioscience, University of the Witwatersrand, Johannesburg, South Africa
21. The Centre for Applied Genomics, The Hospital for Sick Children, Toronto, Canada; Program in Genetics and Genome Biology, The Hospital for Sick Children, Toronto, Canada; McLaughlin Centre, University of Toronto, Toronto, Canada
22. Department of Nutritional Sciences, University of Toronto, Toronto, Canada; Joannah and Brian Lawson Centre for Child Nutrition, University of Toronto, Toronto, Canada
23. Department of Paediatrics, University of the Witwatersrand, Johannesburg, South Africa
24. Department of Biochemistry and Biomedical Sciences, McMaster University, Hamilton, Canada
25. Department of Pharmacology and Therapeutics, McGill University, Montreal, Canada
26. Institute for Life Course Health Research, Department of Global Health, Stellenbosch University, Stellenbosch, South Africa
27. Department of Paediatrics, Alberta Children's Hospital Research Institute, Calgary, Canada
28. Department of Physiology, University of Toronto, Toronto, Canada; Departments of Obstetrics and Gynaecology and Medicine, University of Toronto, Toronto, Canada
29. DSI-NRF Centre of Excellence in Human Development, University of the Witwatersrand, Johannesburg, South Africa

For peer review only

30. Division of Molecular Biology and Human Genetics, Stellenbosch University, Cape Town, South Africa
31. School of Nursing and Midwifery, Queens University, Belfast, UK

Corresponding author: Professor Shane Norris; shane.norris@wits.ac.za

Email addresses:
Draper CE: Catherine.Draper@wits.ac.za
Smuts CM: Marius.Smuts@nwu.ac.za
Prioireschi A: Alessandra.Prioreschi@wits.ac.za
Ware L: lisa.ware@wits.ac.za
Dennis CL: cindylee.dennis@utoronto.ca
Awadalla P: Philip.Awadalla@oicr.on.ca
Bassani DG: diego.bassani@sickkids.ca
Bhutta ZA: zulfiqar.bhutta@sickkids.ca
Briollais L: laurent@lunenfeld.ca
Cameron B: BCAMERON@toh.ca
Chirwa T: Tobias.Chirwa@wits.ac.za
Fallon B: barbara.fallon@utoronto.ca
Gray CM: cgray@sun.ac.za
Hamilton J: jill.hamilton@sickkids.ca
Jamison J: janetijamieson@gmail.com
Jaspan H: hbjaspan@gmail.com
Jenkins JM: jenny.jenkins@utoronto.ca
Kahn K: Kathleen.Kahn@wits.ac.za
Kenge AP: Andre.Kengne@mrc.ac.za
Lambert E: vicki.lambert@uct.ac.za
Levitt N: Naomi.Levitt@uct.ac.za
Martin MC: mcmartin@lunenfeld.ca
Ramsay M: Michele.Ramsay@wits.ac.za
Roth, DE: daniel.roth@sickkids.ca
Scherer SW: stephen.scherer@sickkids.ca
Sellen D: dan.sellen@utoronto.ca
Slemming W: Wiedaad.Slemming@wits.ac.za
Sloboda DM: sloboda@mcmaster.ca
Szyf M: moshe.szyf@mcgill.ca
Tollman S: Stephen.Tollman@wits.ac.za
Tomlinson M: markt@sun.ac.za
Tough S: stough@ucalgary.ca
Matthews S: stephen.matthews@utoronto.ca
Richter LM: Linda.Richter@wits.ac.za
Lye S: LYE@lunenfeld.ca

For peer review only

Article summary

Introduction: South Africa's evolving burden of disease is challenging due to a persistent infectious disease, burgeoning obesity, most notably among women, and rising rates of non-communicable diseases (NCDs). With two thirds of women presenting at their first antenatal visit either overweight or obese in urban South Africa (SA), the preconception period is an opportunity to optimise health and offset transgenerational risk of both obesity and NCDs.

Methods and analysis: *Bukhali* is the first individual randomised controlled trial in Africa to test the efficacy of a complex continuum of care intervention and forms part of the Healthy Life Trajectories Initiative (HeLTI) consortium implementing harmonised trials in Canada, China, India and SA. Starting preconception and continuing through pregnancy, infancy and childhood, the intervention is designed to improve nutrition, physical and mental health, and health behaviours of South African women to offset obesity-risk (adiposity) in their offspring. Women aged 18-28 years (n=6800) will be recruited from Soweto, an urban-poor area of Johannesburg. The primary outcome is dual-energy x-ray absorptiometry derived fat mass index (fat mass divided by height^2) in the offspring at age 5-years. Community health workers will deliver the intervention randomly to half the cohort by providing health literacy material, dispensing a multi-micronutrient supplement, providing health services and feedback, and facilitating behaviour change support sessions to optimise: (i) nutrition, (ii) physical and mental health, and (iii) lay the foundations for healthier pregnancies and early child development.

Ethics and dissemination: Ethical approval has been obtained from the Human Ethics Research Committee University of the Witwatersrand, Johannesburg, South Africa (M1811111), the University of Toronto, Canada (19-0066-E) and the WHO Ethics Committee (ERC.0003328). Data and biological sample sharing policies are consistent with the governance policy of the HeLTI Consortium (https://helti.org) and South African government legislation (POPIA). The recruitment and research team will obtain informed consent.

Strengths and limitations of the study

- *Bukhali* will be the first RCT in Africa to evaluate if a community health worker facilitated intervention starting preconception will reduce offspring obesity risk at age 5-years.
- Primary and many secondary outcomes are harmonised with three other HeLTI trial-sites (Canada, China and India) to enable comparative and pooled analyses.
- The impact of *Bukhali's* data and learning will be to inform preconception prevention of NCD-risk strategies in Africa.
- A limitation is that *Bukhali* does not include an economic evaluation.

Trial registration: this trial is registered with the Pan African Clinical Trials Registry (https://pactr.samrc.ac.za) on 25 March 2019 (identifier: PACTR201903750173871). See Supplementary Table 1

Protocol version: 20 March 2022 (version #4). Any protocol amendments will be communicated to investigators, IRBs, trial participants, and trial registries.

Keywords: pre-conception, South Africa, childhood obesity, early child development, healthy trajectories

Background

Non-communicable diseases (NCDs) such as obesity, hypertension, and diabetes are significant health burdens in many rapidly transitioning middle-income countries[1]. NCDs kill 41 million people each year (71% of deaths) globally, and WHO has identified NCDs as one of the ten leading threats to health[2]. From 1980 to 2014, the prevalence of diabetes changed little in western Europe, but doubled in sub-Saharan Africa (SSA), India and China[1]. SSA countries have persisting afflictions from infectious diseases (HIV, TB and malaria) against a backdrop of maternal and child malnutrition[3], but also now the COVID-19 pandemic. In many respects the impetus to address NCDs as part of the Sustainable Development Goals has stalled as a result of the COVID-19 emergency.

Childhood weight gain, overweight and obesity, and adiposity are risk factors for poor health trajectories and development of NCDs. The number of children and adolescents with obesity has risen to 124 million worldwide and could exceed 250 million by 2030 without substantial interventions[4]. A meta-analysis of 61 studies showed that increased preconception and interpregnancy weight gain was associated with gestational diabetes (88% greater risk), preeclampsia (92% greater risk), and large-for-gestational-age (36% greater risk) babies[5]. A systematic review of 38 studies found a consistent association between maternal pre-pregnancy weight and offspring obesity[6]. Furthermore, Heslehurst and colleagues showed that a child has a 2.6-fold greater risk of obesity if the mother is obese prior to conception[7]. A systematic review by Dean and co-authors identified 23 randomised controlled trials of preconception care and counselling delivered via health facilities or community groups, that reported improved women's health behaviours (calorie restriction and increased physical activity), as well as, more positive maternal, neonatal and child health outcomes including an increase in antenatal care seeking and an increased likelihood of breast feeding[8,9]. None of these preconception interventions were conducted in Africa.

South Africa's (SA) evolving burden of disease is complex due to: (i) persisting malnutrition; (ii) the highest prevalence of overweight and obesity in SSA, particularly among women; and (iii) rising rates of non-communicable diseases such as of type 2 diabetes (T2D). In Soweto (urban-poor area of the Johannesburg Metropole covering 200 km^2 with over 1.3 million residents; 6400/km^2) we have found that the prevalence of overweight and obesity in females increases from 10% by age 8 to 43% by age 22 years, while the prevalence in males remains fairly constant into young adulthood at 10%; further, that girls who are obese by age 5 are 42 times more likely to be obese adults[10,11]. Furthermore, 66% of pregnant Soweto women at first antenatal clinic visit, were either overweight or obese, and 10% were diagnosed with gestational diabetes (GDM) in late pregnancy[12,13]. Consequently, there is a high obesity burden need in Soweto for preconception interventions to support women to optimise health and manage weight to combat intergenerational obesity[14].

The Healthy Life Trajectory Initiative (HeLTI), an international consortium developed in partnership with the WHO to address childhood obesity. We hypothesise that an integrated complex intervention comprising a continuum of care starting pre-conception and across pregnancy, infancy and childhood will reduce childhood adiposity and the risk for NCDs, as well as, improve child development. Four randomised controlled trials in Ontario and Alberta (Canada)[15], Shanghai (China)[16], Mysore (India)[17], and Soweto (South Africa) have been harmonised to test this hypothesis. These sites were selected based on the community need and that they possessed both the infrastructure, and multidisciplinary teams with cohort/trial experience to execute an ambitious ten-year programme of work. This paper describes the HeLTI South African trial protocol in Soweto called *Bukhali* (a Zulu word meaning smart/powerful) signifying the beneficial potential of optimising women's health for herself and her future offspring.

Methods/design

Patient and public involvement

We constituted a Community Advisory Group with local women, and have involved them in the intervention development and the preparatory phase to enable feedback and input into the feasibility of the study. Furthermore, a Participant Advisory Group has been formed as part of a longitudinal qualitative study to further understand and identify preconception health concerns, barriers and solutions, as well as, other contextual issues that participants are facing which could influence their

participation in the trial. Lastly, a Stakeholder Advisory Group has been formed with multiple stakeholders (South African government departments of health and education, UNICEF, Save the Children, WHO, NGOs, and academics) to share the development and results of *Bukhali* and elicit input and guidance around a variety of issues – policy relevance, linkage with current initiatives, ethics, and dissemination.

Formative research and pilot trial study

The trial was designed following the Standard Protocol Items (Recommendations for Interventional Trials (SPIRIT) 2013 statement). Through extensive qualitative (focus groups, in-depth individual interviews, and stakeholder engagement)[18-23] and epidemiological research[24-31], we identified our young women (18-28 years) as the target group given that a high proportion these women are already either overweight or obese and most will have their first child during this age range, and developed the intervention package. We initially opted for a cluster randomised study design in order to included community peer group sessions as part of the intervention. To assess the feasibility and implementation of this design, a pilot trial of data collection and intervention commenced in 2018 and concluded in 2019. We recruited 1719 participants within six community clusters, randomised the clusters, collected baseline data and biological samples, and implemented the planned intervention and control arm activities. The pilot trial data highlighted critical learning, among others, that: (i) it was feasible to recruit and collect good quality data and biological samples (95% consented to provide a blood sample and 75% consented to provide a DNA sample) from a large group of our target population; (ii) young women living in Soweto lead complicated and economically stressful lives and are pre-occupied with survival strategies, which often entails moving and living at multiple homes across family and friends in the community over time; (iii) there is poor health literacy around preconception health amongst young women in Soweto; (iv) young women were anxious about participation due to fear of criminal activities and abduction; (v) community safety of study community health workers could not be assured; and (vi) we could not effectively deliver the planned community peer group component due to poor attendance despite convenient Saturday meeting times, multiple opportunities during the month to attend, and the provision of refreshments. To address these challenges, we formulated a multi-pronged strategy: (i) we rolled-out a communication campaign; a soap opera series of 11 episodes drawing attention to preconception health issues in multiple languages through two local radio stations with the largest population reach supported by social media engagement (https://soundcloud.com/user-662329775); (ii) we engaged and obtained additional support to operate within the community through community leadership forums and police awareness of the study team; (iii) we produced branded material for staff and vehicles with clear university and project identification to promote credibility; and (iv) we modified the study design (we pivoted from cluster-randomisation to individual-randomisation due to cluster contamination linked to young women shifting between multiple households) and intervention delivery, and jettisoned the peer group component because of poor compliance[32].

Study design

Bukhali is an individual-randomised controlled two-arm trial, (simple randomisation; 1:1 ratio to the intervention arm or to standard of care plus control arm). After informed consent and baseline data collection, the participant moves to a separate/private randomisation counter where the participant clicks on a computer-generated electronic envelope to reveal the arm allocation. The randomisation officer then escorts the participant to a separate wing on a different floor within the centre to introduce participant to either the intervention arm or control arm coordinator, thus preserving the concealment of allocation. The research trial data team and associated researchers will be blinded to participant allocation throughout the trial, and this will be achieved by: (i) ensuring that group allocation is locked on the database and cannot be accessed by the research team; (ii) training the research team not to ask and encouraging participants not to divulge their allocation status; and (iii) when participants visit the centre for their respective arm programme these activities will occur in a separate part of the centre away from the research team. All participants will be exposed to the preconception intervention or control programme for up to 18 months. If a participant does not become pregnant within the 18 months, she will have an exit assessment. If a participant becomes pregnant, she will exit the preconception phase and be monitored through pregnancy, and postnatally. An 'index child' conceived after randomisation will be followed until age 5-years. Participant flow through the trial is seen in Figure 1.

For peer review only

Insert Figure.1

Inclusion/exclusion criteria

Participants will be recruited through a survey of Soweto households (approximately 30000), which is sufficiently large to adequately recruit our target participant number. Women aged 18-28 years are eligible, unless they are: (i) diagnosed with type-I diabetes or epilepsy because these require intensive treatment and management priorities; (ii) present with intellectual disability that hinders informed consent, and (iii), not able or willing to provide consent. Pregnancy at the time of the baseline assessment does not exclude participation. Participants who move residence during the course of the study will continue participation as long as their current address is known, the participant wishes to remain involved, and data can be collected. Participants who have miscarriages will be offered counselling support and remain in the study.

Sample size

The sample size calculation is based on the mean (4.1 kg/m^2) and SD (2.1) for dual-energy x-ray absorptiometry (DXA) derived fat mass index (FMI) calculated from our pre-pubertal data from the Birth to Twenty Plus Cohort in Soweto, South Africa. We will require 765 children to be born in each arm to detect a meaningful difference of 0.25 SD (0.5 kg/m^2) in FMI at age 5-years between the intervention and control groups at 80% power and 5% significance level, and assuming 30% attrition. Therefore, we will recruit approximately 6800 women so reach a target of 1530 pregnancies. The intervention group will receive all four phases of the intervention and the control remains standard of care plus throughout. Pooling of data across the four countries will provide the ability to detect an effect size of 0.1 SD between intervention and control groups at 80% power and 5% significance level. The pooled analysis should also allow us to detect a 0.2 SD difference in our primary outcome in boys and girls separately (although this is dependent on the direction of change being the same across our populations).

Primary and secondary outcome questions

Primary child outcome: What is the relative effect of a 4-phase intervention on adiposity status at age 5-years as determined by DXA-derived fat mass index (fat mass/$height^2$) among children of young adult women in the intervention arm, when compared to the children of women who receive standard of care plus (control arm)?

Secondary child outcomes: What are the effects of the 4-phase intervention on child outcomes at age 5-years on:

1. Anthropometry (age- and sex-standardized continuous BMI-z score, BMI growth trajectories, continuous waist circumference z- score, upper arm circumference, head circumference);
2. Cardiometabolic outcomes (systolic and diastolic blood pressure, lipid profile, glucose, insulin, insulin resistance);
3. Behaviour (dietary intake, physical activity, screen time, sedentary behaviour, sleep time);
4. Developmental outcomes (cognitive, motor skills, communication, behavioural); and

Secondary maternal outcomes: What are the effects of the 4-phase intervention on?

1. Anthropometry (overweight and obesity (OWO), DXA-derived body composition, waist circumference);
2. Nutrition (anaemia)
3. Physical health (hypertension);
4. Mental health (depressive symptoms, anxiety) and
5. Behaviour (dietary patterns, physical activity, screen time, sedentary behaviour, sleep time, tobacco and alcohol use);

Phase-specific outcomes

1. Preconception Phase (outcome at conception or exit): What is the effect of the preconception intervention phase on: maternal anthropometry and body composition (overweight and obesity, fat mass index, waist circumference); nutrition (anaemia); health behaviour (physical activity, screen time, sleep); mental health (depressive symptoms, anxiety); and physical health (hypertension)?

For peer review only

2. Pregnancy Phase (parental and child outcomes at birth): What is the effect of the preconception + pregnancy intervention phases on: pregnancy weight gain; rate of gestational diabetes; rate of gestational hypertension; rate of pre-eclampsia; rate of preterm birth < 37 weeks; rates of large- and small- birthweight for gestational age; and maternal mental health?
3. Infancy Phase (parental and child outcomes across the first two years postpartum): What is the effect of the preconception + pregnancy + infancy intervention phases on: breastfeeding outcomes (initiation, duration, exclusivity, self-efficacy); nutrition (anaemia), health behaviour outcomes (physical activity, screen time, sleep); and mental health outcomes (depressive symptoms, anxiety), and child outcomes (growth, blood pressure, sleep, sedentary and physical activity; feeding practices; developmental outcomes)?
4. Early Childhood phase (parental and child outcomes 2-5 years postpartum): What is the effect of the preconception + pregnancy + infancy +childhood intervention phases on: maternal nutrition (anaemia), health behaviour outcomes (physical activity, screen time, sleep); and mental health outcomes (depressive symptoms, anxiety); and child outcomes (growth, blood pressure, sleep, sedentary and physical activity; dietary practices; cognitive development outcomes)?

Ethics and regulatory management

Bukhali has ethical approval from the Human Ethics Research Committee of the University of the Witwatersrand, Johannesburg, South Africa (M1811111), the University of Toronto, Canada (19-0066-E) and the WHO Ethics Committee (ERC.0003328). The trial is registered with the Pan African Clinical Trial Registry.

Intervention: community health worker approach

The intervention has been informed by the UK MRC Guidelines for Complex Interventions, and we used the TIDieR (Template for Intervention Description and Replication) Checklist. Using the taxonomy of behaviour change techniques (BCTs)[33], we grounded the behaviour change intervention in: (i) theory of planned behaviour, which suggests that behaviours are determined by attitudes, subjective norms and perceived control; (ii) control theory, which suggests we use inner- and outer-control to minimise deviation from the standard; and (iii) social cognitive theory, which posits behaviour and choices are influenced by the environment, personality and other factors such as goals. These theories combined anchors the intervention to value the importance of context, individual needs, and support to bolster agency. Figure 2 details the original logic model to illustrate the pathways to impact that guided the intervention development. The intervention is centred on “Living your best life” with four universal objectives that infuse through all phases of the intervention (preconception, pregnancy, infancy and childhood) to: (i) optimise health; (ii) optimise nutrition; (iii) optimise mental health; and (iv) promote early childhood development. The agents of change are modelled upon community health workers (CHWs), who we call “Health Helpers” (HHs) to differentiate them from current Department of Health workers. We opted for the CHW approach as there is positive efficacy evidence in Africa, and it aligns with SA’s primary health care re-engineering policy. The HHs will share similar qualifications, characteristics and salary levels to CHWs in SA, and eligibility includes: women between the ages of 23-40 years with completed secondary school with some tertiary education, but no formal training as a CHW is required.

The HHs are supported by a supervisor and dietician with access to nurses and a referral network. HHs deliver the intervention through the provision of health literacy resource materials and micronutrient supplements, provide health feedback and services, and they support behaviour change through individual sessions in person or telephonically, and supportive text messaging. Multiple strategies will be put in place by the HHs (flexibility, reminders, home visits, incentives) to aid in achieving trial compliance.

Insert Figure 2.

Healthy Conversation Skills and trauma-informed care: HHs will be trained in Healthy Conversation Skills (HCS), a technique specifically developed and tested for use with socioeconomically disadvantaged participants to improve self-efficacy and support behaviour change. HCS is based on a social-cognitive model of health behaviour, which emphasises the role of increasing self-efficacy in promoting behaviour change[34-36]. It recognises that knowledge alone is insufficient to

change behaviour unless people are also motivated and empowered to change. Trained in HCS, HHs will explore the barriers that that women experience in trying to change their behaviour, ways of over-coming these barriers and setting goals for change. The HCS approach enables a woman to reflect on her particular priorities for physical and mental health, and to support her in finding her own solutions to challenges she experiences. The training is standardised and supported by the MRC Lifecourse Epidemiology Unit (UK). HCS training is aimed at achieving four basic competencies that are known to be useful in supporting behaviour change: (i) the use of open-discovery questions, questions that begin with 'how' and 'what' and lead people to explore and find their own ways of overcoming barriers to change; (ii) listening more than talking which allows people to identify and take control of their own behaviour change; (iii) reflecting on practice in order to be more effective; and (iv) supporting the use of SMARTER goal setting so that participants have a sense of change and progress. To the HCS approach we have added selected skills from the WHO Problem Management Plus (individual psychological help for adults impaired by distress in communities exposed to adversity).

Health literacy resource material: With topic experts and a specialised curriculum developer, we integrated behaviour change theory and designed both HH facilitator manuals and participant resource materials. The resource material encompasses 9 modules (women's health, chronic diseases, diet, sleep, physical activity and fitness, sitting time, body size and image, emotional awareness, and caregiving) in 6 chapters, and these modules repeat with new content every 6 chapters. The resource books contain essential educational content, local narratives to convey key concepts, and space for participants to engage with the material through the use of checklists, diaries and reflections. The approach for each module is that of: (i) Knowing (what do I know about it?); (ii) Doing (what am I doing about it?); and (iii) Becoming (how do I live my best life?). Three preconception health resource books are provided; one book is provided for pregnancy health, and supplementary resource materials to the South African Road to Health Booklet (https://sidebyside.co.za/resources/road-to-health-book/) and nurturing care resources are provided for infancy and early childhood.

Multi-micronutrient supplementation: based on 6-monthy point-of-care haemoglobin concentration assessments, the HHs will dispense and monitor micronutrient supplement use. The MMN supplement is based on WHO recommendations (Table 1) to optimise nutrition and address anaemia, based on haemoglobin concentrations:

- Non-anaemic women (Hb ≥12 g/dL at enrolment): Provision of a micronutrient supplement containing approximately 27 mg iron twice (2x) per week.
- Mildly anaemic women (Hb <12 g/dL and >7g/dL at enrolment): Provision of a micronutrient supplement daily for six months.
- Severely anaemic women (Hb <7g/dL at baseline): Receive treatment regime according to current standard of care in South Africa.

Supplement use will continue throughout the preconception period until pregnancy or exit. If the participant becomes pregnant, the supplement regimen will then shift to daily use throughout pregnancy and 6-months post-partum.

Table 1: Nutrient composition of multiple micronutrient supplements

Micronutrient	Unit	Nutrient Reference Value (NRV) EU
Vitamin A	2664 IU	100% (800 µg / 2664 IU)
Vitamin D3	200 IU	100% (5 µg / 200 IU)
Tocopherol (Vitamin E)	15 mg	125% (12 mg / 17.9 I
Vitamin K1	55 µg	73% (75 µg)
Thiamine	1.4 mg	127% (1.1 mg)
Riboflavin	1.4 mg	100% (1.4 mg)
Niacinamide	18 mg	113% (16 mg)

For peer review only - http://bmjopen.bmj.com/site/about/guidelines.xhtml

Pantothenic acid	6 mg	100% (6 mg)
Pyridoxine (Vitamin B6)	1.9 mg	136% (1.4 mg)
Biotin	30 µg	60% (50 µg)
Folic acid	600 µg	300% (200 µg)
Cyanocobalamin (Vitamin B12)	2.6 µg	100% (2.5 µg)
Ascorbic acid	60 mg	75% (80mg)
Copper	1.15 mg	100% (1 mg)
Iodine	250 µg	167% (150 µg)
Iron	27 mg	193% (14 mg)
Selenium	50 µg	100% (55 µg)
Zinc	10 mg	100% (10 mg)

Services: Through individual in-person or telephonic sessions, HHs will provide: (i) specific health feedback to participants based on their baseline results (BMI (obesity), blood pressure (hypertension), haemoglobin concentration (anaemia), mental health, and lifestyle (physical activity, sedentary behaviour, sleep); (ii) offer home-based HIV and pregnancy testing with counselling, utilise HCS to identify aspects of health behaviour change, set goals and monitor, and provide access to healthcare referral networks. For overweight and obese women, additional waist circumference measurements will be provided. During the infant and child periods, HHs continue the monthly consultations and support women with aid of the South African Road to Health Booklet and postnatal clinic visits.

Control arm: call centre approach (standard of care plus)
In addition to access to standard of care offered through the public health system, control arm participants will have a dedicated call centre. The rationale to add a standard of care plus programme is to maintain contact with participants, and minimise attrition bias. Similar to the intervention team, the call centre team will receive training in the delivery of the modules. To partially control for special attention given to women in the intervention arm, the call centre will contact participants once a month and deliver a curriculum on "life skills" via telephone, SMS and email. The non-health specific intervention material will be standardised and the modules of the curriculum will include: (i) civic rights and responsibilities (How do I apply for an identity document; what does it mean to vote?); (ii) financial education (savings & budgets; how do I open a bank account); (iii) job readiness (preparing a CV; job interview skills; volunteering); (iv) navigating social media (internet, different social media; how social media can be harmful), and (v) social grants. HIV and pregnancy testing services will be offered on request for free at the research centre.

Process evaluation
For all intervention (health worker-centric) and control (call centre-centric) components, Standard Operating Procedures (SOPs) support the HHs and call centre team to deliver their respective modules in a structured and uniform way. We will follow the MRC guidance on process evaluation of complex interventions[37]. Evaluation will focus on implementation, mechanisms of impact and context. Assessment of implementation will focus on finding out what is delivered and how delivery is achieved, and which is vital information if the intervention is to be replicated. We will assess compliance by counts using a log (for example, contacts made), and by discussions between HHs and participants. We will measure fidelity of intervention delivery by observing HHs during one-to-one contacts with intervention group participants, focusing on HHs use of HCS and the competencies they demonstrate. We will also observe the contact between call centre assistants and control participants to record the interactions. We will monitor intervention dose by recording the frequency and duration of contact between participants and HHs using diaries. To understand the mechanisms through which the intervention brings about change in the trial's specified outcomes, we will assess the acceptability of the intervention, and women's experiences of contact with HHs using interviews and focus groups. In assessing the context, we will aim to identify factors that might act as barriers or facilitators to intervention implementation or effects. This will include local and national policy, local

For peer review only

service configuration and provision, and socio-demographic and environmental factors within the communities taking part in the study. Qualitative methods (interviews and focus groups), including longitudinal approaches, will be used to further explore specific issues of relevance, in terms of participants' experiences of the trial, key issues facing participants, and factors influencing implementation of the trial.

Data collection, management and analysis

Table 2 and 3 details the phenotype measures and biological sample collection on women, biological fathers and index child. Formative research has indicated low-level (10%) biological father participation in the study. A dedicated research team will be established increase father participation through alternate approaches (for example: data collection flexibility in evening and on Saturdays; encouraging fathers to accompany pregnant women for their ultrasound scan). There is substantial harmonisation of data, samples and quality management across the four HeLTI studies. For example, over 1800 core variables have been harmonized from preconception through to infancy (6 months). All data will be managed through real-time capturing, reporting and quality control utilising REDCap electronic data capture tools[38]. Maelstrom Research at McGill University (Canada), has been supporting the harmonisation process across the sites and developing the online catalogue and metadata, which are available on their website (https://www.maelstrom-research.org/study/helti-hp). In addition, Maelstrom will support the implementation of a data infrastructure within each country using DataSHIELD/Opal software[39] to allow federated data analysis (country-specific or meta-analysis by pooling estimates across countries) to be conducted without having access to individual participant data or exporting and pooling data on a central server. This capacity will provide a mechanism for addressing restrictive regulation regarding exporting data across countries. The data management system coupled with our laboratory management system of samples (different tissues at different time points) will enable researchers to conduct future mechanistic studies and participants have provided consent for this on provision of IRB approval for such studies. All personal data are managed in a separate and secure with restricted access in line with the Protection of Personal Information Act of South Africa.

Analysis plan

Standard data screening/cleaning procedures will be conducted including the extent and pattern of missing data. When data are missing at random (MAR), which we expect to be the case, we will utilise multiple imputation so as to maximise sample size and study power in a dataset. We will assess the comparability at randomisation of the groups using descriptive analyses. We will also assess process indicators including recruitment and retention, and indicators of programme delivery in the Intervention Group. Sensitivity analyses will be performed to assess the influence of attrition and compliance. Intention-to-treat analysis will be used for key outcomes based on all viable pregnancies adjusted for compliance and selection of viable pregnancies to minimise bias. For the primary outcome, we will assess the effect of the intervention on fat mass index at age 5-years. The concept of this multiple-phase trial, is that the outcomes of interest in a particular phase result from the intervention administered in the same phase, but is also mediated by the outcomes observed in the previous phase(s). For example, a reduction in obesity risks during pregnancy caused by the intervention in this phase will impact birth weight and the child weight/BMI observed in the next phase. Therefore, at the end of the study, we will measure an "overall" effect of the interventions over the four phases on the final outcomes, but also, could assess how the association between the final outcomes and the interventions is mediated by the phase-specific outcomes (see Figure 3). Other secondary anthropometric outcomes to be assessed at 5-years of age include OWO, BMI and centiles, and adiposity (skin-fold thickness, arm circumference). We will also assess the effect of the intervention on glucose metabolism, blood pressure and cognitive outcomes. Mixed models with random intercept/slope effects and fixed effects (for the intervention) will be used to estimate the intervention effect. Due to possible heterogeneity of effects for key outcomes according to the time of initiation of the intervention (e.g. preconception versus pregnancy) we will conduct a subgroup analysis stratifying by this variable, testing for heterogeneity. The distribution of modifiable risk factors for childhood obesity, poor cardiometabolic and neurodevelopmental outcomes at baseline, and their changes over time will be assessed according to study group.

Insert Figure 3.

Table 2. Longitudinal data collection measures (M-mother, F-Father, C-Infant/child)

Domain	Measure	Preconception		Pregnancy (week)		Postnatal period (months)				
Time point (months)		Baseline	18 month (exit)	10-17	24-28	Delivery	6	12	24	60
Demography	Demographic and socio-economic data, household composition, occupation, education, living environment	M	M	M	F		M	M	M	M
	Date of birth / sex	M			F	C				
Anthropometry and body composition	Height/length	M			F	C	C	C	C	C
	Weight	M	M	M	M/F	C	M/C	M/C	M/C	M/C
	Gestational weight gain			M	M					
	Body Mass Index	M	M	M	M/F		M	M	M	M
	Adiposity: DXA (Adult)	M	M		F		M		M/C	M/C
	Skinfolds (triceps & subscapular) (*if scan is not possible)			M	M		M*		M*	M*
	Peapod body composition					C	C			
	Waist circumference	M	M		F		C	M/C	M/C	M/C
	Fetal anthropometry (gestational age and fetal growth)			C	C	C (GA)				
	Mid upper arm circumference	M	M	M	M/F	C	C	C	M/C	M/C
	Head circumference					C	C	C	C	C
Clinical	Pregnancy Complications			M	M					
	Birth complications, survival					M/C	C	C	C	C
	APGAR score 1 & 5 mins					C				
	HIV test			M	F					M
	Pregnancy test (pre-DXA scan)	M	M				M		M	M
	Blood pressure	M	M	M	M/F		M	M	M/C	M/C
	Anaemia (HemoCue)	M	M	M	M/F			M	M	M
	HbA1c	M	M	M	M/F			M	M	M
	Random blood glucose	M	M	M	F					
	Fasting blood glucose				M			M	M/C	M/C
	Oral glucose tolerance test				M					M/C
	Plasma & Serum collection	M	M	M	M/F			M	M/C	M/C
	DNA collection	M	M	M	M/F			M		M/C
	Urine spot sample	M	M		M/F				C	M/C
	Urine dipstick	M	M	M	M/F				C	M/C
	Heel-prick blood								C	
	Buccal swab								C	

**Lifestyle and health behaviour**	Diet intake (Dietary diversity & Household Food Insecurity Access Scale)	M	M	M	M/F			M	M	M
	Breastfeeding Questionnaire					M				
	Feeding (breastfeeding, solid food introduction, diversity, eating behaviour; SUNRISE Questionnaire)						C	C	C	C
	Physical activity (M:GPAQ & Sitting Time Quesstionnaire)	M	M	M	M/F			C/M	**M**	M
	Step Test		M		F					
	Accelerometry (sub-study)								C	C
	Screen Time Questionnaire	M	M	M	M/F		C	C	C	C
	Sleep: Pittsburgh Sleep Quality Index	M	M	M	M/F		M	M	M	M
	Brief Infant Sleep Questionnaire						C	C	C	
	SUNRISE sleep questionnaire									C
	Tobacco, alcohol, and drug use: Exposure questionnaire (WHO-STEPS, AUDIT-Questionnaire)	M	M	M	M/F			M	M	M
	Environmental tobacco exposure				M			C	C	C
	Child Care Questionnaire						M	M	M	M
**Mental and physical health, and chid development**	Depression (PHQ9)	M	M	M	M/F			M		M
	Depression (Edinburgh)			M	M		M			
	Stress (PSS)			M	M/F		M		M	M
	Stressful life events (ACEs)	M			F					
	Generalised Self-Efficacy Scale	M	M	M	M/F			M		M
	Social Support Questionnaire	M	M	M	M/F					
	Social Provisions Scale				M					M
	Medical history (and TB), family history, drug, supplement history	M	M	M	M/F		M	M	M	M
	Breasfeeding efficacy				M					
	Anxiety (GAD-7)	M	M	M	M/F			M	M	M
	Medication/supplement use	M	M	M	M/F	M/C	M/C	M/C	M/C	M/C
	Medical events (hospitalisation)				M	M/C	M/C	M/C	M/C	M/C
	Births, pregnancy & reproductive health	M		M		M	M	M	M	M
	Vaccinations						C	C	C	C
	Emotional health Questionnaire	M	M	M	M/F					
	Ages and Stages questionnaire						C	C	C	C
	Parenting practices (Brigance Parent-Child Interaction Scale)							M	M	

	Neurodevelopment (WHO Global Scale for Early Development - short-form)							C	C	
	Executive function (Early Years Toolbox)									C
	Temperament/behaviour (Strengths and Difficulties Questionnaire)								C	C
	School readiness (International Development and Early Learning Assessment)									C
	Stimulation in the home (UNICEF Multiple Inidcators Cluster Survey Early Childhood Development, Home Learning Environment)								C	C

Governance and monitoring

The shared governance and harmonisation structures for the HeLTI trials include: Research Committee of country Principal Investigators; a single Data Monitoring Committee that is independent from the sponsor and competing interests (DMC Charter is available on the HeLTI website); WHO Monitoring and Evaluation Team; and a HeLTI Secretariat. The trial is blinded to the investigators and research data collection team, but the data management team will unblind participant's allocation upon request by the DMC or ethics committee for key reporting or in cases of risk. A governance framework has been developed and adopted by all partners that details the management and access of data and biological samples. The HeLTI website (https://helti-net.org) provides more information including contact details of the HeLTI office and secretariat and information on the Data Access Portal through which investigators from around the world can view and request access to data/biospecimens from individual HeLTI country datasets. HeLTI will also be registered as a hub on Global Health Network (https://tghn.org) The Research Committee is supported by seven cross-consortium active working groups, including: (i) Core Variable Harmonization, (ii) Interventions and Retention, (iii) Early Child Development Measures, (iv) Biospecimen Collection and Management, (v) Data Management and Quality Assurance, (vi) Capacity Building, and (vii) Publications. Results will actively be disseminated within the four countries and through WHO mechanisms. An independent clinical research associate will audit the trial annually. WHO aims to regularly review quality assurance data of the trial. An electronic management system is in place for trial HHs and staff to record and manage adverse events. Utilising REDCap, Adverse Event Forms will be completed online, which will trigger a process of alerts and recommendations from the investigators, researchers and project coordinators to best handle the event. All events are also discussed at the HH weekly debrief session and reported to the DMC.

For peer review only

Table 3. Longitudinal biological sample measures

	Preconception		Pregnancy (weeks)		Postnatal period (months)		
	Baseline	18 month	10-17	24-28	12	24	60
**Mother**							
**Blood collection**	X (random)	X (random)	X (random)	X (fasting; OGTT)	X (fasting)	X (fasting)	X (fasting; OGTT)
**Plasma**	X	X	X	X	X	X	X
**Serum**	X	X	X	X	X	X	X
**DNA**	X		X	X	X		X
**Urine (spot sample)**	X	X		X			X
**Urine (dipstick)**	X	X	X	X			X
**Father**							
**Blood - random**				X			
**Plasma**				X			
**Serum**				X			
**DNA**				X			
**Urine (spot sample)**				X			
**Urine (dipstick)**				X			
**Infant/Child**							
**Blood collection (DBS)**					X		
**Blood collection**						X (fasting)	X (Fasting; OGTT)
**Plasma**						X	X
**Serum**						X	X
**DNA**							X
**Buccal swab**						X	
**Urine (spot sample & dipstick)**						X	X

Trial status and impact of the Covid-19 pandemic

Trial formative research started in April 2017, the pilot trial was initiated in 2018, main trial recruitment began in October 2019 and is expected to conclude early in 2022, and the trial is expected to be completed by 2029. The collective HeLTI initiative provides an unprecedented preconception health platform with the potential to expand to other settings. To maximise the learning and impact of these studies, HeLTI aims to host postgraduate students and training opportunities, and facilitate accelerated managed access to data and biological samples. Our formative work has revealed just how complex both the environments and lives of young urban women in South Africa are. The health implications are further compounded by poorer than anticipated health literacy levels. Among these women, health is not prioritised amidst competing economic and food security concerns, and the daily stress of navigating challenging household (relationships, households with multi-families) and community circumstances (poverty, crime and exposure to violence). Also, the Soweto environment is becoming more obesogenic with easier access to less healthy fast foods that are affordable and sold on most street corners. This makes the decision to purchase healthier, more expensive foods that are sold further away less appealing[20].

For peer review only - http://bmjopen.bmj.com/site/about/guidelines.xhtml

For these reasons, we have opted for a more pragmatic and adaptive trial design, whereby the intervention package can be modified to the circumstances of each participant and can incorporate new learning on how best to engage, support and improve compliance. The strength of this approach is to remain relevant and responsive to the urban poor context, and thereby, increase the likelihood of achieving health behaviour change. The challenge of this adaptive and individually-responsive approach is how to capture and analyse the dose effect of intervention elements. To address this challenge, we have setup a sophisticated data management and dashboard system which can monitor key quality assurance, delivery and compliance indicators that are not only useful for trial management, but also assist the intervention and control arm teams.

Six months into the main trial the Covid-19 pandemic hit South Africa through a series of waves and lockdown levels. A hard lockdown (level 5) was implemented in South Africa from 26 March - 30 April 2020. Thereafter a level 4 lockdown was implemented until 31 May 2020. Level 3 then lasted until 17 August, when the country moved to level 2 and finally level 1 (September 2021). The recruitment has been severely interrupted particularly between 26 March and 30 July 2020. Upon resuming community recruitment, additional Covid-19 specific questions were added to the questionnaire, support and surveillance protocols were implemented and a dedicated COVID-19 nurse was appointed. Intervention face-to-face interactions were replaced with virtual face-to-face sessions during more severe lockdowns and micronutrient supplements were delivered to participants' homes. To boost trial recruitment, we launched a revised community drive that included community pop-up stands, and enlisted community leaders aiding recruitment drives. We have also developed a participant retention standard operating procedures. In conclusion, changing the course of the obesity tide globally will require a major concerted effort to examine initiatives across the lifecourse. The preconception period, in particular, represents an exciting and largely untested stage in life to prevent the transgenerational impacts of maternal metabolic and mental health.

Acknowledgements
The authors extend our gratitude to the South African Medical Research Council and the Canadian Institutes of Health Research for funding *Bukhali*. We greatly appreciate the willingness of all women who have agreed to participate in the study and the staff who are implementing this ambitious research programme. We also acknowledge the role of WHO in supporting the HeLTI collaboration and providing quality assurance support to *Bukhali.* We thank the HeLTI PI Research Committee and HeLTI Office for their intellectual contributions, comradery and administrative support.

Author Contributions
SAN and SL are co-principal investigators for Healthy Life Trajectory Initiative South Africa. SAN, CD, LW, AP and SL wrote the initial protocol draft. CMS, CLD, PA, DGB, ZAB, LB, BC, TC, BF, CG, JH, JJ, HJ, JMJ, KK, AK, EL, NL, MCM, MR, DER, SWS, DS, WS, DMS, MS, ST, MT, ST, SM, and LMR read and contributed to the final version. All authors provided edits and critiqued the manuscript for intellectual content.

Funding
The authors extend our gratitude to the South African Medical Research Council (Strategic Health Innovation Partnerships grant) and the Canadian Institutes of Health Research for funding (grant number: HLS 151553) *Bukhali.*

Trial Sponsor
University of the Witwatersrand, Johannesburg, South Africa; Contact Dr Drennan (robin.drennan@wits.ac.za).

Competing interests
We have no conflicts of interest to declare. The HeLTI Council of funders and WHO require all Principal Investigators to complete annual Declarations of Interest and to commit to having no direct or indirect relations with the tobacco, arms and food industries during the course of the trial.

Patient consent for publication Not required.

For peer review only

Provenance and peer review Not commissioned; peer-reviewed for ethical and funding approval prior to submission.

References

1. Wang H, Naghavi M, Allen C, *et al*. Global, regional, and national life expectancy, all-cause mortality, and cause-specific mortality for 249 causes of death, 1980-2015: a systematic analysis for the global burden of disease study 2015. *Lancet* 2016;388:1459–544.
2. World Health Organisation. https://www.who.int/news-room/spotlight/10-global-health-issues-to-track-in-2021 Accessed November 2021
3. Wells JC, Sawaya AL, Wibaek R, *et al*. The double burden of malnutrition: aetiological pathways and consequences for health. *Lancet* 2020;395:75-88.
4. World Obesity Federation. *Atlas of Childhood Obesity.* 2019, London.
5. Nagpal TS, Souza SCS, Moffat M, *et al*. Does prepregnancy weight change have an effect on subsequent pregnancy health outcomes? A systematic review and meta-analysis. *Obes Rev* 2021; e13324.
6. Woo Baidal JA, Locks LM, Cheng ER, *et al*. Risk factors for childhood obesity in the first 1,000 days: a systematic review. *Am J Prev Med* 2016;50:761–79
7. Heslehurst N, Vieira R, Akhter Z, *et al*. The association between maternal body mass index and child obesity: A systematic review and meta-analysis. *PLoS Med* 2019;16:e1002817.
8. Dean SV, Lassi ZS, Imam AM, *et al*. Preconception care: nutritional risks and interventions. *Reprod Health* 2014;11:S3.
9. Dean SV, Lassi ZS, Imam AM, *et al*. Preconception care: closing the gap in the continuum of care to accelerate improvements in maternal, newborn and child health. *Reprod Health* 2014;11:S1
10. Nyati LH, Pettifor JM, Norris SA. The prevalence of malnutrition and growth percentiles for urban South African children. *BMC Public Health* 2019;19:492.
11. Lundeen EA, Norris SA, Adair LS, *et al*. Sex differences in obesity incidence: 20-year prospective cohort in South Africa. *Pediatr Obes*. 2016;11:75-80.
12. Macaulay S, Ngobeni M, Dunger DB, Norris SA. The prevalence of gestational diabetes mellitus amongst black South African women is a public health concern. *Diabetes Res Clin Pract*. 2018;139:278-287
13. Prioreschi A, Wrottesley SV, Said-Mohamed R, *et al*. Understanding how maternal social and biological factors are related to fetal growth in an urban South African cohort. *J Dev Orig Health Dis.* 2021;12:79-87.
14. Barker M, Dombrowski SU, Colbourn T, *et al*. Intervention strategies to improve nutrition and health behaviours before conception. *Lancet*. 2018; 391:1853-1864.
15. Dennis CL, Marini F, Dick JA, *et al*. Protocol for a randomised trial evaluating a preconception-early childhood telephone-based intervention with tailored e-health resources for women and their partners to optimise growth and development among children in Canada: a Healthy Life Trajectory Initiative (HeLTI Canada). *BMJ Open*. 2021;11:e046311.
16. Wu Y, Marc I, Bouchard L, *et al*. Study protocol for the Sino-Canadian Healthy Life Trajectories Initiative (SCHeLTI): a multicentre, cluster-randomised, parallel-group, superiority trial of a multifaceted community-family-mother-child intervention to prevent childhood overweight and obesity. *BMJ Open*. 2021;11:e045192.
17. Kumaran K, Krishnaveni GV, Suryanarayana KG, *et al*. Protocol for a cluster randomised trial evaluating a multifaceted intervention starting preconceptionally-Early Interventions to Support Trajectories for Healthy Life in India (EINSTEIN): a Healthy Life Trajectories Initiative (HeLTI) Study. *BMJ Open*. 2021;11:e045862.
18. Cohen et al. Material and relational difficulties: The impact of the household environment on the emotional wellbeing of young black women living in Soweto, South Africa. . *Family Issues* 2020, Vol. 41(8) 1307–1332
19. Draper CE, Bosire E, Prioreschi A, *et al*. Urban young women's preferences for intervention strategies to promote physical and mental health preconception: A Healthy Life Trajectories Initiative (HeLTI). *Prev Med Rep* 2019;14:100846

20. Ware LJ, Prioreschi A, Bosire E, *et al*. Environmental, Social, and Structural Constraints for Health Behavior: Perceptions of Young Urban Black Women During the Preconception Period – A Healthy Life Trajectories Initiative. *J Nutr Educ Behav*. 2019; S1499-4046:30178-2
21. Bosire EN et al. Young women's perceptions of life in urban South Africa: Contextualising the preconception knowledge gap. African *J Reproduc Health*. 2021;25:39-49.
22. Prioreschi A, Wrottesley SV, Slemming W, *et al*. A qualitative study reporting maternal perceptions of the importance of play for healthy growth and development in the first two years of life. *BMC Pediatr* 2020 Sep 9;20:428
23. Wrottesley SV, Prioreschi A, Slemming W, *et al*. Maternal perspectives on infant feeding practices in Soweto, South Africa. Public Health Nutr. 2021; 24:3602-3614
24. Silubonde TM, Baumgartner J, Ware LJ, *et al*. Adjusting Haemoglobin Values for Altitude Maximizes Combined Sensitivity and Specificity to Detect Iron Deficiency among Women of Reproductive Age in Johannesburg, South Africa. *Nutrients*. 2020;12:633
25. Ware LJ, Kim AW, Prioreschi A, Nyati LH, Taljaard W, Draper CE, Lye SJ, Norris SA. Social vulnerability, parity and food insecurity in urban South African young women: the healthy life trajectories initiative (HeLTI) study. *J Public Health Policy*. 2021;42:373-389.
26. Prioreschi A, Wrottesley SV, Norris SA. Physical Activity Levels, Food Insecurity and Dietary Behaviours in Women from Soweto, South Africa. J Community Health. 2020 Jun 19
27. Kehoe S, Wrottesley S, Prioreschi A, *et al*. Food insecurity, diet quality and body composition: data from the Healthy Life Trajectories Initiative pilot survey in urban Soweto, South Africa. *Public Health Nutr*. 2021;24:1629-1637
28. Wrottesley SV, Prioreschi A, Kehoe SH, Ward KA, Norris SA. A maternal "mixed, high sugar" dietary pattern is associated with fetal growth. *Matern Child Nutr* 2019;16: e12912
29. Prioreschi A, Wrottesley SV, Said-Mohamed R, *et al*. Understanding how maternal social and biological factors are related to fetal growth in an urban South African cohort. *J Dev Orig Health Dis*. 2020 Feb 17:1-9
30. Wrottesley SV, Prioreschi A, Norris SA. The association between fetal growth and neonatal adiposity in urban South African infants. *Pediatr Obes*. 2021;16:e12737
31. Soepnel L et al. Obesity and adiposity of 3- to 6-year-old children born to mothers with hyperglycemia detected in pregnancy in an urban South African setting. Annals Human Biol. 2020 48:81-92
32. Draper CE, Prioreschi A, Ware LJ, *et al*. Pilot implementation of *Bukhali*: A preconception health trial in South Africa. *SAGE Open Med*. 2020 Jul 7;8
33. Michie S, Richardson M, Johnston M, *et al*. The behavior change technique taxonomy (v1) of 93 hierarchically clustered techniques: building an international consensus for the reporting of behavior change interventions. *Ann Behav Med* 2013;46:81-95.
34. Lawrence W, Black C, Tinati T, *et al*. 'Making every contact count': Evaluation of the impact of an intervention to train health and social care practitioners in skills to support health behaviour change. *J Health Psychol*. 2016;21:138-51.
35. Lawrence W, Vogel C, Strömmer S, *et al*. How can we best use opportunities provided by routine maternity care to engage women in improving their diets and health? *Matern Child Nutr* 2020;16:e12900.
36. Adam LM, Jarman M, Barker M *et al*. Use of healthy conversation skills to promote healthy diets, physical activity and gestational weight gain: Results from a pilot randomised controlled trial. *Patient Educ Couns* 2020;103:1134-1142.
37. MRC guidance on process evaluation of complex interventions, (https://www.mrc.ac.uk/documents/pdf/mrc-phsrn-process-evaluation-guidance-final/).
38. PA Harris, R Taylor, R Thielke, *et al*, Research electronic data capture (REDCap) – A metadata-driven methodology and workflow process for providing translational research informatics support, *J Biomed Inform 2009;42:377-81*
39. Gaye A, Marcon Y, Isaeva J, *et al*. DataSHIELD: taking the analysis to the data, not the data to the analysis, *Int J Epi* 2014; 43:1929–1944

For peer review only

Legends

Figure 1. Participant flow diagram

Figure 2. Pathways to impact model that informed the intervention development

Figure 3. Impact of outcomes in a multi-phase trial and intervention components

For peer review only

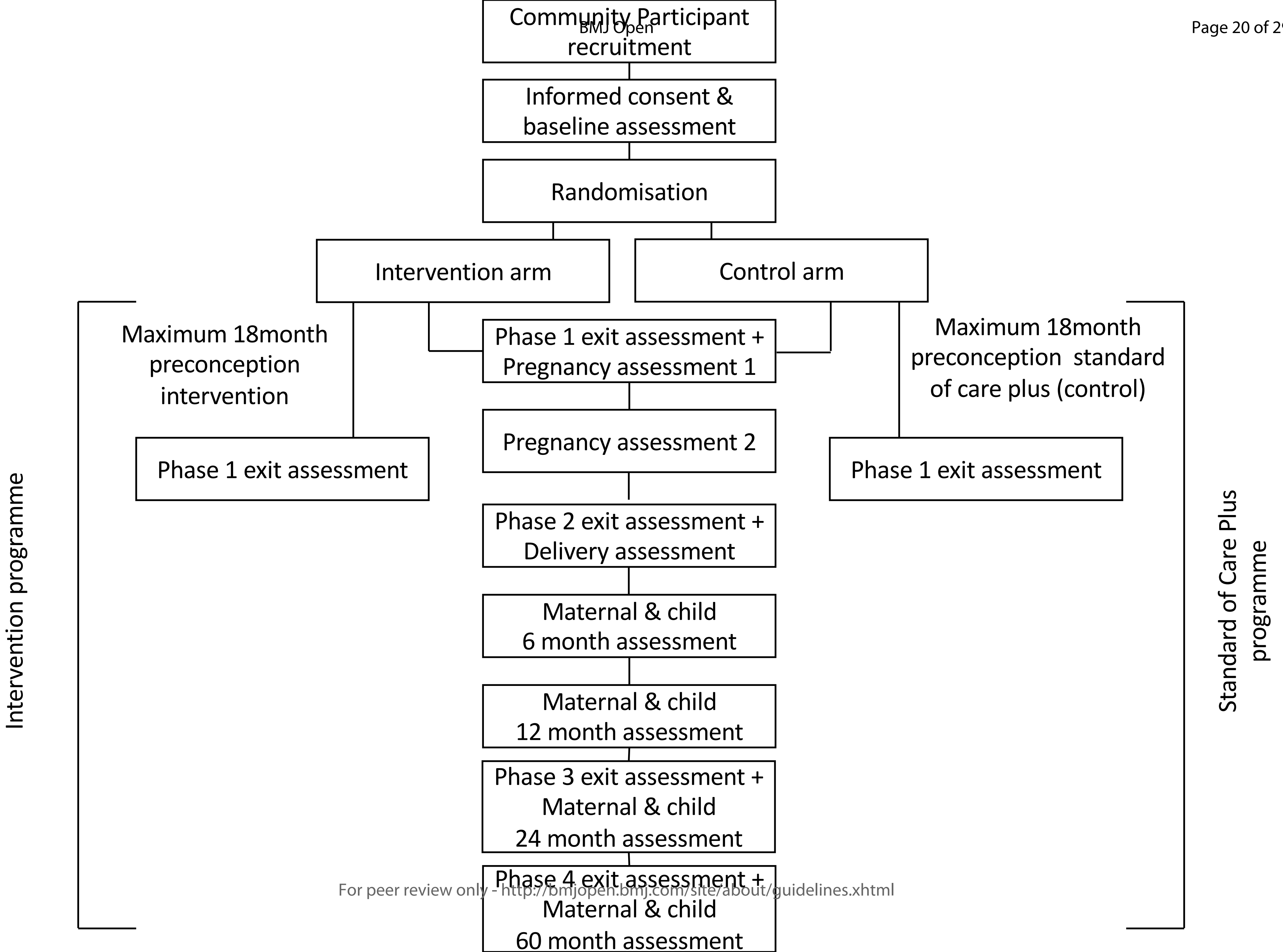
Community Participant recruitment
Informed consent & baseline assessment
Randomisation
Intervention arm
Control arm
Maximum 18month preconception intervention
Phase 1 exit assessment
Phase 1 exit assessment + Pregnancy assessment 1
Maximum 18month preconception standard of care plus (control)
Phase 1 exit assessment
Pregnancy assessment 2
Phase 2 exit assessment + Delivery assessment
Maternal & child 6 month assessment
Maternal & child 12 month assessment
Phase 3 exit assessment + Maternal & child 24 month assessment
Phase 4 exit assessment + Maternal & child 60 month assessment
Intervention programme
Standard of Care Plus programme

Logic model for programme of intervention to improve physical and mental health in women

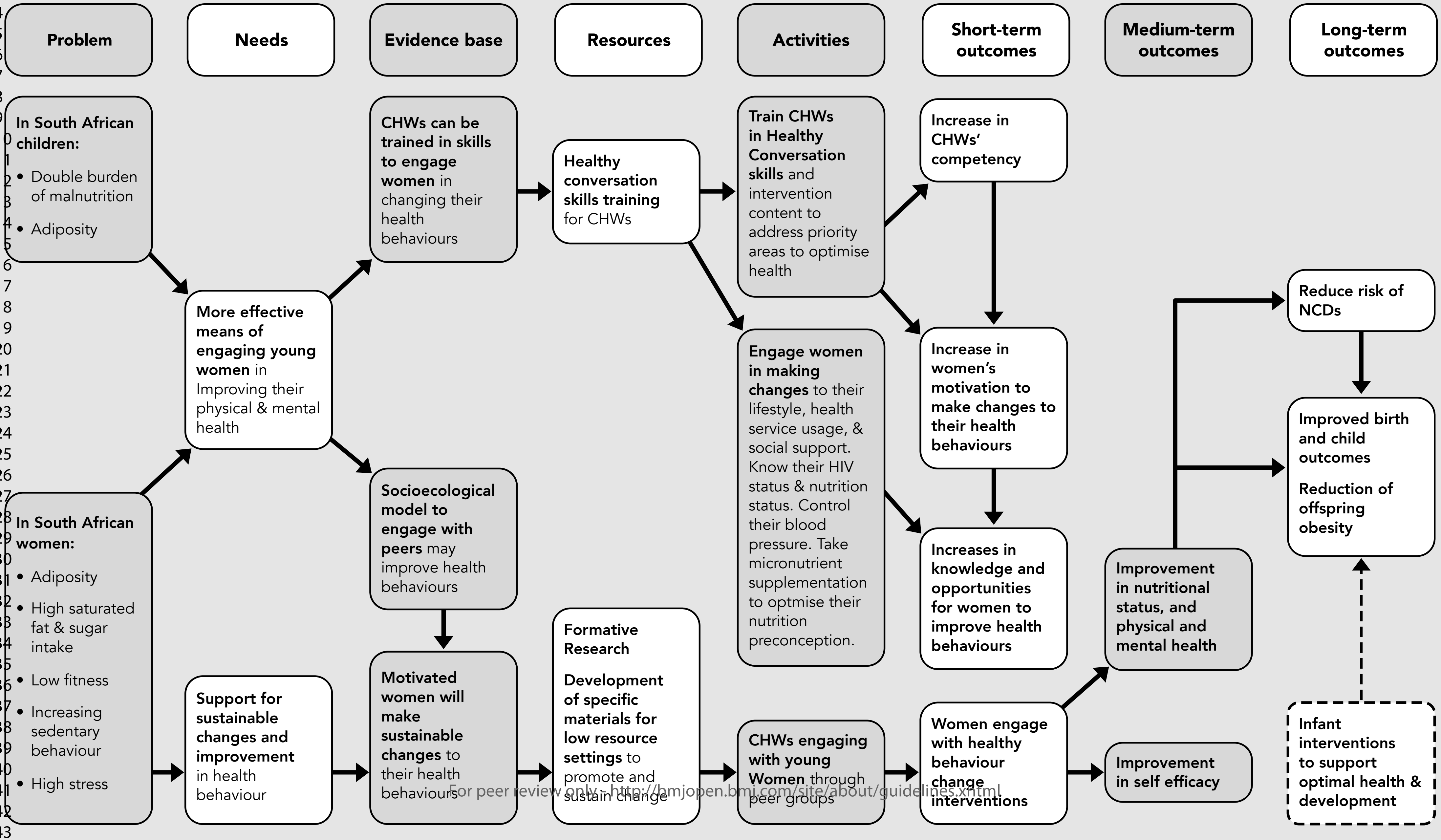

For peer review only - http://bmjopen.bmj.com/site/about/guidelines.xhtml

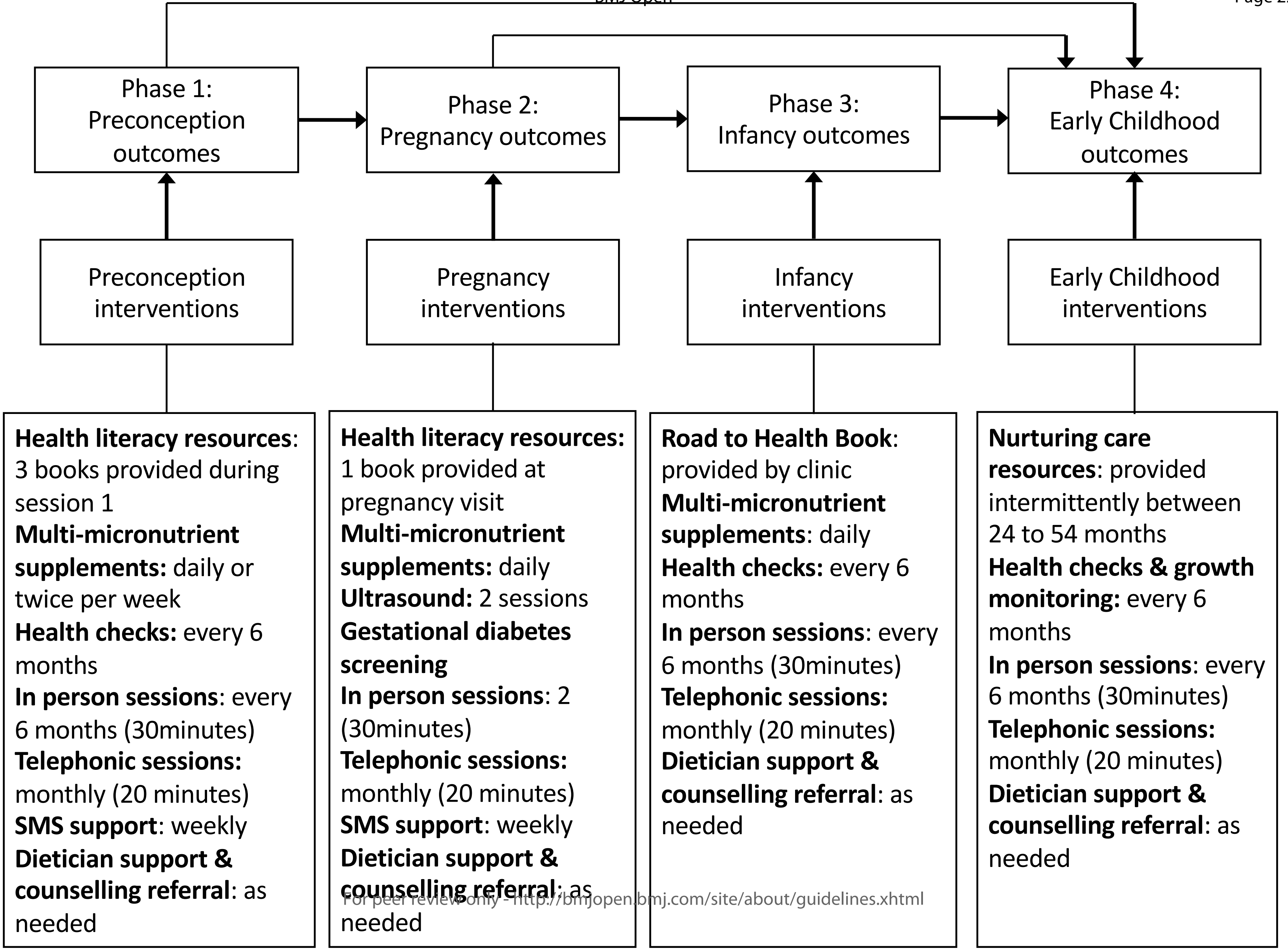

For peer review only - http://bmjopen.bmj.com/site/about/guidelines.xhtml

Table 1: World Health Organization Trial Registration Data Set

Data category	Information
Primary registry and trial identifying number	PACTR201903750173871
Date of registration in primary registry	25 March 2019
Source(s) of monetary or material support	South African Medical Research Council; Canadian Institutes of Health Research
Primary sponsor	University of the Witwatersrand, Johannesburg, South Africa
Contact for public queries	SAN; shane.norris@wits.ac.za
Contact for scientific queries	SAN; shane.norris@wits.ac.za
Public title	HeLTI-SA: a preconception randomised control trial
Countries of recruitment	South Africa
Health condition(s) or problem(s) studied	Child obesity and non-communicable disease risk
Intervention(s)	Active comparator: community health worker, health literacy, behaviour change, dietetic and counselling support; risk-screening health services; multi-micronutrient supplement<br>Placebo comparator: standard of care plus non-health related call centre support
Key inclusion and exclusion criteria	Women aged 18-28 years are eligible, unless they are: (i) diagnosed with type-I diabetes or epilepsy because these require intensive treatment and management priorities; (ii) present with intellectual disability that hinders informed consent, and (iii), not able or willing to provide consent.
Study type	Complex prevention intervention; individual randomisation; research team blind to randomisation; phase II
Date of first enrolment	October 2019
Target sample size	6800 women
Recruitment status	Recruiting
Primary outcome(s)	DXA-derived fat mass index (fat mass/height^2)
Key secondary outcomes	Maternal and child anthropometry, cardiometabolic, and behaviour, and child developmental outcomes

For peer review only

Reporting checklist for protocol of a clinical trial.

Based on the SPIRIT guidelines.

Instructions to authors

Complete this checklist by entering the page numbers from your manuscript where readers will find each of the items listed below.

Your article may not currently address all the items on the checklist. Please modify your text to include the missing information. If you are certain that an item does not apply, please write "n/a" and provide a short explanation.

Upload your completed checklist as an extra file when you submit to a journal.

In your methods section, say that you used the SPIRITreporting guidelines, and cite them as:

Chan A-W, Tetzlaff JM, Gøtzsche PC, Altman DG, Mann H, Berlin J, Dickersin K, Hróbjartsson A, Schulz KF, Parulekar WR, Krleža-Jerić K, Laupacis A, Moher D. SPIRIT 2013 Explanation and Elaboration: Guidance for protocols of clinical trials. BMJ. 2013;346:e7586

For peer review only

		Reporting Item	Page Number
**Administrative information**			
Title	#1	Descriptive title identifying the study design, population, interventions, and, if applicable, trial acronym	1
Trial registration	#2a	Trial identifier and registry name. If not yet registered, name of intended registry	3
Trial registration: data set	#2b	All items from the World Health Organization Trial Registration Data Set	Supp table
Protocol version	#3	Date and version identifier	3
Funding	#4	Sources and types of financial, material, and other support	16
Roles and responsibilities:	#5a	Names, affiliations, and roles of protocol contributors	1; 16

contributorship			
Roles and responsibilities: sponsor contact information	#5b	Name and contact information for the trial sponsor	16
Roles and responsibilities: sponsor and funder	#5c	Role of study sponsor and funders, if any, in study design; collection, management, analysis, and interpretation of data; writing of the report; and the decision to submit the report for publication, including whether they will have ultimate authority over any of these activities	16
Roles and responsibilities: committees	#5d	Composition, roles, and responsibilities of the coordinating centre, steering committee, endpoint adjudication committee, data management team, and other individuals or groups overseeing the trial, if applicable (see Item 21a for data monitoring committee)	14
**Introduction**			
Background and rationale	#6a	Description of research question and justification for undertaking the trial, including summary of relevant studies (published and unpublished) examining benefits and harms for each intervention	4
Background and rationale: choice of comparators	#6b	Explanation for choice of comparators	8
Objectives	#7	Specific objectives or hypotheses	4
Trial design	#8	Description of trial design including type of trial (eg, parallel group, crossover, factorial, single group), allocation ratio, and framework (eg, superiority, equivalence, non-inferiority, exploratory)	6
**Methods: Participants, interventions, and**			

For peer review only

outcomes

Study setting	#9	Description of study settings (eg, community clinic, academic hospital) and list of countries where data will be collected. Reference to where list of study sites can be obtained	4
Eligibility criteria	#10	Inclusion and exclusion criteria for participants. If applicable, eligibility criteria for study centres and individuals who will perform the interventions (eg, surgeons, psychotherapists)	7
Interventions: description	#11a	Interventions for each group with sufficient detail to allow replication, including how and when they will be administered	8-10
Interventions: modifications	#11b	Criteria for discontinuing or modifying allocated interventions for a given trial participant (eg, drug dose change in response to harms, participant request, or improving / worsening disease)	6
Interventions: adherance	#11c	Strategies to improve adherence to intervention protocols, and any procedures for monitoring adherence (eg, drug tablet return; laboratory tests)	14
Interventions: concomitant care	#11d	Relevant concomitant care and interventions that are permitted or prohibited during the trial	N/A
Outcomes	#12	Primary, secondary, and other outcomes, including the specific measurement variable (eg, systolic blood pressure), analysis metric (eg, change from baseline, final value, time to event), method of aggregation (eg, median, proportion), and time point for each outcome. Explanation of the clinical relevance of chosen efficacy and harm outcomes is strongly recommended	7;12
Participant timeline	#13	Time schedule of enrolment, interventions (including any run-ins and washouts), assessments, and visits for participants. A schematic diagram is highly recommended (see Figure)	8

For peer review only

Sample size	#14	Estimated number of participants needed to achieve study objectives and how it was determined, including clinical and statistical assumptions supporting any sample size calculations	7
Recruitment	#15	Strategies for achieving adequate participant enrolment to reach target sample size	7
**Methods: Assignment of interventions (for controlled trials)**			
Allocation: sequence generation	#16a	Method of generating the allocation sequence (eg, computer-generated random numbers), and list of any factors for stratification. To reduce predictability of a random sequence, details of any planned restriction (eg, blocking) should be provided in a separate document that is unavailable to those who enrol participants or assign interventions	6
Allocation concealment mechanism	#16b	Mechanism of implementing the allocation sequence (eg, central telephone; sequentially numbered, opaque, sealed envelopes), describing any steps to conceal the sequence until interventions are assigned	6
Allocation: implementation	#16c	Who will generate the allocation sequence, who will enrol participants, and who will assign participants to interventions	6
Blinding (masking)	#17a	Who will be blinded after assignment to interventions (eg, trial participants, care providers, outcome assessors, data analysts), and how	6
Blinding (masking): emergency unblinding	#17b	If blinded, circumstances under which unblinding is permissible, and procedure for revealing a participant’s allocated intervention during the trial	14
**Methods: Data collection,**			

For peer review only

**management, and analysis**			
Data collection plan	#18a	Plans for assessment and collection of outcome, baseline, and other trial data, including any related processes to promote data quality (eg, duplicate measurements, training of assessors) and a description of study instruments (eg, questionnaires, laboratory tests) along with their reliability and validity, if known. Reference to where data collection forms can be found, if not in the protocol	10-12
Data collection plan: retention	#18b	Plans to promote participant retention and complete follow-up, including list of any outcome data to be collected for participants who discontinue or deviate from intervention protocols	16
Data management	#19	Plans for data entry, coding, security, and storage, including any related processes to promote data quality (eg, double data entry; range checks for data values). Reference to where details of data management procedures can be found, if not in the protocol	10
Statistics: outcomes	#20a	Statistical methods for analysing primary and secondary outcomes. Reference to where other details of the statistical analysis plan can be found, if not in the protocol	11
Statistics: additional analyses	#20b	Methods for any additional analyses (eg, subgroup and adjusted analyses)	11
Statistics: analysis population and missing data	#20c	Definition of analysis population relating to protocol non-adherence (eg, as randomised analysis), and any statistical methods to handle missing data (eg, multiple imputation)	11
**Methods: Monitoring**			
Data monitoring: formal committee	#21a	Composition of data monitoring committee (DMC); summary of its role and reporting structure;	14

For peer review only

		statement of whether it is independent from the sponsor and competing interests; and reference to where further details about its charter can be found, if not in the protocol. Alternatively, an explanation of why a DMC is not needed	
Data monitoring: interim analysis	#21b	Description of any interim analyses and stopping guidelines, including who will have access to these interim results and make the final decision to terminate the trial	11
Harms	#22	Plans for collecting, assessing, reporting, and managing solicited and spontaneously reported adverse events and other unintended effects of trial interventions or trial conduct	14
Auditing	#23	Frequency and procedures for auditing trial conduct, if any, and whether the process will be independent from investigators and the sponsor	14
**Ethics and dissemination**			
Research ethics approval	#24	Plans for seeking research ethics committee / institutional review board (REC / IRB) approval	3
Protocol amendments	#25	Plans for communicating important protocol modifications (eg, changes to eligibility criteria, outcomes, analyses) to relevant parties (eg, investigators, REC / IRBs, trial participants, trial registries, journals, regulators)	3
Consent or assent	#26a	Who will obtain informed consent or assent from potential trial participants or authorised surrogates, and how (see Item 32)	3
Consent or assent: ancillary studies	#26b	Additional consent provisions for collection and use of participant data and biological specimens in ancillary studies, if applicable	10
Confidentiality	#27	How personal information about potential and enrolled participants will be collected, shared, and	10

For peer review only

		maintained in order to protect confidentiality before, during, and after the trial	
Declaration of interests	#28	Financial and other competing interests for principal investigators for the overall trial and each study site	13
Data access	#29	Statement of who will have access to the final trial dataset, and disclosure of contractual agreements that limit such access for investigators	16
Ancillary and post trial care	#30	Provisions, if any, for ancillary and post-trial care, and for compensation to those who suffer harm from trial participation	N/A
Dissemination policy: trial results	#31a	Plans for investigators and sponsor to communicate trial results to participants, healthcare professionals, the public, and other relevant groups (eg, via publication, reporting in results databases, or other data sharing arrangements), including any publication restrictions	6;14
Dissemination policy: authorship	#31b	Authorship eligibility guidelines and any intended use of professional writers	N/A
Dissemination policy: reproducible research	#31c	Plans, if any, for granting public access to the full protocol, participant-level dataset, and statistical code	14
**Appendices**			
Informed consent materials	#32	Model consent form and other related documentation given to participants and authorised surrogates	Supplementary document
Biological specimens	#33	Plans for collection, laboratory evaluation, and storage of biological specimens for genetic or molecular analysis in the current trial and for future use in ancillary studies, if applicable	Table 3

The SPIRIT Explanation and Elaboration paper is distributed under the terms of the Creative Commons Attribution License CC-BY-NC. This checklist was completed on 04. December 2021 using https://www.goodreports.org/, a tool made by the EQUATOR Network in collaboration with Penelope.ai

For peer review only
